# Supplementary material for: Clinical features and molecular mechanisms of RP1L1 variants causing occult macular dystrophy
Source: HGG Adv. 2025 May 30;6(3):100461. doi: 10.1016/j.xhgg.2025.100461 (PMC12206149; doi:10.1016/j.xhgg.2025.100461)
Supplement: Document S1. Figures S1–S7, Tables S1–S4, and the Members of the Japan Eye Genetics Consortium [file mmc1.pdf]

HGGA, Volume *6*

Supplemental information

Clinical features and molecular mechanisms of *RP1L1* variants causing occult macular dystrophy

Yang Pan, Daisuke Iejima, Kazutoshi Yoshitake, Kazushige Tsunoda, Takeshi Iwata, and on behalf of the Japan Eye Genetics Consortium

Supplemental material

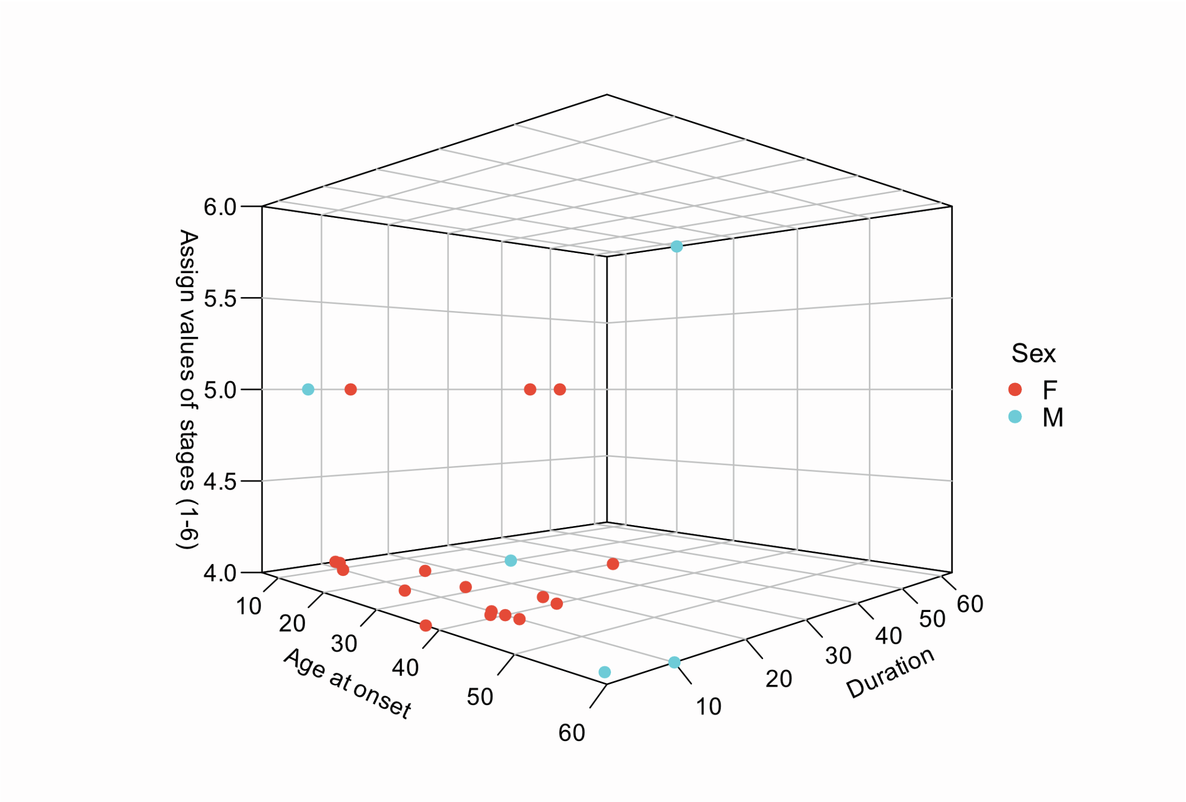

Figure S1. Correlation analysis in a 3D scatter plot

The 3D scatter plot shows the correlation between age at onset and OMD stage, as well as the correlation between duration and OMD stage. The female data points are represented by red dots, whereas the male data points are represented by blue dots.

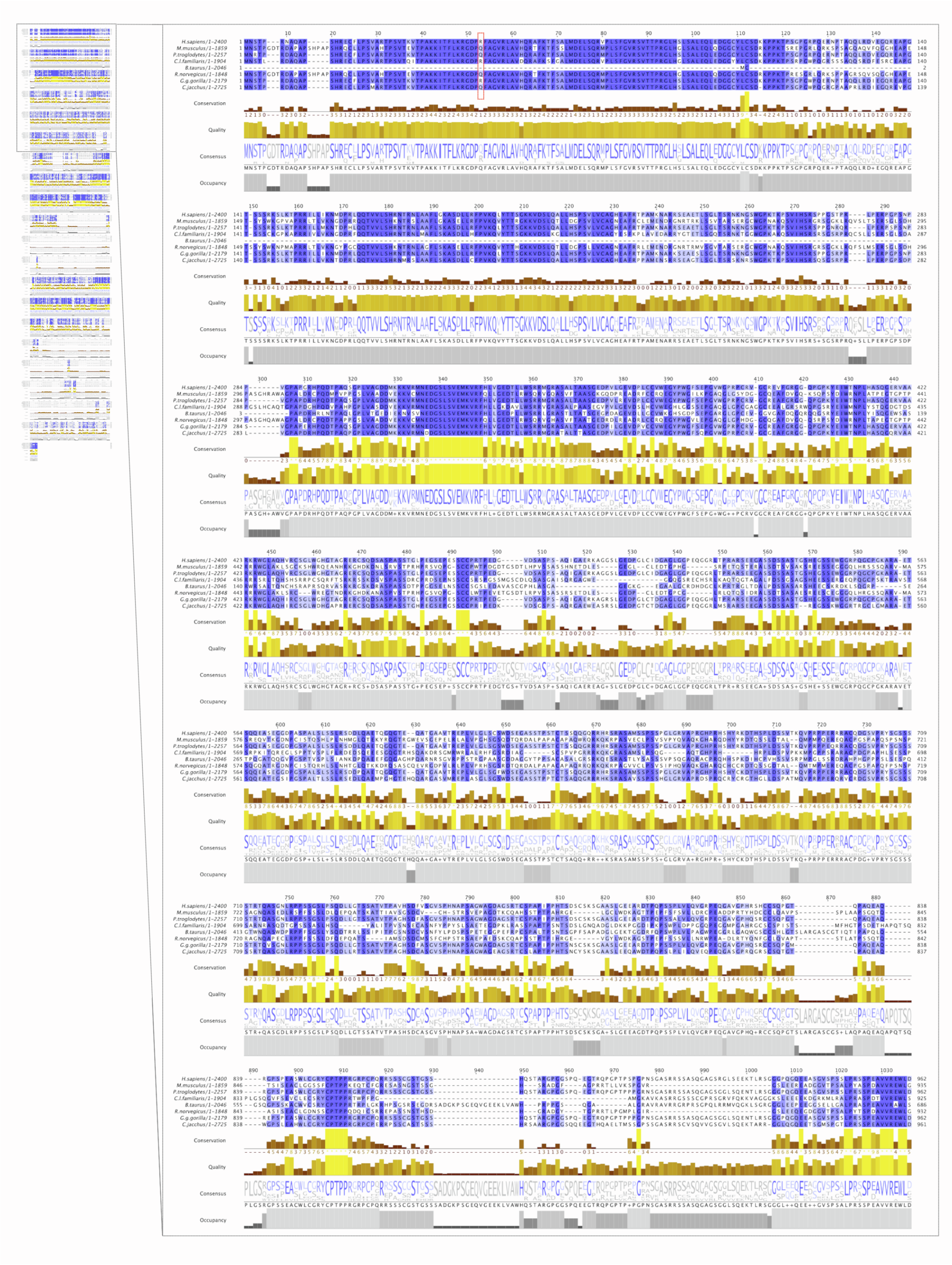

Figure S2. Multiple sequence alignment for RP1L1 in Jalview

The position of the variant is shown below. Histograms show the degree of conservation at each residue. The human RP1L1 R45 is indicated by the red box.

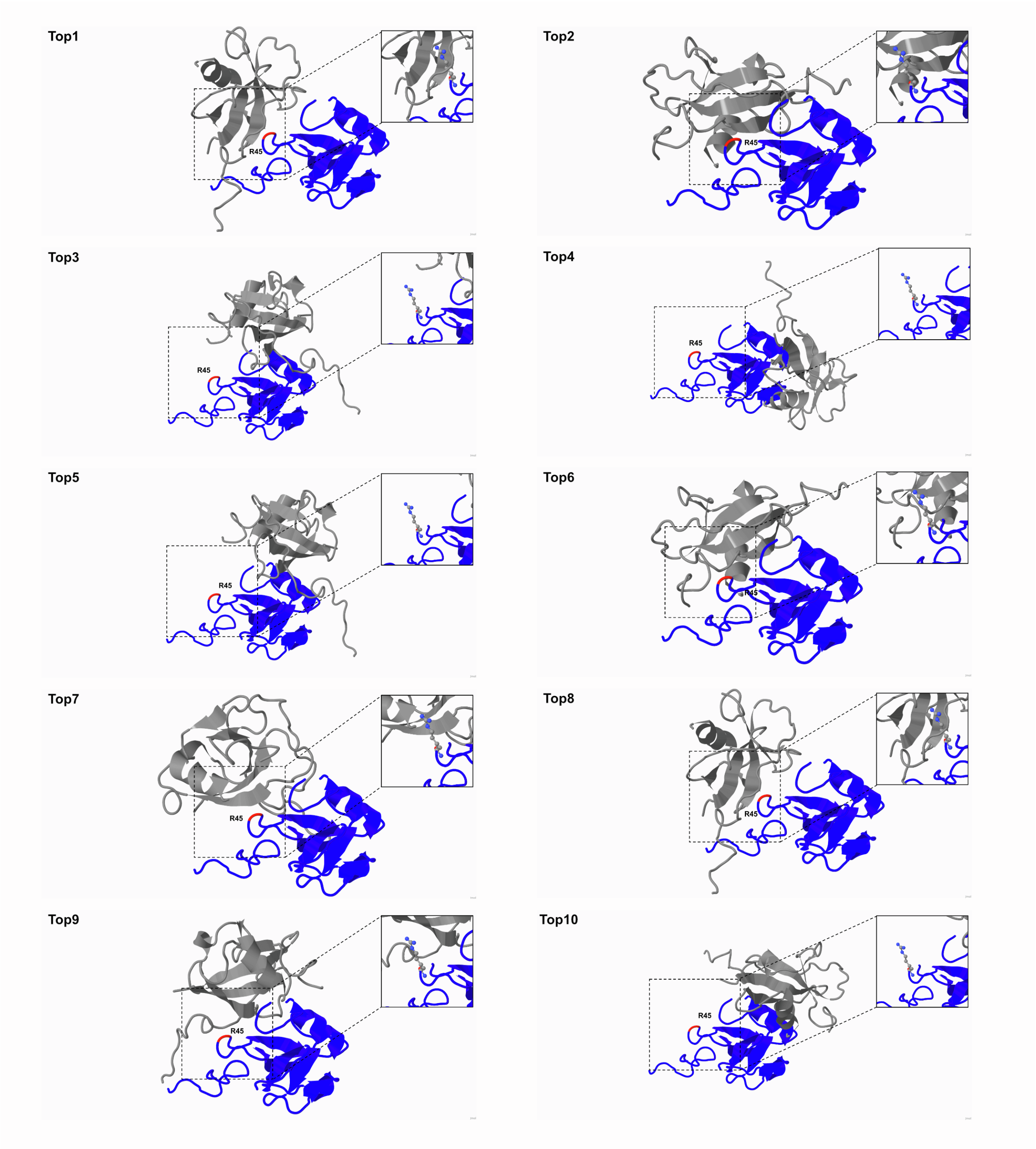

Figure S3. Top 10 docking predictions for RP1L1 and RP1 binding

The Phyre2 server generated RP1L1 (blue) and RP1 (gray) protein structures. The protein structures were visualized with Jmol (https://jmol.sourceforge.net).

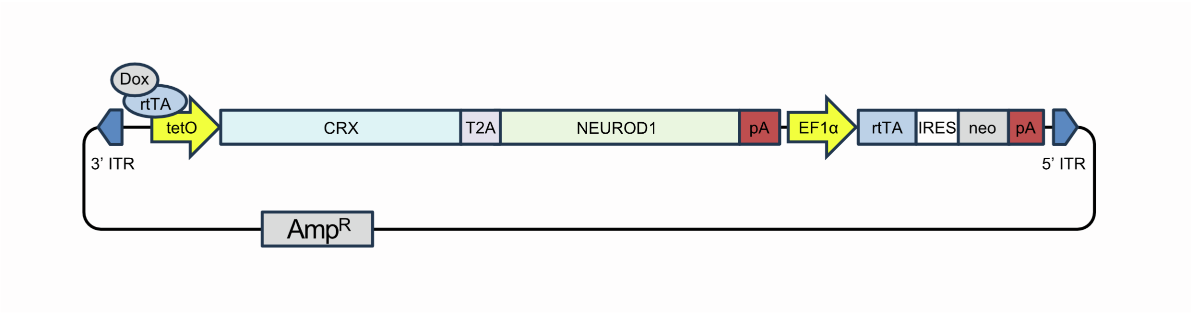

Figure S4. Schematic representations of the polycistronic piggyBac vector for CRX and NEUROD1 introduction into iPSCs

ITR, inverted terminal repeats; Dox, doxycycline; rtTA, reverse tetracycline transactivator; tetO, dox-responsive promoter; T2A, self-cleaving 2A peptide; pA, polyadenylation signal; EF1α, elongation factor 1α; IRES, internal ribosome entry site; neo, neomycin resistance gene.

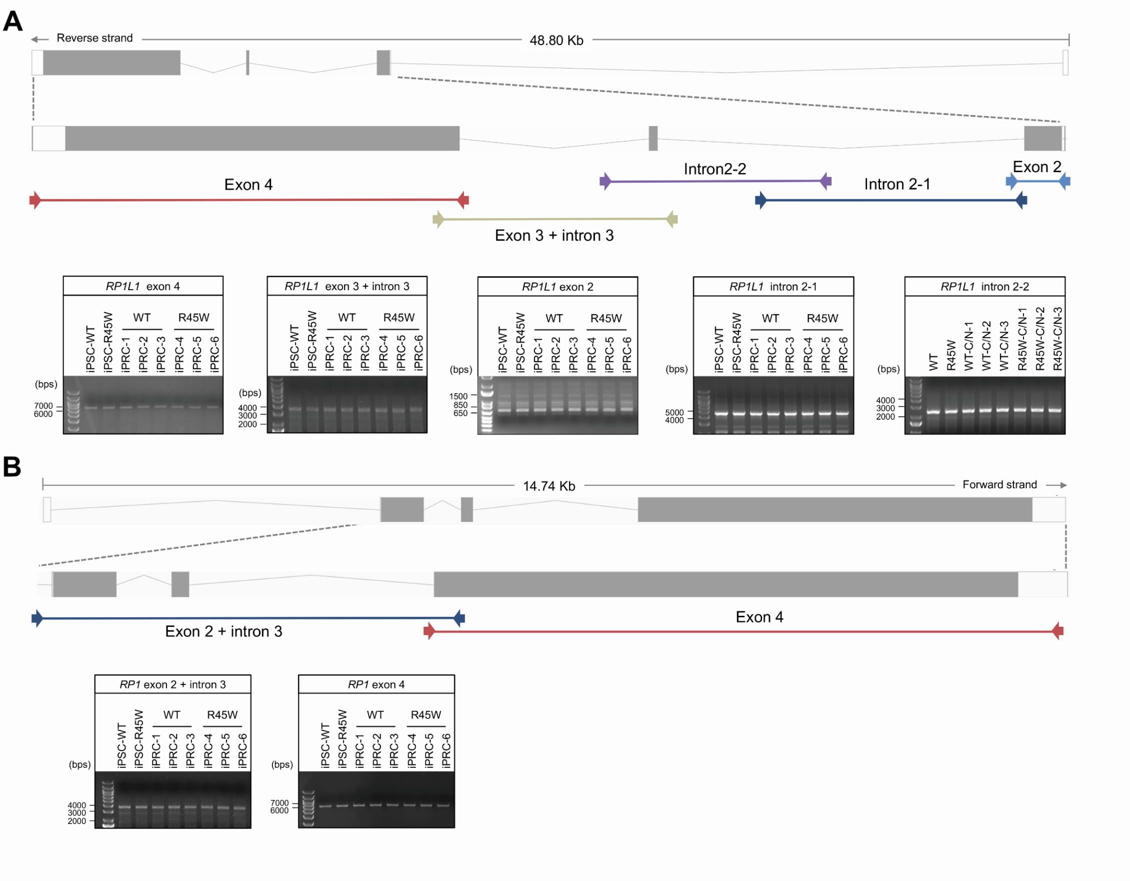

Figure S5. Confirmation of *RP1L1* and *RP1* gene integrity via endpoint PCR

Gel electrophoresis images showing the PCR products of *RP1L1* (A) and *RP1* (B) from iPSCs (induced pluripotent stem cells) and iPRCs (iPSC-induced photoreceptor-like cells). The integrity of the RP1L1 and RP1 genes was verified. The arrows highlight the positions of the primers utilized in the PCR reactions.

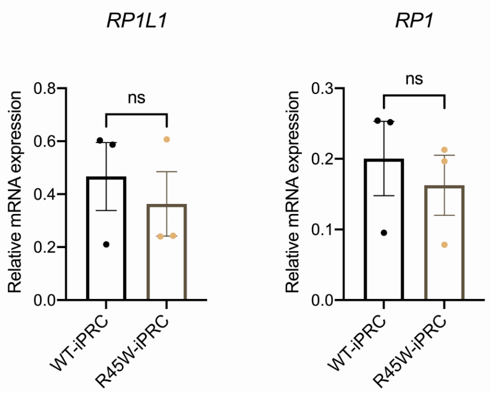

Figure S6. ***RP1L1*** **and** ***RP1*** **transcription in iPRCs**

The transcription levels of *RP1L1* and *RP1* in iPRCs were analyzed via RT–qPCR. The mRNA levels were normalized to those of *GAPDH* and measured in quadruplicate. The data are presented as the means ± SEMs (standard error of the mean). Statistical significance was assessed via Student's *t* test, with nonsignificant results indicated as "ns" (p >= 0.05).

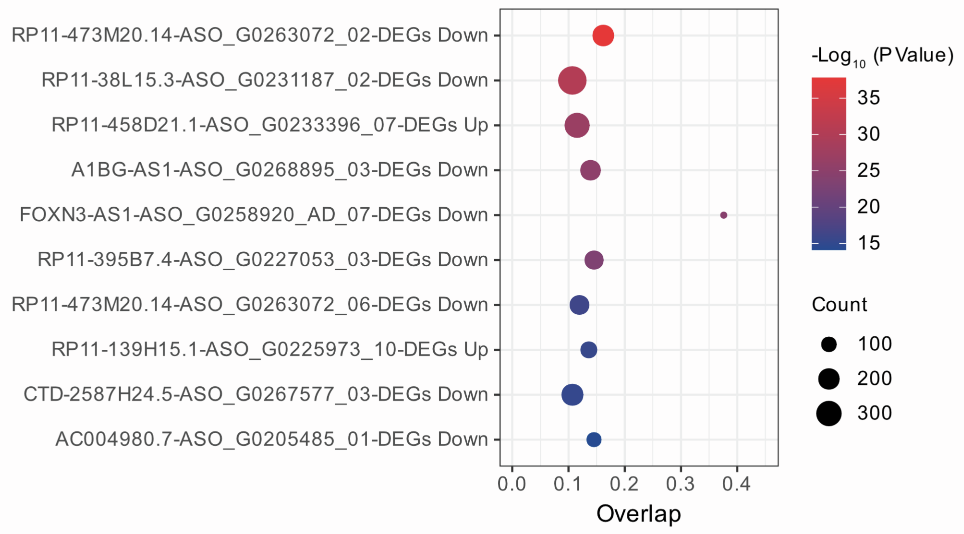

Figure S7. FANTOM6 enrichment analysis

Enrichment analysis was conducted on 1242 DEGs identified in R45W-iPRCs via the FANTOM6_lncRNA_KD_DEGs library. This analysis aimed to uncover associations between R45W and other long noncoding RNAs.

Table S1: Staging of OMD on the basis of visual symptoms and SD-OCT findings.

Family ID	Patient ID	Sex	Age at onset	Duration	Visual Symptoms		Decimal BCVA		OCT			Stage
					1st	2nd	OD	OS	IZ	EZ	Foveal Bulge	
1	KA-008	F	30	37	Reduced VA	-	0.15	0.15	Extinguished	Blurred	Present	IIb
2	KA-017	F	38	10	Reduced VA	Photophobia	0.1	0.1	Extinguished	Blurred	Present	IIb
3	KA-035	F	25	9	Reduced VA	Photophobia	0.2	0.2	Extinguished	Blurred	Present	IIb
4	KA-067	F	7	12	Reduced VA	Photophobia	0.15	0.15	Extinguished	Blurred	Present	IIb
4	KA-068	F	40	10	Photophobia	Reduced VA	0.1	0.1	Extinguished	Blurred	Present	IIb
5	KA-290	F	11	10	Reduced VA	Photophobia	0.1	0.1	Extinguished	Blurred	Present	IIb
5	KA-313	F	37	18	Reduced VA	Photophobia	0.1	0.1	Extinguished	Blurred	Present	IIb
6	SMOP-P01	F	40	17	Reduced VA	-	0.1	0.4	Extinguished	Blurred	Present	IIb
6	SMOP-P02	F	10	48	Reduced VA	-	0.2	0.15	Extinguished	Blurred	Absent	IIIa
6	SMOP-P03	F	6	12	Reduced VA	-	0.15	0.15	Extinguished	Blurred	Present	IIb
6	SMOP-P06	M	20	63	Reduced VA	-	0.15	0.15	Extinguished	Disrupted	Absent	IIIb
6	KA-014	F	18	17	Reduced VA	Photophobia	0.2	0.2	Extinguished	Blurred	Present	IIb
7	Nagoya-144-0144	F	38	2	Photophobia	Reduced VA	0.4	0.5	Extinguished	Blurred	Present	IIb
8	KINKI-070-0017	F	19	6	Reduced VA	Color vision abnormality	0.2	0.2	Extinguished	Blurred	Absent	IIIa
8	KINKI-070-0018	F	12	40	Reduced VA	-	0.1	0.15	Extinguished	Blurred	Absent	IIIa
8	KINKI-070-0019	M	8	7	Reduced VA	-	0.2	0.2	Extinguished	Blurred	Absent	IIIa
9	KINKI-002-2	F	28	15	Reduced VA	-	0.3	0.4	Extinguished	Blurred	Present	IIb
10	KINKI-030-11	M	60	10	Reduced VA	-	0.1	0.2	Extinguished	Blurred	Present	IIb
10	KINKI-030-14	M	58	4	Reduced VA	-	0.15	0.15	Extinguished	Blurred	Present	IIb
11	Nagoya-003-0003	F	42	10	Reduced VA	-	0.3	0.6	Extinguished	Blurred	Present	IIb

12	Nagoya-007-0007	M	20	29	Reduced VA	-	0.16	0.16	Extinguished	Blurred	Present	IIb
13	Nagoya-040-0040	F	39	9	Reduced VA	-	0.2	0.2	Extinguished	Blurred	Present	IIb

OMD = occult macular dystrophy; SD-OCT= spectral-domain optical coherence tomography; F = female; M = male; VA = visual acuity; OD = right eye; OS = left eye; IZ = interdigitation zone; EZ = ellipsoid zone; BCVA = best corrected visual acuity.

Table S2. Sequencing metrics for *RP1L1* and *RP1* coverage.

Metrics	*RP1L1* (pCMV-HA-N-RP1L1)	*RP1* (pCMV-Myc-N-RP1)
Contig Length (with gaps)	8,371	7,326
Contig Length (without gaps)	7,788	7,208
Average Length/Sequence	802	803
Total Sequence Length	136,368	41,764
Top Strand	75	26
Bottom Strand	95	26
Total	170	52
Average Coverage	16.29	5.7

Table S3: Results of in silico molecular genetic analysis for identified *RP1L1* variants in the pCMV-HA-N-RP1L1 (with or without R45W) plasmid and *RP1* variants in the pCMV-Myc-N-RP1 plasmid.

Nucleotide change		Amino acid change	dbSNP ID	Frequency (GnomAD)	SIFT	Polyphen2	CADD	REVEL	MetaLR	Mutation Assessor
RP1L1	c.501A>G	-	-	-	-	-	-	-	-	-
	c.665A>C	p.222H>P	rs4388421	18.61%	tolerated	possibly damaging	likely benign	likely benign	tolerated	low
	c.1242C>T	-	-	-	-	-	-	-	-	-
	c.1651C>T	p.551R>W	rs372698666	0.00%	tolerated	benign	likely benign	likely benign	tolerated	low
	c.1837C>T	-	-	-	-	-	-	-	-	-
	c.4401G>T	p.1467R>S	rs4840498	98.42%	tolerated	benign	likely benign	likely benign	tolerated	-
	c.4448C>T	p.1483A>V	rs62490855	17.88%	deleterious	benign	likely benign	likely benign	tolerated	-
	c.5530G>A	p.1884A>T	rs773601565	0.00%	tolerated	benign	likely benign	likely benign	tolerated	-
	c.5836C>A	p.1946A>E	rs11785822	92.42%	tolerated	benign	likely benign	likely benign	tolerated	-
	c.5860A>G	p.1954T>A	rs11783478	61.40%	tolerated	benign	likely benign	likely benign	tolerated	-
	c.6723A>G	-	-	-	-	-	-	-	-	-
	c.6853G>A	p.2285G>R	rs55642448	47.84%	tolerated	benign	likely benign	likely benign	tolerated	-
RP1	c.2619G>A	p.872R>H	rs444772	27.61%	deleterious	benign	likely benign	likely benign	tolerated	neutral
	c.5008G>A	p.1670A>T	rs446227	25.94%	tolerated	benign	likely benign	likely benign	tolerated	low
	c.5071T>C	p.1691S>P	rs414352	27.75%	tolerated	benign	likely benign	likely benign	tolerated	neutral
	c.5175A>G	-	-	-	-	-	-	-	-	-

dbSNP = single nucleotide polymorphism database; GnomAD = genome aggregation database.

Table S4. Information on the primers used in the experimental procedures.

Name	Sequence (5' to 3')	Application
RP1L1-R45W-F	TTTGTCCTTCCTTCTGTTCCATC	PR1L1-R45W sequencing
RP1L1-R45W-R	GCTTCTTATCAGAGCAGAGGTAGC	
RP1L1-A-EcoRI-F	ATGAGAATTCGGATGAACAGCACCCCCAGGAATG	RP1L1 cloning
RP1L1-A-R	GCCACAGGTCCTTCGAGATG	
RP1L1-B-F	TGCCAGCAAAGTGAGGTTCAAAGACTCC	
RP1L1-B-Not1-R	ATGCGCGGCCGCCTAGAAATCTAAGTCATCTTGG CCAA	
RP1-Sal1-F	ATGCGTCGACCATGAGTGATACCCCTTCTAC	RP1 cloning
RP1-Not1-R	CCCCGCGGCCGCTTATAAATCTTCTTGTTCTCT	
RP1-seq1R	GACCTCTGTCAGGTGCTGTAGAAA	RP1 sequencing
RP1-seq2F	ACGGCGAGTCCTACCTATGTTC	
RP1-seq2R	TCTCAACTGTCATAGTGCCGTCTT	
RP1-seq3F	GGAAATGCAAAGTCAGAAAGCA	
RP1-seq3R	ACTAAGGTCACACTGCCAATCAC	
RP1-seq4F	ACCTATGGAGCGAAGCAGTAATC	
RP1-seq4R	CATCTGCTGAAATAGGACTGAACC	
RP1-seq5F	CAAGTGCAATAAGTGCTGGTGTT	
RP1-seq5R	TCTTGGAAATCGTGGAATTGAG	
RP1-seq6F	GACAGCAAGCAATAAATTCCAGGT	
RP1-seq6R	GCTAAAGAATTTGCCCTGGTTG	
RP1-seq7F	GAAGTGGCATCTGGGTATTTGAG	
RP1-seq7R	GCAGCCTCTACACTTTGCCTTT	
RP1-seq8F	AATTGCCGGTTTGACAGGAG	
RP1-seq8R	ACTCTGAATGTTGACCGTGGAA	
RP1-seq9F	GATGCTCACAAGGCTACCAACA	
RP1-seq9R	TTAGGCAAAGGCCACAGGAG	
RP1-seq10F	TTCCTGTCAATGTCTGCAATACC	
RP1-seq10R	GTTTCTCCTTCATTGGTCTCCTTT	
RP1-seq11F	GGAGAGCAAGCCACTGAAGAA	
RP1-seq11R	CCCTCACAGCACTAACATCACAC	
RP1-seq12F	GCGAACTTACCCAAGAGAAAGAA	
RP1-seq12R	CCAGCAGAAATAAAGGAATGAGTG	
RP1-seq13F	CGATGGATGAACTCTCCTCTTCA	
RP1-seq13R	CCGCTGAGGTCTTGTGTATTTG	
RP1-seq14F	CAGGGAAGAGAACAATAAAGCAAG	

RP1L1-seq1F	GAGTGCTTCCTGCCCTCTGT	RP1L1 sequencing
RP1L1-seq2F	GCCTGTCCGTGGAGATGAAA	
RP1L1-seq3F	GAGAGATGCAGCCAGGACAG	
RP1L1-seq4F	AGGCAGAGACGCAAGGACAG	
RP1L1-seq5F	CTCTTCCACCCCTTCCACCT	
RP1L1-seq6F	CTCCTCGGGCTCTCTTCCTT	
RP1L1-seq7F	CCTCCTCACCCTTGGTTCTG	
RP1L1-seq8F	TATGAGTTGGCGGACGAGAC	
RP1L1-seq9F	TGACACTGGTCCCCAATCAG	
RP1L1-seq10F	GAAGAAGGGCTGCAAGAAGAG	
RP1L1-seq11F	CACAGAGGAACCCACAGAGC	
RP1L1-seq12F	ATGATGGGCCAAGAGCACAC	
RP1L1-seq13F	CGAAACCTCTCGGCCTTCTC	
RP1L1-seq14F	GCAACCAGAGGTCCCATCAA	
RP1L1-seq15F	GGGGAGACCCAGAAGACAGA	
RP1L1-seq16F	GAGGCAGAAGAGGAGGCACA	
RP1L1-seq17F	GCCCAACCAGAGTTAGAAGGTG	
RP1L1-seq18F	GGGGCCAGTGAAGGTTATGA	
RP1L1-seq19F	AGAGCTGCCCCTGAAAACCT	
RP1L1-seq20F	TGAGGATGAAGCAGAAAGAGACA	
RP1L1-seq21F	CTCGAACCTGGAGCAGTTAGC	
RP1L1-seq22F	CCCCGGAGGCAGAAAAGGAG	
RP1L1-seq23F	AGTCAGAAGGTGCAGAGGCCCAAG	
RP1L1-seq24F	GAGGCCCAGGAGGCTGAAGAGGA	
RP1L1-seq25F	CATCTGGGTGTCCGTGTTACTG	
RP1L1-seq26F	CAACCAGAGGTCCCATCAAA	
RP1L1-seq27F	GGGCATAAGTGAAAGGGGAGA	
RP1L1-seq28F	GCTCTGGGCATGAGGACAAC	
RP1L1-seq29F	GAGGCCCAGGAGGCTGAAG	
RP1L1-seq1R	GATCCCCTCGCTTGAGGAAG	
RP1L1-seq2R	GCAGGGGATTCGTCCAGATT	
RP1L1-seq3R	GGCTGACGAGTCCGAAGAAG	
RP1L1-seq4R	CCTTGCGGTAGTGAGAATGC	
RP1L1-seq5R	AAGGAAGAGAGCCCGAGGAG	
RP1L1-seq6R	CTGTGAGCAGCAGTGGCTTC	
RP1L1-seq7R	CTTCTCCTTGATGCCCCTGA	
RP1L1-seq8R	GGGACACCCTCTCCTGATTG	

RP1L1-seq9R	CACACCAGAGGAGGATGTGG	
RP1L1-seq10R	CCCTCTGCTCCTCACTGTCTCT	
RP1L1-seq11R	AGCCCTTCTCCTCCTGTTTCT	
RP1L1-seq12R	GGTCCAGCAGATCATTGTCC	
RP1L1-seq13R	AGGCTCCAGCACCATCCTAC	
RP1L1-seq14R	ATCCTCACCCTCGTCCACTC	
RP1L1-seq15R	CCCGAGTTTGGGATCTTTGT	
RP1L1-seq16R	CCTGGATCTTGGTCACCTCCT	
RP1L1-seq17R	ACCTTCTGACTCTGGCTCGTC	
RP1L1-seq18R	TCTGCCTCCTGGGCATCTAC	
RP1L1-seq19R	TCAGAAGCCTCCTCAGATTGG	
RP1L1-seq20R	CCAGTGGACTGAACGTTGCTC	
RP1L1-seq21R	GGCGCTGAAGGTCTTAAAGG	
RP1L1-seq22R	GTCCATGAGGGCGCTGAAGGT	
RP1L1-seq23R	GAGGCACGCGCTGGGAGAG	
RP1L1-seq24R	GGTAGCAGCCTCCATCTTCC	
RP1L1-seq25R	GCTTCTTATCAGAGCAGAGGTAGC	
RP1L1-seq26R	GCCTCTTCTTCTTGCTGTCCTT	
RP1L1-seq27R	GCTGGCCTCTGACAATTCCT	
RP1L1-seq28R	TCATCTTCCTGGGAGCCTTT	
RP1L1-seq29R	TGCCTCTGGGGTCTCTACATC	
RP1L1-seq30R	CGTCTGACTCTGGCTGGGCATC	
RP1L1-seq31R	AGCCTCCTGTGCCTCCTCTT	
RP1L1-seq32R	TCTGCCTCCTGGGCATCTACATCT	
RP1L1-seq33R	TCAGAAGCCTCCTCAGATTGG	
RP1L1-seq34R	TCTGCCAGCAGTTGCCCCAAG	
RP1L1-seq35R	TGGAGGAGGAAGGGCCTGTTTG	
RP1L1-seq36R	CTCCAAGTACATGGTCATTT	
PB-CRX-T2A-NeuroD1-CRX-1 F	TGGATCCGGTACCGATGATG	CRX and NEUROD sequencing
PB-CRX-T2A-NeuroD1-CRX-1 R	CTCCAGATGGACACAGTGG	
PB-CRX-T2A-NeuroD1-CRX-2 F	CCCTCCACAGATGTGTGTCC	
PB-CRX-T2A-NeuroD1-CRX-2 R	CTCTGCCCTCCAAGATCTGA	
PB-CRX-T2A-NeuroD1-NEUROD1-1 F	ACCCTGGCCCTATGACCAAA	
PB-CRX-T2A-NeuroD1-NEUROD1-1 R	CCAGGTTGGTGGTGGGTTG	

PB-CRX-T2A-NeuroD1-NEUROD1-2 F	CGCTTTGCAAGGGCTTATCC	
PB-CRX-T2A-NeuroD1-NEUROD1-2 R	CACTTTGTACAAGAAAGCTGGGT	
CRX F	CCAGTGTGGATCTGATGCACCA	RT–qPCR
CRX R	GGTACTGGGTCTTGGCAAACAG	
NEUROD1 F	GGTGCCTTGCTATTCTAAGACGC	
NEUROD1 R	GCAAAGCGTCTGAACGAAGGAG	
RCVRN F	TTCAAGGAGTACGTCATCGCC	
RCVRN R	GATGGTCCCGTTACCGTCC	
RHO F	CACCTCTCTGCATGGATACTTCG	
RHO R	ATGGGCTTACACACCACCAC	
ARR3 F	GCACAAGCTAGGGGACAATG	
ARR3 R	CCAGCCGCACATAGTCTCTC	
RP1L1 F	AAAATGCCAGGAGAAGCGAGGC	
RP1L1 R	AGACCGCGAATGGATCACACTC	
RP1 F	TCTACAGCACCTGACAGAGGTC	
RP1 R	TAAATGGCTCCCTTCCTGCCGC	
RP1L1 intron 2-1 F	ACCAGCGGGAAAAAGGTAGG	PCR
RP1L1 intron 2-1 R	AATCGCACCCACTAAACGGT	
RP11 intron 2-2 F	AGCTACCGTTTAGTGGGTGC	
RP1L1 intron 2-2 R	ACTGTTGCCTGAGACCATCG	
RP1L1 exon 2 F	CCCTCTTCCTCTGCCCTGTT	
RP1L1 exon 2 R	TTTTTCCCGCTGGTCGTGTA	
RP1L1 exon 3 + intron 3 F	CAGGGTAGAGGGGTTTCTGC	
RP1L1 exon 3 + intron 3 R	ATGGATCACACTCGGCTTGG	
RP1L1 exon 4 F	TTCCTCTTCACTGCGTGCC	
RP1L1 exon 4 R	GGCTGACCTCCGATAACCG	
RP1 exon2-intron 3 F	TCTTCTCTAGGTCTCAGCCAAAA	
RP1 exon2-intron 3 R	GAGTAAAAGGTGCCCACATCAAAA	
RP1 exon 4 F	GATGTGGGCACCTTTTACTCTTA	
RP1 exon 4 R	TTGTCCTTAGCACATTTTGATGCC	

Uncropped images

Figure 2E

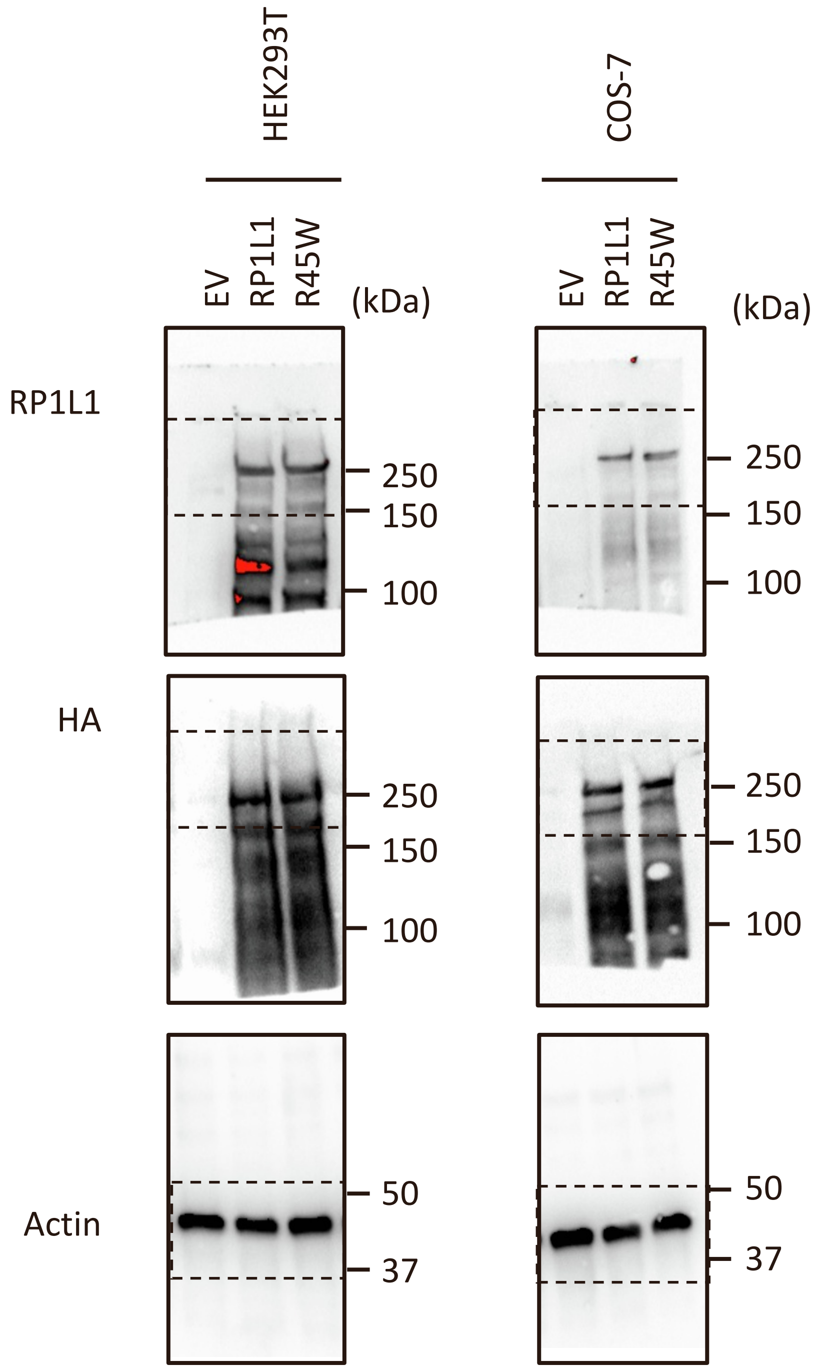

HEK293T
COS-7
EV
RP1L1
R45W
(kDa)
RP1L1
250
150
100
HA
250
150
100
Actin
50
37

Figure 3C

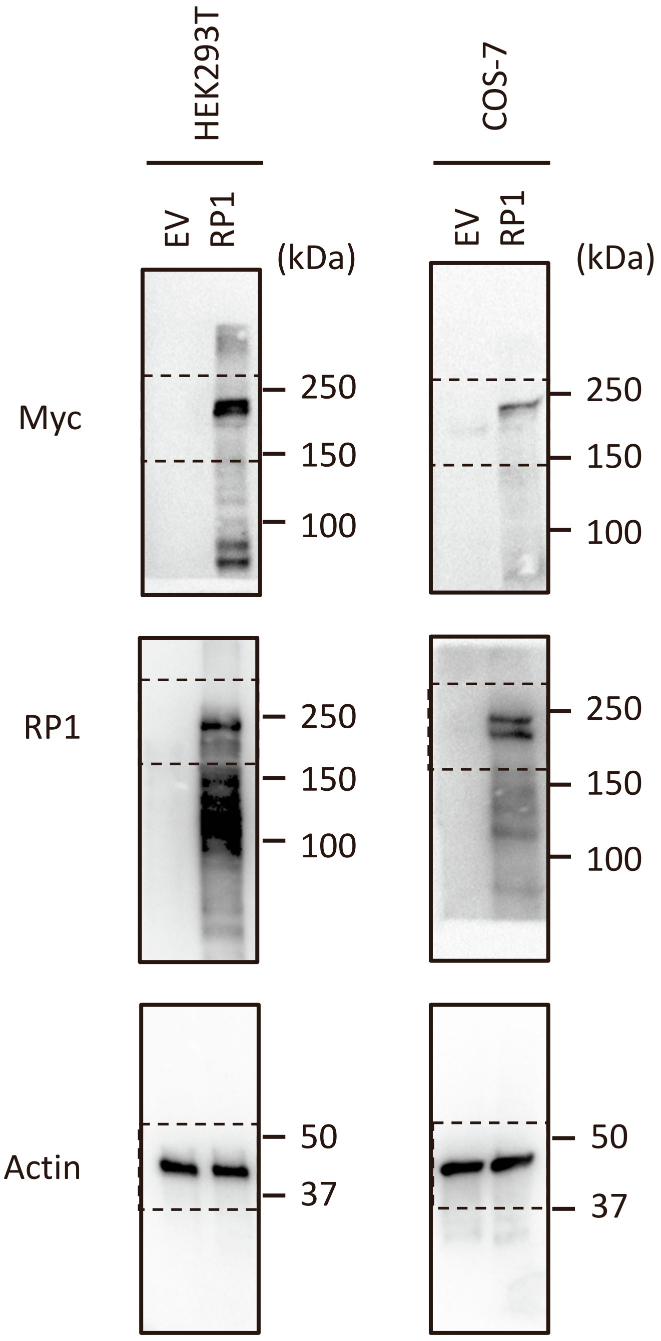

Figure 3D

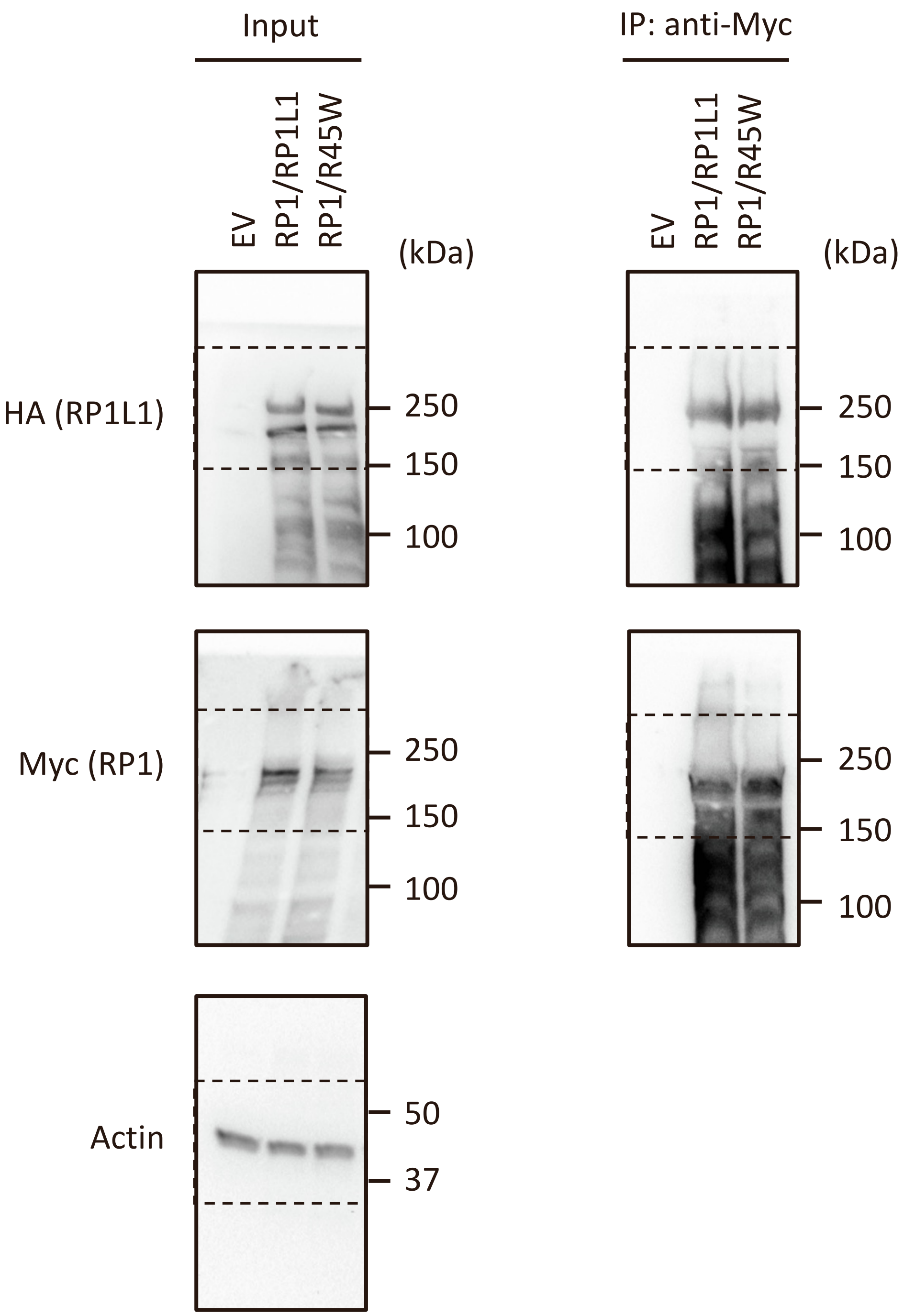

Input
IP: anti-Myc
EV
RP1/RP1L1
RP1/R45W
(kDa)
HA (RP1L1)
250
150
100
Myc (RP1)
250
150
100
Actin
50
37

Figure 4I

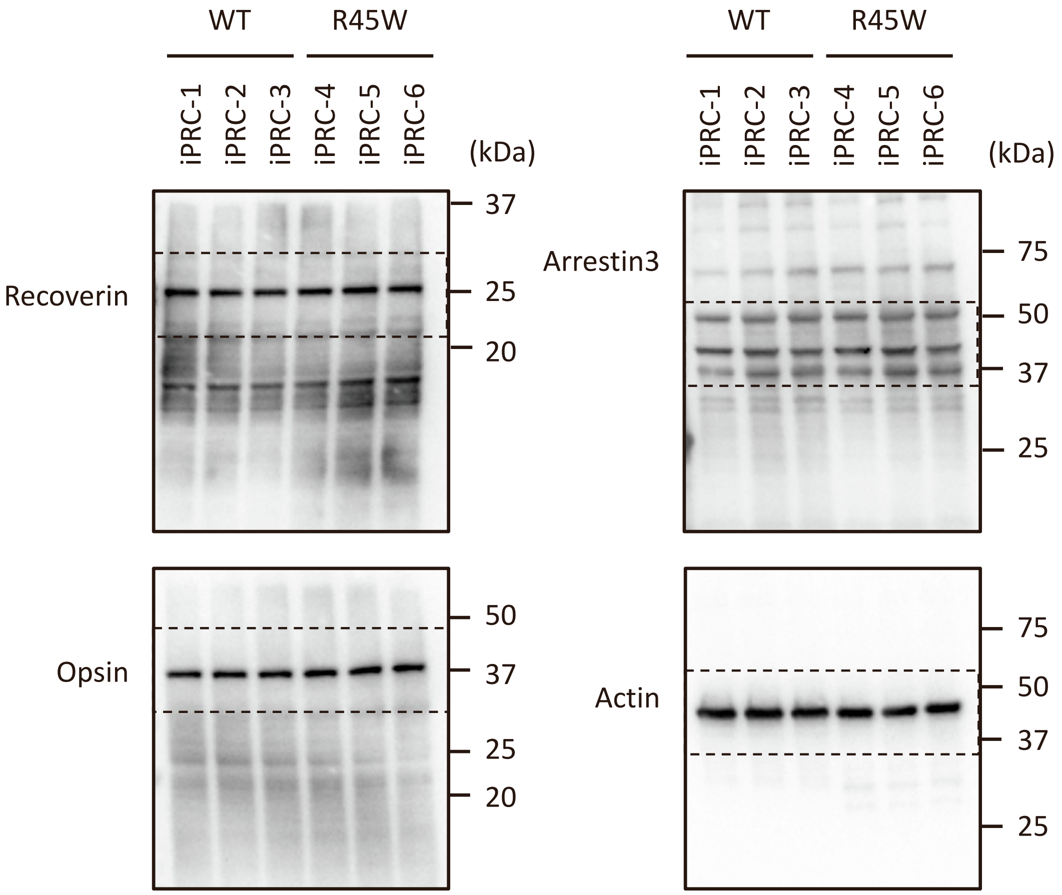
WT
R45W
iPRC-1
iPRC-2
iPRC-3
iPRC-4
iPRC-5
iPRC-6
(kDa)
Recoverin
37
25
20
Arrestin3
75
50
37
25
Opsin
50
37
25
20
Actin
75
50
37
25

Figure 7C

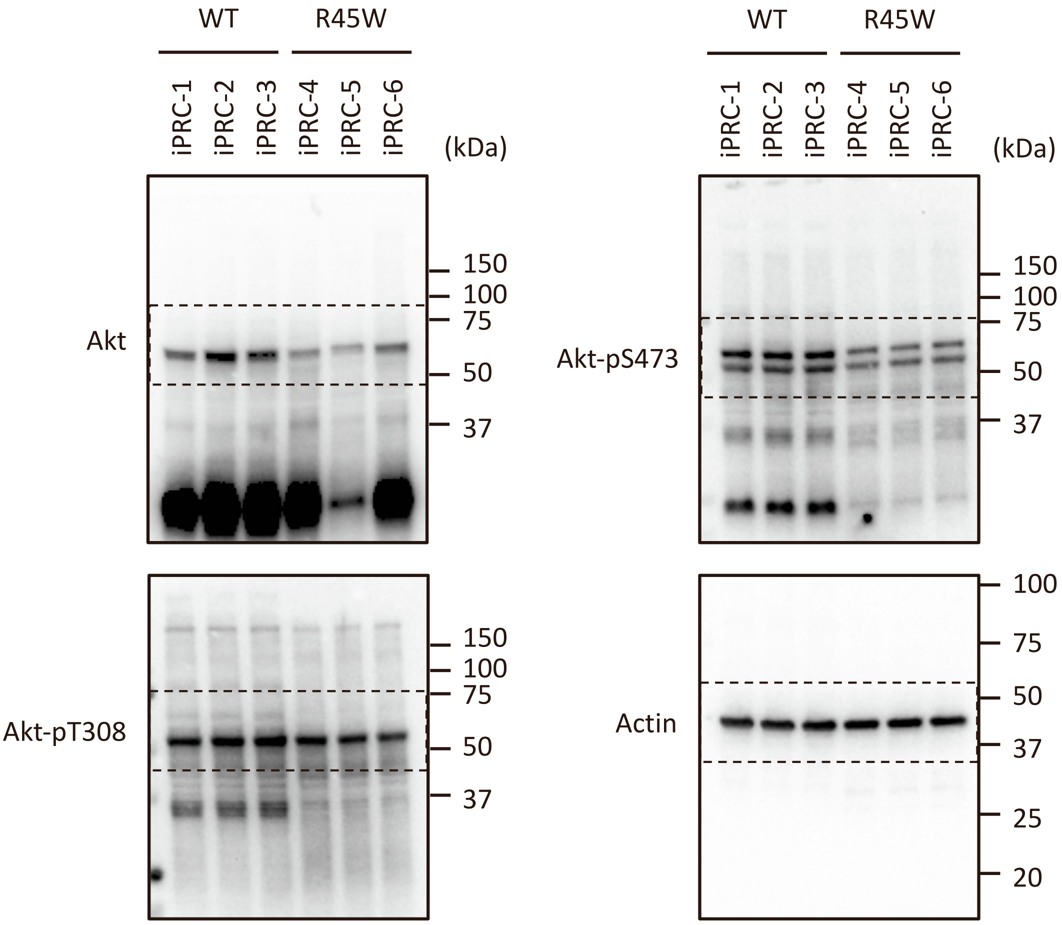
WT
R45W
iPRC-1
iPRC-2
iPRC-3
iPRC-4
iPRC-5
iPRC-6
(kDa)
Akt
150
100
75
50
37
Akt-pT308
Akt-pS473
Actin
25
20

Figure S4

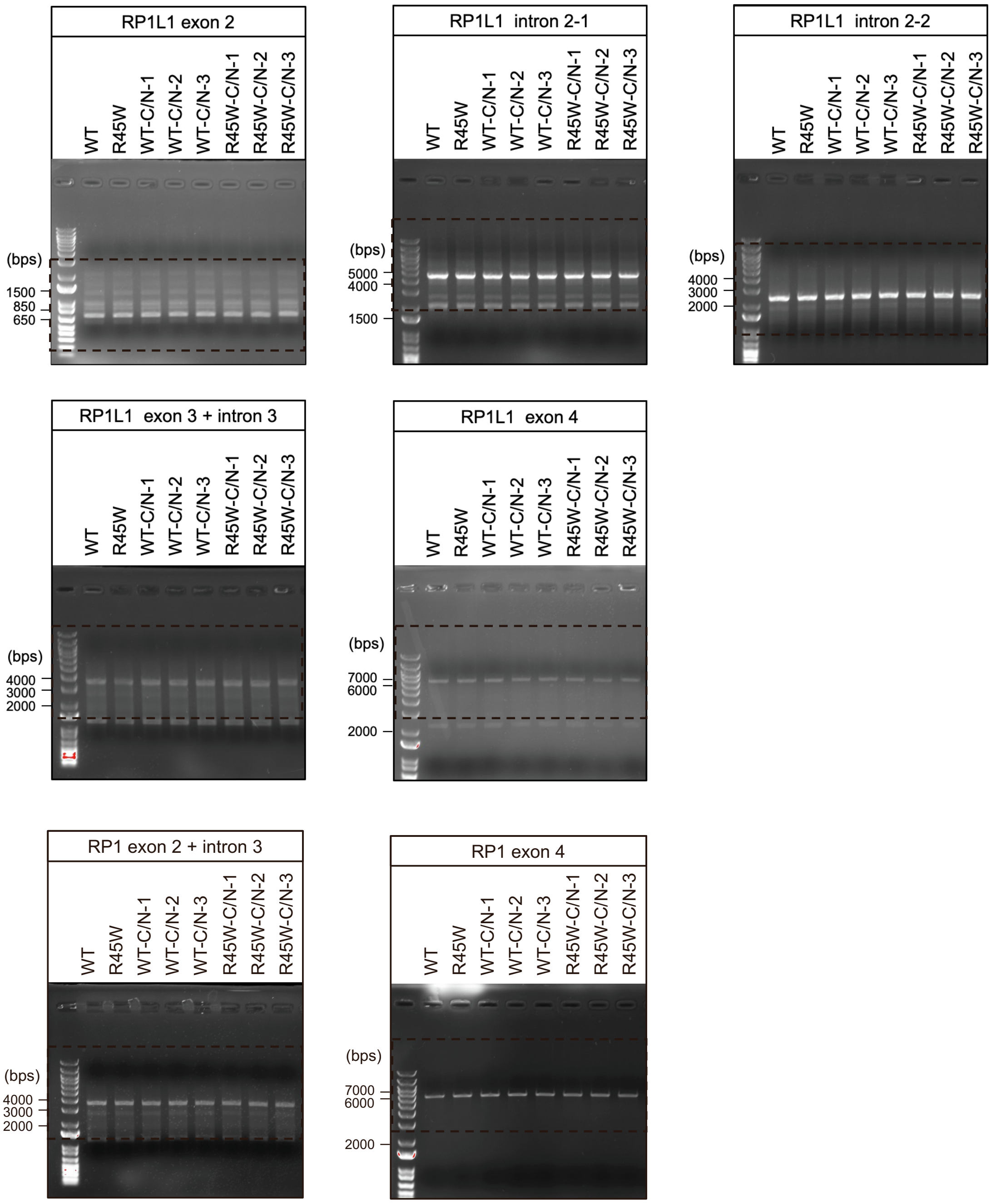
RP1L1 exon 2
WT
R45W
WT-C/N-1
WT-C/N-2
WT-C/N-3
R45W-C/N-1
R45W-C/N-2
R45W-C/N-3
(bps)
1500
850
650
RP1L1 intron 2-1
WT
R45W
WT-C/N-1
WT-C/N-2
WT-C/N-3
R45W-C/N-1
R45W-C/N-2
R45W-C/N-3
(bps)
5000
4000
1500
RP1L1 intron 2-2
WT
R45W
WT-C/N-1
WT-C/N-2
WT-C/N-3
R45W-C/N-1
R45W-C/N-2
R45W-C/N-3
(bps)
4000
3000
2000
RP1L1 exon 3 + intron 3
WT
R45W
WT-C/N-1
WT-C/N-2
WT-C/N-3
R45W-C/N-1
R45W-C/N-2
R45W-C/N-3
(bps)
4000
3000
2000
RP1L1 exon 4
WT
R45W
WT-C/N-1
WT-C/N-2
WT-C/N-3
R45W-C/N-1
R45W-C/N-2
R45W-C/N-3
(bps)
7000
6000
2000
RP1 exon 2 + intron 3
WT
R45W
WT-C/N-1
WT-C/N-2
WT-C/N-3
R45W-C/N-1
R45W-C/N-2
R45W-C/N-3
(bps)
4000
3000
2000
RP1 exon 4
WT
R45W
WT-C/N-1
WT-C/N-2
WT-C/N-3
R45W-C/N-1
R45W-C/N-2
R45W-C/N-3
(bps)
7000
6000
2000

Members of the Japan Eye Genetics Consortium:

Takeshi Iwata, Kazushige Tsunoda, Akiko Suga, Yang Pan, Kazutoshi Yoshitake, Yu Teruyama, Kazuki Kuniyoshi, Takaaki Hayashi, Shinji Ueno, Kei Shinoda, Hiroyuki Kondo, Mineo Kondo, Makoto Nakamura, Kaoru Fujinami, Shuhei Kameya, Nobuhisa Naoi, Yoshihide Hayashizaki, Yasuhiro Murakawa, Hideya Kawaji, Carninci Piero, Toshihide Nishimura, Nobuhiro Shimozawa, Yozo Miyake, Masayuki Horiguchi, Syuichi Yamamoto, Manami Kuze, Atsushi Mizota, Nobuhisa Naoi, Shigeki Machida, Yoshiaki Shimada, Hisashi Fujikado, Yoshihiro Hotta, Masayo Takahashi, Kiyofumi Motiduki, Akira Murakami, Susumu Ishida, Mitsuru Nakazawa, Teruhisa Hatase, Kazuo Tsubota, Akiko Maeda, Atsuhiro Tanigawa, Syuji Yamamoto, Hiroyuki Yamamoto, Tetsuju Sekiryu, Kenji Kashiwagi, Takeo Fukuchi, Atsuhshi Hayashi, Katsuhiro Hosono, Keisuke Mori, Koichi Furuya, Keiichiro Suzuki, Yasuo Yanagi, Natsuko Nakamura.
