# Supplementary material for: Clinical features and molecular mechanisms of RP1L1 variants causing occult macular dystrophy
Source: HGG Adv. 2025 May 30;6(3):100461. doi: 10.1016/j.xhgg.2025.100461 (PMC12206149; doi:10.1016/j.xhgg.2025.100461)
Supplement: Document S2. Article plus supplemental information [file mmc2.pdf]

HGG
Advances

ARTICLE

Clinical features and molecular mechanisms of *RP1L1* variants causing occult macular dystrophy

Yang Pan,[1] Daisuke Iejima,[1] Kazutoshi Yoshitake,[1,2] Kazushige Tsunoda,[3] Takeshi Iwata,[1,5,*] and on behalf of the Japan Eye Genetics Consortium[4]

Summary

Occult macular dystrophy (OMD) is an inherited retinopathy characterized by progressive bilateral vision loss despite normal findings on fundoscopic examination, fluorescein angiography, and full-field electroretinography. Its pathogenesis remains unknown, and no treatments are available. Here, we performed whole-exome sequencing on 133 samples from 78 OMD pedigrees to identify pathogenic variants, using filters for minor allele frequency, function prediction, and retinal expression. We identified the *RP1L1* c.133C>T, p.Arg45Trp (R45W) mutation as the sole pathogenic variant in two families with dominantly inherited OMD. Additionally, we discovered five other potentially pathogenic *RP1L1* variants. Together, these six variants accounted for 33.33% of pedigrees, with R45W being the most prevalent, at 16.6%. The R45W mutation correlated with earlier onset, more severe clinical phenotypes, and abnormal intracellular localization rather than altered expression levels. R45W disrupted the intracellular localization of RP1L1 and RP1, compromising cell viability. In induced photoreceptor-like cells derived from OMD patients carrying R45W, we observed downregulation of the long noncoding RNA *MEG3* and the PI3K/Akt pathway, alongside upregulation of extracellular matrix organization. These findings validate the etiologic role of *RP1L1* and offer insights into the pathogenesis of OMD, thereby facilitating future research and therapeutic development.

Introduction

Inherited retinal diseases (IRDs) comprise a group of heterogeneous disorders leading to vision loss, primarily caused by Mendelian mutations, and afflict more than 2 million people globally.[1] Macular dystrophies, a subset of IRDs, cause significant vision loss that is often due to progressive macular atrophy.[2] Occult macular dystrophy (OMD [MIM: 613587]), or Miyake disease, is an autosomal dominant hereditary retinopathy[3,4] first described in 1989 by Miyake et al., characterized by progressive vision loss despite normal findings on fundoscopic examination, fluorescein angiography, and full-field electroretinography (ERG).[5] Diagnostic procedures such as focal macular ERG and multifocal ERG (mfERG) show severe attenuation of the responses,[5] and spectral-domain optical coherence tomography (SD-OCT) may reveal subtle changes of the photoreceptor layer.[6–8] Consequently, patients with OMD may be misdiagnosed with other disorders that feature low visual acuity and normal fundoscopic findings, such as optic neuropathy of unknown origin, amblyopia, or nonorganic vision loss.[9] This suggests that OMD may be more prevalent than previously thought and highlights the need for better differential diagnosis. No treatments for OMD are available.

The *RP1L1* (NM_178857.6) c.133C>T, p.Arg45Trp (R45W) mutation was first identified in 2010 through linkage disequilibrium (LD) analysis in a Japanese family with OMD and was subsequently detected in two additional OMD-afflicted Japanese families.[4] Subsequent studies have further associated *RP1L1* mutations with OMD,[8,10–15] but they have not incorporated candidate gene filtration or functional validation. Furthermore, LD structures differ between populations, presenting challenges for generalizing LD analysis results across ethnic groups.[16] These interpopulation differences necessitate whole-exome sequencing (WES) or whole-genome sequencing with candidate gene filtration and inheritance pattern analysis to verify and precisely locate pathogenic mutations and thus ensure the accuracy and broad applicability of LD results.

Although human RP1L1 has been reported to localize to the photoreceptor cilium[17,18] and interact with RP1,[17] research into the pathogenic role of *RP1L1* [MIM: 608581] in OMD has been beset by specific challenges. First, *RP1L1* expression is limited to photoreceptors,[4] restricting the availability of suitable human cell lines for research. Second, the *RP1L1* mRNA is extensive, exceeding 7 kb, and contains a large repetitive region that comprises 28 repeats of a polymorphic 16-amino acid sequence encoding an unusually high percentage of glutamine, glycine, and, above all, glutamic acid residues,[18] impeding the construction of a full-length overexpression plasmid. Third, the human RP1L1 protein shows low sequence

[1]Molecular and Cellular Biology Division, National Institute of Sensory Organs, NHO Tokyo Medical Center, Tokyo, Japan; [2]School of Marine Biosciences, Kitasato University, Sagamihara, Kanagawa, Japan; [3]Division of Vision Research, National Institute of Sensory Organs, NHO Tokyo Medical Center, Tokyo, Japan
[4]A list of the members of the Japan Eye Genetics Consortium is available in the supplemental information.
[5]Lead contact
*Correspondence: takeshi.iwata@kankakuki.jp
https://doi.org/10.1016/j.xhgg.2025.100461.
© 2025 The Author(s). Published by Elsevier Inc. on behalf of American Society of Human Genetics.
This is an open access article under the CC BY license (http://creativecommons.org/licenses/by/4.0/).

homology (46.6%) with its murine counterpart, which lacks the complex repeat found in humans, deviating from the average similarity of 85%.[19] Additionally, the absence of a murine macula hinders the *in vivo* investigation of OMD pathology. Furthermore, retinal biopsies are highly invasive and virtually unobtainable, limiting histopathological analysis of retinal dystrophies to postmortem eyes. These factors encumber the investigation of the pathogenic role of RP1L1 in OMD. However, induced pluripotent stem cell (iPSC) technology has enabled the generation of retinal cells and *in vitro* models to study IRDs,[20] enabling the comprehensive assessment of RP1L1 function and OMD pathogenesis.

Here, we performed WES with filtration in 133 samples from 78 families experiencing OMD. We then characterized the identified variants and validated the effects of the most prevalent mutation (R45W) in transfected cells and induced photoreceptor-like cells (iPRCs). R45W disrupted the intracellular localization of R45W and RP1 in co-transfected cells. Additionally, long non-coding RNA (lncRNA) *MEG3* expression and the phosphatidylinositol 3-kinase/protein kinase 3 (PI3K/Akt) pathway were significantly downregulated in iPRCs, providing causal evidence for R45W-associated OMD.

Material and methods

Ethics

Signed informed consent was obtained from all participants. This study adhered to the Declaration of Helsinki and complied with the Health Insurance Portability and Accountability Act. The protocol was approved by the ethics committee of the National Hospital Organization Tokyo Medical Center (R23-090).

Human study participants

Clinical information from 165 OMD patients and 133 DNA samples from OMD families were obtained with the assistance of clinicians from the ophthalmology departments of Tokyo Medical Center, Nagoya University, Kindai University, Jikei University, Mie University, Teikyo University, University of Miyazaki, Aichi University, Gifu University, and Chiba University. The data were analyzed at the Tokyo Medical Center between January 2012 and December 2023.

Diagnostic criteria and clinical staging

The OMD diagnosis was based on a decrease in visual acuity, a normal ophthalmoscopic fundus appearance, normal full-field ERGs, and localized macular dysfunction detected by focal macular or mfERGs.[8] Clinical staging was determined by visual symptoms and microstructural changes observed via SD-OCT. Patients were classified into stages: stage I, with no visual symptoms or minimal structural changes (Ia: minimal changes at the foveal center; Ib: changes in parafoveal structures); stage II, characterized by an extinguished interdigitation zone (IZ) and blurred ellipsoid zone (EZ) (IIa: foveal impairment; IIb: entire macular region affected); and stage III, with an extinguished IZ, absence of the foveal bulge (a dome-shaped structure of the intact EZ in the central fovea observed in SD-OCT imaging[21]), and blurred or disrupted EZ (IIIa: continuous EZ; IIIb: disrupted EZ at the fovea).[8] Each stage was assigned a value from 1 to 6 corresponding to Ia, Ib, IIa, IIb, IIIa, and IIIb, respectively. These values were used to analyze the correlation between subtle photoreceptor layer changes and onset age or disease duration to understand their relationship.

WES data analysis

Genomic DNA from peripheral blood was subjected to WES by Macrogen Japan, as previously described.[22] The reads were aligned to the human reference genome GRCh38 via the Burrows-Wheeler Aligner. Variant calling and analysis were performed via Genome Analysis Toolkit (GATK, version 3.8) following GATK best practices.

We applied a stringent minor allele frequency (MAF) cutoff of <0.00025 for rare dominant alleles across multiple databases: ExAC ($n = 60{,}707$; allele count ≤ 30), gnomAD ($n = 141{,}456$; allele count ≤ 70), HGVD ($n = 1208$; allele count $= 0$), 54KJPN ($n = 54{,}000$; allele count ≤ 27), and our in-house database ($n = 2{,}076$; allele count ≤ 1). Variants were filtered according to inheritance patterns by comparing affected individuals with unaffected controls within the same pedigree. Functional impact was assessed with PROVEAN (modified with ePat), SIFT, PolyPhen-2, and MutationTaster, which require three out of four tools to predict damaging effects. Finally, genes not expressed in the retina (normalized Transcripts Per Million [nTPM] <1 from Human Protein Atlas; accessed December 28, 2023) were excluded.[23] Data processing and filtration were conducted via Python Pandas.

Cloning of RP1L1- and RP1-expressing plasmids

Full-length human *RP1* cDNA (NCBI [NM_006269.2]) was amplified from human retina marathon-ready cDNA (Clontech, catalog no. 639349) via KOD FX *Neo* PCR polymerase (Toyobo, catalog no. KFX-201). Following standard procedures, RP1 cDNA was cloned and inserted into the pCMV-Myc vector (Takara, catalog no. Z5689N) and validated by Sanger sequencing (Table S4).

Owing to its large size and repetitive nature, *RP1L1* cDNA (NCBI [NM_178857.6]) was amplified in two fragments: RP1L1-A (3.4 kb, with *EcoR* I and *Kpn* I sites) and RP1L1-B (3.8 kb, with *Kpn* I and *Not* I sites). These fragments were subsequently cloned and inserted into the pCMV-HA vector (Takara, catalog no. Z5690N). After transformation and purification, the plasmids were digested with *Kpn* I (Takara, catalog no. 1068A) and ligated via a DNA ligation kit (Takara, catalog no. 6023). Mutations were introduced into the expression vector via the KOD-Plus Mutagenesis Kit (Toyobo, catalog no. SMK-101) and validated via Sanger sequencing (Table S4).

Western blot analysis

Plasmids (2 μg) containing pCMV-HA-RP1L1, pCMV-HA-RP1L1-R45W, and pCMV-Myc-RP1 were transfected into HEK293T (RRID: CVCL_0063) and COS-7 cells (American Type Culture Collection [ATCC] catalog no. CRL-1651, RRID: CVCL_0224) using ViaFect transfection reagent (Promega, catalog no. E4981). Forty-eight hours later, the cells were harvested in radioimmunoprecipitation assay (RIPA) buffer supplemented with protease and phosphatase inhibitors (Roche), PMSF, and aprotinin. The samples were subjected to 7.5% Mini-PROTEAN TGX Gel (Bio-Rad, catalog no. 4561024), transferred onto a PVDF membrane via the Transblot Turbo system (Bio-Rad), and probed with Can Get Signal and PVDF Blocking Reagent Set (Toyobo, catalog no.

NKB101/NYPBR). Equal protein loading was confirmed via the use of an anti-actin antibody. Detection was performed via SuperSignal West Femto Maximum Sensitivity Substrate (Thermo Fisher Scientific), and the samples were imaged with a Bio-Rad ChemiDoc XRS+ system. The primary antibodies used were as follows: anti-RP1L1 (1:500; SCB catalog no. sc-87408; RRID: AB_2180481), anti-RP1 (1:500; SCB catalog no. sc-87405; RRID: AB_2180479), and anti-actin (1:4,000; Millipore catalog no. MAB1501; RRID: AB_2223041). The secondary antibodies used were donkey anti-goat immunoglobulin G (IgG) H&L (horseradish peroxidase [HRP]) (1:10,000; Abcam catalog no. ab97110; RRID: AB_10679463) and goat anti-rabbit IgG H&L (HRP) (1:10,000; Abcam catalog no. ab6721; RRID: AB_955447).

Intracellular localization assay

COS-7 (ATCC catalog no. CRL-1651, RRID: CVCL_0224) and 661W (RRID: CVCL_6240) cells were transfected with hemagglutinin (HA)-tagged RP1L1, HA-tagged RP1L1-R45W, HA-tagged RP1L1/Myc-tagged RP1, or HA-tagged RP1L1-R45W/Myc-tagged RP1 vectors using ViaFect transfection reagent (Promega, catalog no. E4981) on 24-well coverslips (Sumitomo Bakelite, catalog no. MS-92132), respectively. After 48 hours, the cells were fixed with 4% paraformaldehyde, permeabilized with 0.3% Triton X-100 in PBS, blocked with protein block serum-free (Dako), and incubated with primary antibodies overnight at 4°C. Secondary antibodies and DAPI were used to detect signals, and the cells were visualized under a confocal fluorescence microscope (Zeiss, catalog no. LSM700). The primary antibodies used were as follows: anti-Myc (1:200; Cell Signaling Technology, catalog no. 2278, RRID: AB_490778) and anti-HA (1:1,000; MBL International, catalog no. M180-3, RRID: AB_10951811). The secondary antibodies used were Alexa Fluor 488 goat anti-rabbit IgG (1:500; Thermo Fisher Scientific, catalog no. A-11034, RRID: AB_2576217) and Alexa Fluor 568 goat anti-mouse IgG (1:500; Thermo Fisher Scientific, catalog no. A-11031, RRID: AB_144696). Nuclear staining was done with DAPI (1:500; Dojindo, catalog no. 340-07971).

RP1L1-RP1 binding prediction

The RP1L1 and RP1 protein structures were predicted via Phyre2.[24] The predicted protein model was used for protein docking analysis via ZDOCK.[25]

Coimmunoprecipitation

HEK293T cells were transfected with Myc-tagged RP1 and HA-tagged RP1L1 or RP1L1-R45W plasmids (10 μg) via Fect transfection reagent (Promega, catalog no. E4981). After 48 h, the cells were harvested in RIPA buffer supplemented with protease and phosphatase inhibitors (Roche), PMSF, and aprotinin. Myc-tagged RP1 and its interactants were extracted via anti-Myc-tag monoclonal antibody-magnetic beads (MBL, catalog no. M047-11) and validated via western blotting (WB). The primary antibodies used were as follows: anti-Myc (1:1,000; Cell Signaling Technology, catalog no. 2278, RRID: AB_490778), anti-HA (1:2,000; MBL International, catalog no. M180-3, RRID: AB_10951811), and anti-actin (1:4,000; Millipore, catalog no. MAB1501; RRID: AB_2223041). The secondary antibodies used were as follows: goat anti-rabbit IgG H&L (HRP) (1:10,000; Abcam, catalog no. ab6721, RRID: AB_955447) and goat anti-mouse IgG H&L (1:10,000; Abcam, catalog no. ab6789, RRID: AB_955439).

Cell viability assay

COS-7 cells were transfected with HA-tagged RP1L1, HA-tagged RP1L1-R45W, HA-tagged RP1L1/Myc-tagged RP1, or HA-tagged RP1L1-R45W/Myc-tagged RP1 vectors via Fect transfection reagent (Promega, catalog no. E4981) in 96-well plates. Cell viability was measured via a Cell Counting Kit-8 (Donjindo, catalog no. CK04) after 24, 48, and 72 h, and the absorbance at 450 nm was measured via an iMark microplate reader (Bio-Rad).

Preparation and maintenance of human iPSCs

Human iPSCs were established from circulating T cells obtained from OMD patients and controls via the Sendai virus. After 5 days of culture with anti-human CD3 (BD Biosciences, catalog no. 555336, RRID: AB_395742) in KBM502 medium (KOHJIN BIO), the cells were transferred to a new plate and treated with the SeV vector mixture (DNAVEC) containing SeV-OCT3/4, SeV-SOX2, SeV-KLF4, and SeV-c-MYC at an MOI of 10. The cells were then transferred onto mitomycin C-inactivated mouse embryonic fibroblast feeder cells. The iPSC medium was changed every other day, and colonies were picked and passaged via collagenase IV (STEMCELL Technologies). Immunofluorescence was used to validate the expression of the following iPSC markers: Oct3/4 (1:200; BD Biosciences, catalog no. 611203, RRID: AB_398737), E-cadherin (1:200; Cell Signaling Technology, catalog no. 3195, RRID: AB_2291471), SSEA4 (1:1,000; Cell Signaling Technology, catalog no. 4755, RRID: AB_1264259), and Nanog (1:200; Cosmo Bio, catalog no. REC-RCAB0003P, RRID: AB_1962353). Sanger sequencing validated the *RP1L1* c.133C>T p.R45W mutation. The primer sequences are listed in Table S4.

Photoceptor-like cell differentiation

iPSCs were differentiated into iPRCs as described previously, with minor modifications.[20] Human iPSCs were expanded in COAT-1-coated (TaKaRa, catalog no. Y30012) 6-well plates in basal medium (Cellartis DEF-CS 500, TaKaRa). The piggyBac vector (PB-CRX [cone-rod homeobox]-T2A-NeuroD1 [neurogenic differentiation 1], a gift from Haruhisa Inoue, RRID: Addgene_194607) was used to introduce CRX and NEUROD1, following selection with neomycin and single-cell cloning. PCR and RT-qPCR were used to validate the expression of these genes. For differentiation, CRX- and NEUROD1-introduced iPSCs were dissociated and plated on iMatrix-511-coated (Nippi, catalog no. 892011) 6-well plates with differentiation medium. Doxycycline was added until 3 days before analysis. Markers of photoreceptor cells were analyzed by qPCR and WB using specific antibodies: anti-arrestin C (1:500; Proteintech, catalog no. 11100-2-AP; RRID: AB_2289959), anti-recoverin (1:500; Proteintech, catalog no. 10073-1-AP; RRID: AB_2178005), and anti-opsin (1:2,000; Sigma-Aldrich, catalog no. O4886; RRID: AB_260838). The primer sequences are listed in Table S4.

RT-qPCR

Total RNA from iPSCs and iPRCs was extracted via the RNeasy Plus Mini Kit (QIAGEN). cDNA was synthesized via ReverTra Ace qPCR RT Master Mix (Toyobo, catalog no. FSQ-201). RT-qPCR was performed with KOD SYBR qPCR Mix (Toyobo, catalog no. QKD-201) on an ABI STEP-One real-time PCR system (Thermo Fisher Scientific). mRNA expression was normalized to that of glyceraldehyde 3-phosphate dehydrogenase (GAPDH). The primers used are listed in Table S4.

RNA sequencing data processing and analysis

Total RNA was extracted from iPRCs via the RNeasy Plus Mini Kit (QIAGEN) and validated for quality and quantity via UV spectrophotometry. RNA with an $OD_{260/280}$ ratio between 1.9 and 2.1 was used for downstream analysis. Sample QC, RNA library preparation, and sequencing were conducted at Amelieff (Tokyo, Japan). RNA integrity was assessed on the Agilent Bioanalyzer 2100 (Agilent Technologies), the highest quality, with a score of >9.8. Sequencing was performed on an Illumina NovaSeq 6000 system with 150-bp paired-end reads.

The RNA sequencing (RNA-seq) data were processed and analyzed via standard bioinformatics pipelines. The raw sequencing reads were quality checked via FastQC (version 0.11.8) and MultiQC (version 1.12), trimmed for adaptors and low-quality bases via Trimmomatic (version 0.39) and PRINSEQ-lite (version 0.20.4), and aligned with the human reference genome (GRCh38) via the STAR aligner (version 2.7.9a).[26–29] FeatureCounts (version 1.6.0) was used to quantify gene expression, and the resulting count matrices were then analyzed via the R package edgeR (version 3.40.0) to determine differentially expressed genes (DEGs) between samples.[30,31] Gene expression was normalized to counts per million (CPM), and genes expressed in fewer than two samples were removed. TMM normalization was applied for DEG analysis. Principal-component analysis was performed via the Euclidean distance with the R_prcomp function and plotted on a two-dimensional plane. Hierarchical clustering was performed for genes expressing more than 1 CPM in at least two samples via R_amap (version 0.8.19) and R_gplots (version 3.1.3). The Euclidean distance was calculated, and the complete linkage method was applied for clustering.

Gene Ontology and Reactome pathway enrichment analyses

To explore the functional implications of these DEGs, Gene Ontology (GO) analysis (biological processes, molecular functions, and cellular components) and Reactome pathway enrichment analysis were performed via the DAVID platform. A false discovery rate (FDR) <0.05 was considered significant. Redundant GO terms were eliminated via REVIGO on the basis of the threshold of medium (0.7). The results were visualized via Hiplot Pro.

Network analysis and hub gene identification

The network analysis and identification of hub genes were performed as previously described.[32] Briefly, the common DEGs were used to construct a protein-protein interaction (PPI) network via the STRING database, with medium confidence (0.4).[33] Hub genes and vital networks were identified via the CytoHubba and MCODE plugins in Cytoscape (version 3.10.2). Furthermore, the GeneMANIA database was utilized to analyze the gene network of the hub genes.[34]

Validation of hub gene expression and regulation of the PI3K/Akt pathway

The expression levels of the hub genes were validated via RT-qPCR, with the mRNA levels normalized to those of GAPDH. The primer sequences are listed in Table S4. To compare the RT-qPCR and RNA-seq fold change (FC) values, the RNA-seq standard error of the mean (SEM) was calculated via the standard error propagation method. The PI3K/Akt pathway was analyzed by WB using the following antibodies: anti-Akt (1:1,000; Cell Signaling Technology, catalog no. 4691; RRID: AB_915783), anti-phospho-Akt (Ser473) (1:2,000; Cell Signaling Technology, catalog no. 4060; RRID: AB_2315049), and anti-phospho-Akt (Thr308) (1:1,000; Cell Signaling Technology, catalog no. 13038; RRID: AB_2629447). The protein bands were quantified via ImageJ (NIH).

Statistics

An unpaired, two-tailed Student's t test or one-way ANOVA followed by Tukey's multiple-comparison test was used for statistical analyses via GraphPad Prism 9. The Mann-Whitney U test was used to determine the significance of the difference in the age of onset between OMD patients and OMD patients carrying RP1L1 R45W. The Shapiro-Wilk test was used to analyze the distributions of onset age, duration, and OMD stage. Levene's test was used to assess whether the variances of multiple groups were approximately equal. Welch's t test was conducted to compare the difference in onset age between general OMD patients and those carrying R45W (R45W-OMD). The Spearman coefficient was used for correlation coefficient analysis among onset age, duration, and OMD stage. Pearson correlation was used for correlation coefficient analysis of the log2(FC) values between the RNA-seq and RT-qPCR validation of the hub genes. All the data are presented as the means ± SEMs. p values less than 0.05 were considered statistically significant.

Results

OMD clinical characteristics and *RP1L1* pathogenic variant features

In total, 165 individuals with clinical diagnoses of OMD from 117 families were identified from our National Institute of Sensory Organs (NISO) database. Demographic and clinical data are summarized in Table 1. The median age of onset was 34.75 ± 3.63 years. Among them, 47.41% ± 8.23% exhibited an autosomal dominant inheritance pattern.

WES was performed on 133 individuals from 78 OMD families. The workflow used to identify causative mutations is shown in Figure 1A and included the filtration of MAF <0.025%, functional effect prediction, and retinal expression. After filtration, three pedigrees had no remaining candidate genes, and two families retained only one candidate gene, *RP1L1* R45W. By screening all 1,062 candidate genes, we found that *RP1L1* was the most common, occurring in 26 OMD families and accounting for 33.33% of the total. Six candidate pathogenic *RP1L1* (NM_178857.6) mutations were identified: R45W in 13 pedigrees (16.67%), c.3596C>G (p.Ser1199Cys) in 6 pedigrees (7.69%), c.3599G>T (p.Gly1200Val) in 4 pedigrees (5.13%), and c.661G>A (p.Gly221Arg), c.2869G>T (p.Val957Phe), and c.3602T>G (p.Val1201Gly) each in 1 pedigree (1.23%) (Figure 1B).

Clinical characteristics of the 22 affected individuals harboring R45W are provided in Table S1 and summarized in Table 1, with pedigrees presented in Figure 1C (some of these data have been published[4,8,13,35]). The median age of onset was 26.62 ± 6.75 years (range 3–60),

Table 1. Comparison of the clinical features observed in patients with OMD from various studies

	OMD	OMD (*RP1L1* p.Arg45Trp)				
Country (sample number)	Japan ($n = 165$)	Japan ($n = 22$)	China ($n = 8$)	Germany ($n = 11$)	Japan ($n = 41$)	East Asian ($n = 20$)
Age at onset, y (range)	34.75 ± 3.63 (3–77)	26.62 ± 6.75 (3–60)	23.25 ± 12.65 (6–51)	–[a]	27.21 ± 4.77 (3–60)	22.85 ± 8.52 (2–73)
Sex, female (%)	83 (50.30)	17 (77.27)	2 (25.00)	7 (63.63)	27 (65.85)	6 (30.00)
Autosomal dominant, (%)	47.41 ± 8.23	58.87 ± 18.24	50.70 ± 31.25	–	–	80.17 ± 17.50
**Spherical equivalent, diopters**						
OD	−2.56 ± 0.50 (−13.0 to 4.0)	−4.03 ± 1.51 (−13.0 to −0.5)	–	−2.32 ± 2.48 (−10 to 1.5)	–	–
OS	−2.55 ± 0.53 (−13.0 to 4.0)	−3.92 ± 1.56 (−13.0 to −0.5)				
**Best-corrected visual acuity, logMAR**						
OD	0.44 ± 0.06 (−0.18 to 1.52)	0.63 ± 0.14 (−0.08 to 1.05)	0.26 ± 0.14 (0.05–0.5)	0.84 ± 0.19 (0.49–1.3)	0.31 ± 0.10 (0.1–1.5)	0.74 ± 0.14 (−0.08 to 1.22)
OS	0.45 ± 0.06 (−0.18 to 1.70)	0.59 ± 0.13 (−0.08 to 1.0)	0.24 ± 0.15 (0.004–0.5)		0.29 ± 0.09 (0.08–1.5)	0.64 ± 0.15 (−0.08 to 1.1)
Symptoms	Reduced or poor visual acuity, photophobia, night blindness, no symptom	Reduced or poor visual acuity, photophobia, no symptom	Reduced visual acuity, photophobia, red-green deficiency, central scotoma	Poor visual acuity, impaired color discrimination	Reduced visual acuity, photophobia, color vision abnormality, no symptom	Reduced visual acuity, photophobia, frowning eyes, no symptom
Bilateral (%)	80.15 ± 5.77	86.41 ± 13.64	–	–	–	–
Reference	This study	This study	Wang et al., 2020[14]	Huchzermeyer et al., 2023[12]	Nakamura et al., 2019[8]	Fujinami et al., 2019[13]

AD, autosomal dominant; BCVA, best-corrected visual acuity; logMAR, logarithm of the minimum angle of resolution; OD, right eye; OMD, occult macular dystrophy; OS, left eye.
[a]Age: 52.27 ± 9.70 (range 26–71); there is no information for age at onset.

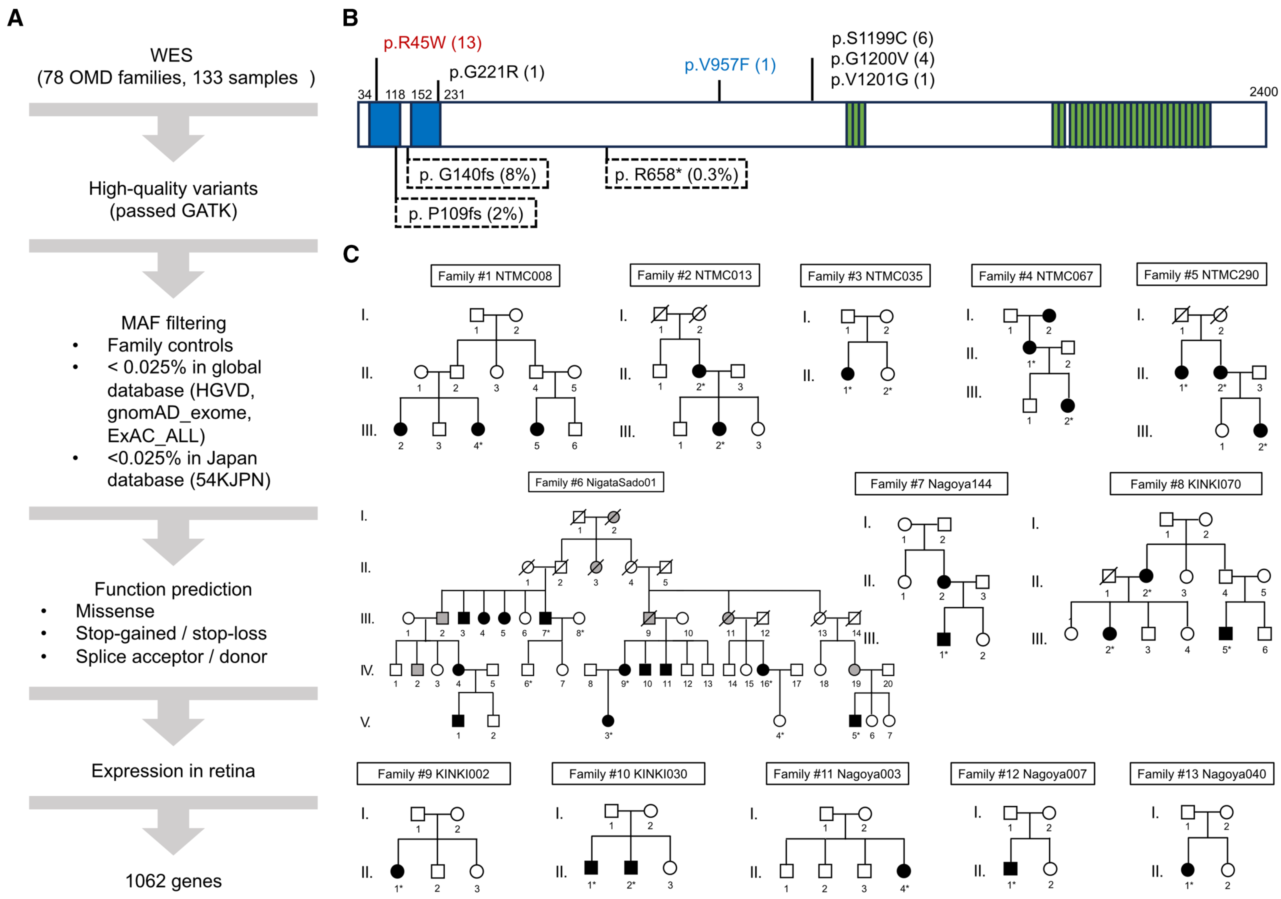

Figure 1. ***RP1L1* R45W mutation identified in 13 OMD families**
(A) Workflow of whole-exome sequencing (WES) analysis conducted in 78 occult macular dystrophy (OMD) families. GATK, Genome Analysis Toolkit; MAF, minor allele frequency.
(B) Protein-level depiction of 6 candidate *RP1L1* (NM_178857.6) variants identified with the family count, including the most frequent variant p.Arg45Trp (R45W) in red and a novel variant p.Val957Phe (V957F) in light blue, along with previously reported variants in black. The top three common truncating *RP1L1* variants from the 54KJPN database are also indicated by the dotted box. The RP1L1 protein comprises two doublecortin domains (blue) and 28 repeats of 16 amino acids (green).
(C) Pedigrees of the 13 OMD families with heterozygous *RP1L1* R45W showing affected individuals (solid squares/circles), unaffected family members (white icons), individuals with reduced visual acuity without diagnosis (gray icons), and deceased individuals (slash symbol). The proband of each pedigree is marked by an asterisk. The generation numbers are shown on the left.

indicating an earlier onset than in general OMD patients from our database ($n = 165$) (Welch's t test, $p = 0.036$). We graded the SD-OCT changes in the photoreceptor layers of these 22 patients and staged 17 (77.27%) as IIb, 4 (18.18%) as IIIa, and 1 (4.55%) as IIIb. All patients had extinguished IZs and blurred or disrupted EZs. Photoreceptor structures of the fovea and parafovea usually deteriorate gradually over the course of OMD. However, the severity of the clinical phenotype (assigned values of SD-OCT stages) of R45W-OMD did not correlate significantly with disease duration (Spearman correlation coefficient = 0.25, $p = 0.27$). The clinical stage, however, was linked to age at disease onset, with earlier onset correlating with more severe symptoms (Spearman correlation coefficient = −0.447, $p = 0.037$) (Figure S1). This suggests that although early onset is associated with more pronounced symptoms, disease duration is not clearly related to clinical severity. The tendency of R45W-OMD patients to exhibit an earlier onset and a more severe clinical phenotype underscores the urgent need for early diagnosis and treatment.

R45W mutation disrupts intracellular localization but not expression

To determine the effects of the R45W mutation on protein synthesis and intracellular trafficking, we generated RP1L1 and R45W expression vectors. Owing to its large size and complexity with 28 repeats, we partitioned the *RP1L1* cDNA into two fragments, individually cloned them into a pCMV-HA vector, and then combined the fragments to construct a full-length RP1L1 overexpression plasmid (Figures 2A–2D; Tables S2 and S3). Although R45W did not affect RP1L1 expression in transfected HEK293T and COS-7 cells according to WB (Figures 2E and 2F), immunofluorescence studies of transfected COS-7 cells revealed overexpressed RP1L1 in the nucleus and cytoplasm, whereas R45W was localized in the cytoplasm (Figure 2G). Similarly, in transfected 661W cells,

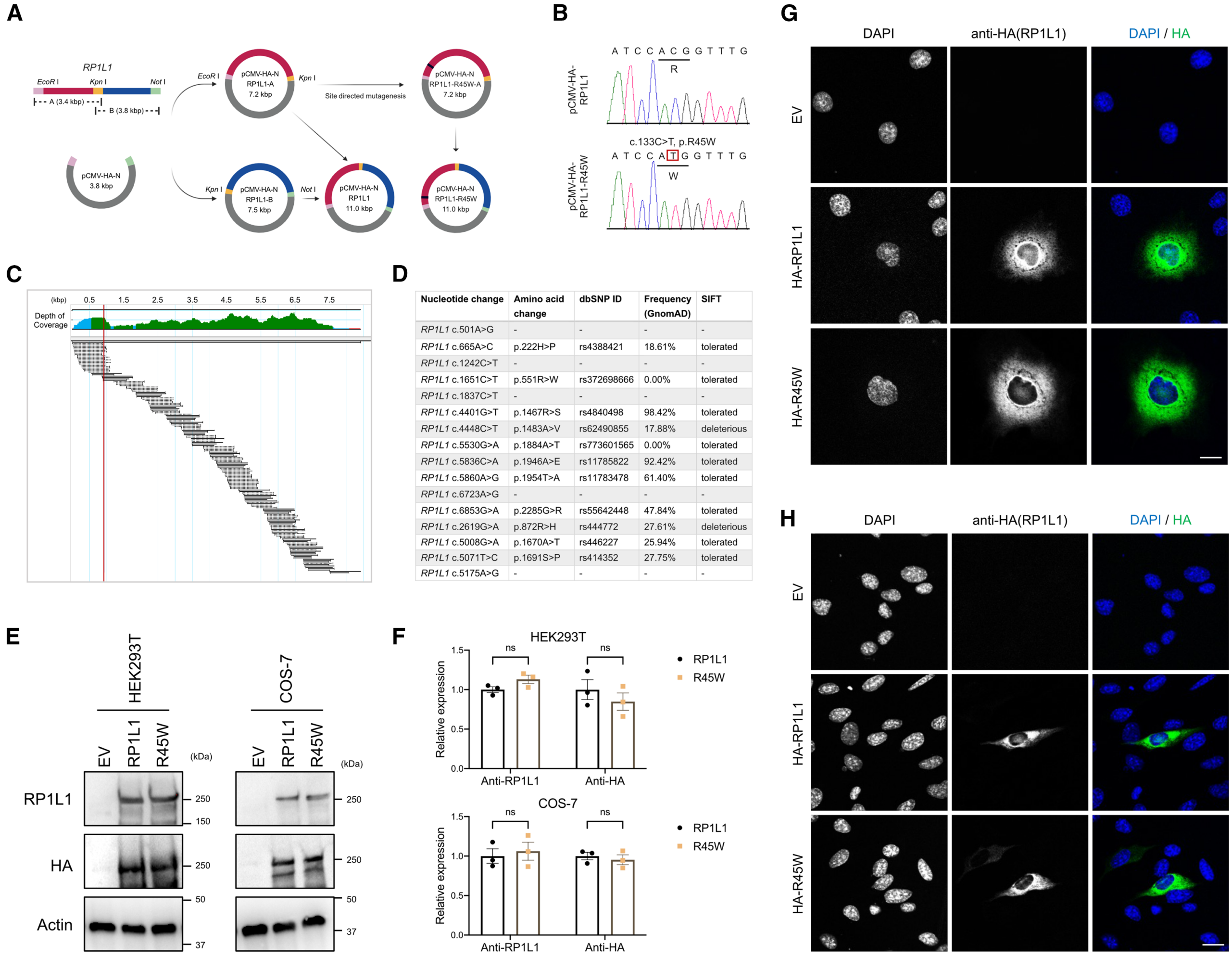

Nucleotide change	Amino acid change	dbSNP ID	Frequency (GnomAD)	SIFT
RP1L1 c.501A>G	-	-	-	-
RP1L1 c.665A>C	p.222H>P	rs4388421	18.61%	tolerated
RP1L1 c.1242C>T	-	-	-	-
RP1L1 c.1651C>T	p.551R>W	rs372698666	0.00%	tolerated
RP1L1 c.1837C>T	-	-	-	-
RP1L1 c.4401G>T	p.1467R>S	rs4840498	98.42%	tolerated
RP1L1 c.4448C>T	p.1483A>V	rs62490855	17.88%	deleterious
RP1L1 c.5530G>A	p.1884A>T	rs773601565	0.00%	tolerated
RP1L1 c.5836C>A	p.1946A>E	rs11785822	92.42%	tolerated
RP1L1 c.5860A>G	p.1954T>A	rs11783478	61.40%	tolerated
RP1L1 c.6723A>G	-	-	-	-
RP1L1 c.6853G>A	p.2285G>R	rs55642448	47.84%	tolerated
RP1L1 c.2619G>A	p.872R>H	rs444772	27.61%	deleterious
RP1L1 c.5008G>A	p.1670A>T	rs446227	25.94%	tolerated
RP1L1 c.5071T>C	p.1691S>P	rs414352	27.75%	tolerated
RP1L1 c.5175A>G	-	-	-	-

Figure 2. *RP1L1* R45W mutation leads to aberrant trafficking *in vitro*

(A) Strategy for generating *RP1L1* and R45W constructs: *RP1L1* was constructed by integrating segments A and B into the pCMV-HA-N vector via specific restriction enzymes. The *RP1L1* R45W mutation was introduced via site-directed mutagenesis, followed by ligation into the *RP1L1-B* vector.

(B) Sanger sequencing confirmed the c.C133T (R45W) mutation in the *RP1L1* R45W construct (marked by the red box).

(C) Sanger sequencing with 65 primers confirmed the integrity of the *RP1L1* sequence, with the R45W mutation indicated (red line).

(D) *In silico* analysis of identified *RP1L1* variants obtained from *RP1L1* and R45W constructs via Sanger sequencing.

(E) The expression of RP1L1 and R45W with an HA tag in HEK293T and COS-7 cells was determined by WB.

(F) Quantification of RP1L1 and R45W expression levels from three independent experiments, presented as the mean ± SEM. Statistical significance was determined by Student's t test.

(G and H) Immunofluorescence localization of RP1L1 and R45W with an N-terminal HA tag in transfected COS-7 (G) and 661W (H) cells. RP1L1 was detected with an anti-HA antibody (green), and nuclei were stained with DAPI (blue). Scale bars: 10 μm. The results are representative of three independent experiments.

the R45W mutation appeared to influence the translocation of RP1L1, with a potential shift from the nucleus to the cytoplasm (Figure 2H).

R45W mutation causes abnormal intracellular localization of RP1

Given that mouse Rp1l1 interacts with Rp1[17] and that pathogenic variants of human *RP1* [MIM: 603937] are well-known causes of autosomal dominant retinitis pigmentosa [RP] [MIM: 268000]),[36,37] we explored the interaction between human RP1L1 and RP1. Because murine Rp1l1 and human RP1L1 share only 46.63% protein sequence homology (Figure S2), docking analysis of the human RP1L1 and RP1 proteins was first performed to predict their interaction and to identify R45 as the potential binding site on RP1L1 (Figures 3A and S3). To validate this interaction and assess the effect of R45W, we created an RP1 expression vector and conducted coimmunoprecipitation experiments (Figures 3A–3D), which demonstrated that RP1L1 binds to RP1 and that R45W does not disrupt this binding (Figure 3D).

Given its effect on intracellular localization, R45W may also influence the relocation of RP1. To investigate this hypothesis, we cotransfected these two proteins and performed immunofluorescence assays. RP1 was expressed

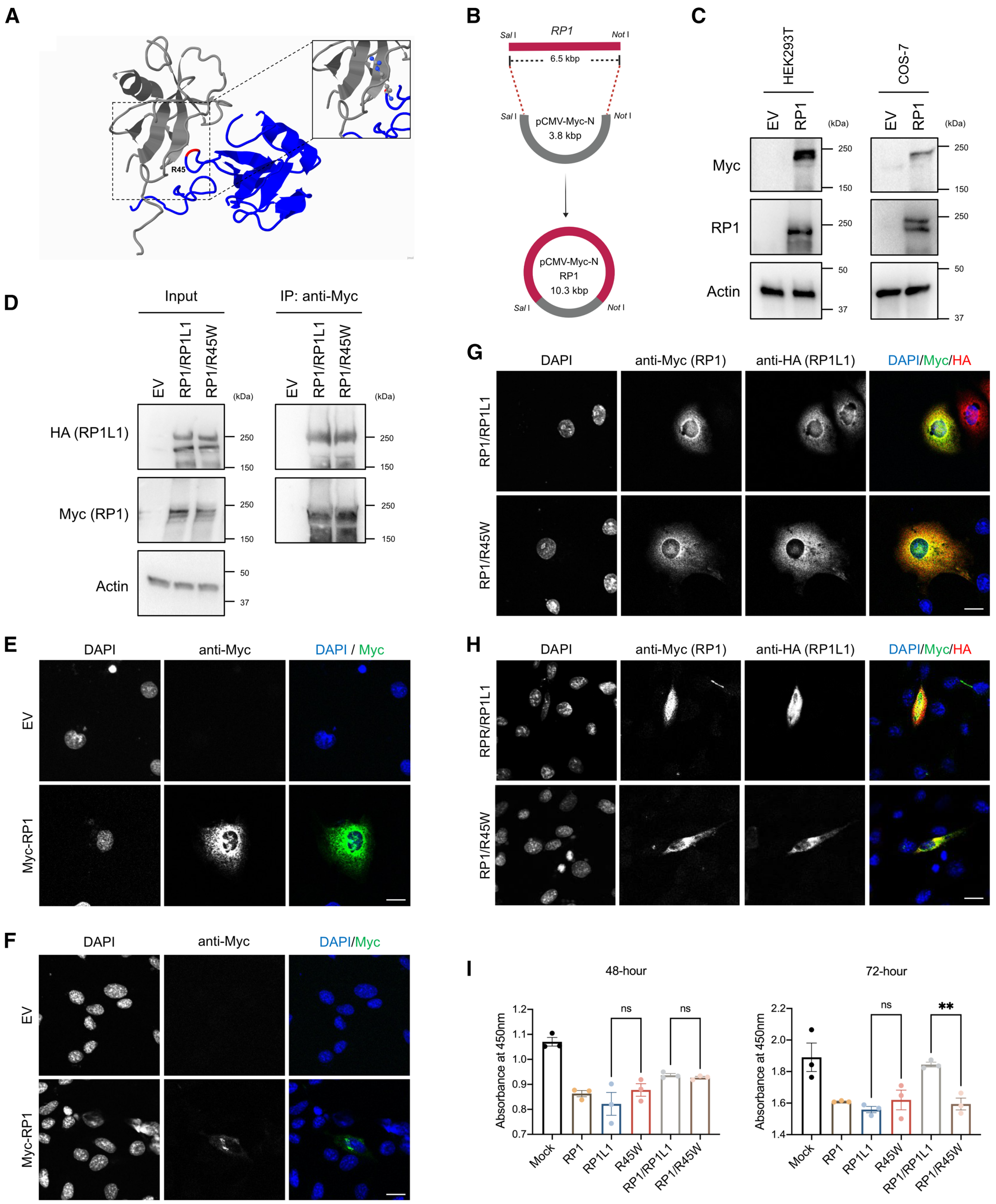

Figure 3. Impact of the R45W mutation on RP1 trafficking *in vitro*

(A) RP1L1 (blue) binds to RP1 (gray) at residue R45W, as illustrated computationally by docking prediction top 1. The Phyre2 server generated RP1L1 and RP1 protein structures, which were visualized with Jmol.

(B) RP1 was incorporated into the pCMV-Myc-N vector via *Sal* I and *Not* I restriction sites to create the expression construct.

(C) Western blotting confirmed RP1 expression with an N-terminal Myc-tag in HEK293T and COS-7 cells.

(D) Coimmunoprecipitation via an anti-Myc antibody in cotransfected HEK293T cells demonstrated that both RP1L1 and R45W interact with RP1, as detected by WB with anti-HA and anti-Myc antibodies. EV, empty vector.

(E–H) Immunofluorescence in COS-7 and 661W cells revealed the subcellular localization of the RP1 and RP1L1 proteins, with RP1 labeled in green (anti-Myc antibody) and RP1L1/R45W labeled in red (anti-HA antibody). Nuclei were stained with DAPI (blue). Scale bars: 10 μm.

(I) Cell viability was significantly inhibited in cells coexpressing R45W and RP1 compared with those coexpressing RP1L1 and RP1 at 72 h posttransfection. Data represent the means ± SEMs from three independent experiments. Statistical significance was determined by one-way ANOVA followed by Tukey's multiple-comparison test (**$p < 0.01$; ns, nonsignificant).

in the nucleus and cytoplasm of transfected COS-7 cells (Figure 3E) and aggregated in the endoplasmic reticulum of transfected 661 cells (Figure 3F). In COS-7 cells cotransfected with RP1L1 and RP1, both proteins localized to the nucleus and cytoplasm, with some difference compared to cells cotransfected with R45W and RP1 (Figure 3G). A similar pattern was observed in cotransfected 661W cells (Figure 3H). To evaluate the impact of R45W on cell survival, we conducted a cell viability assay. Cotransfected COS-7 cells demonstrated that R45W alone did not affect cell survival but reduced viability when it coexpressed with RP1 (Figure 3I).

R45W downregulates lncRNA *MEG3* transcription in iPRCs

RP1L1 is expressed only in the retina[18] and primarily in photoreceptor cells (Human Protein Atlas). Here, iPSCs were generated and differentiated into photoreceptor-like cells to determine the effect of R45W on peripheral blood lymphocytes from patients with R45W-OMD (Figures 4A–4D). iPSCs were transduced with genes encoding two transcription factors, CRX and NEUROD1, via the piggyBac vector with neomycin selection (Figures 4E–4I and S4). *CRX* and *NEUROD1* insertion and expression were confirmed by RT-PCR (Figure 4E) and RT-qPCR (Figure 4F). The integrity of *RP1L1* and *RP1* was confirmed via PCR, which amplified target genes from genomic DNA (Figure S5). We cultured iPRCs until day 28 and conducted RT-qPCR and WB to validate the expressions of photoreceptor-like cell markers that included recoverin, opsin (also known as rhodopsin [RHO]), and arrestin 3 (ARR3), which were not significantly different (Figures 4H and 4I). Congruent with the *in vitro* results, R45W did not affect RP1L1 expression in iPRCs (Figure S6).

To gain further insight into the molecular mechanisms of R45W in OMD, RNA-seq was conducted on iPRCs. Genes meeting the criteria of FC >2 and an FDR <0.05 in comparison with isogenic controls were characterized as upregulated DEGs. In contrast, genes with FC <−2 and FDR <0.05 were considered downregulated DEGs. A total of 1,242 DEGs were identified, including 1,044 upregulated and 198 downregulated DEGs (Figure 5A). Notably, *MEG3*, a lncRNA, was the most strongly (14.5-fold) downregulated, with the lowest FDR ($\log_{10}(\mathrm{FDR}) = -160$) (Figures 5A and 5B), suggesting that it may play an important role in R45W-induced OMD.

GO and Reactome enrichment analyses

To reveal specific affected cellular pathways, GO enrichment and Reactome pathway analyses were performed on up- and downregulated DEGs, respectively (Figures 5C–5F). With respect to biological process, cellular component, and molecular function, the upregulated genes were primarily responsible for extracellular matrix (ECM) organization, the ECM, and the ECM structural constituent, respectively (Figures 5C and 5E). Reactome pathway analysis revealed that the upregulated DEGs were also involved in ECM organization and the downregulation of pathways related to keratinization and the cornified envelope, similar to the results of the GO analyses (Figures 5D and 5F). The interphotoreceptor matrix (IPM) plays a significant etiologic role in retinal degenerative disorders.[38] These results suggest that the ECM may play a crucial role in R45W-induced OMD.

Identification of the PPI network and hub genes

The 1,242 DEGs were uploaded into STRING to construct a PPI network, after which the generated files were imported into the Cytoscape software tool for visualization (Figure 6A). Using the cytoHubba plugin, which is based on all 12 algorithms, the 11 most frequent genes (*EGF*, *MMP2*, *PDGFRB*, *KDR*, *BCL2*, *EGFR*, *PDGFRA*, *CCL2*, *CDKN2A*, *LOX*, and *DCN*) were identified as hub genes (Figure 6B). The MCODE plugin was subsequently used to recognize key clusters, and a significant module consisting of 10 nodes and 41 edges was identified (Figure 6C). Genes involved in the module were *EGF*, *EGFR*, *SOX9*, *CDKN2A*, *LOX*, *PDGFRA*, *MMP2*, *CCL2*, *KDR*, and *BCL2*. A gene network was further constructed via GeneMANIA (Figure 6D). Functional annotation associated most of the hub genes with PI3K signaling, whereas others were associated with ECM organization, the regulation of protein kinase B signaling, the ERK1 and ERK2 cascades, the regulation of the intrinsic apoptosis signaling pathway, calcium ion import, and the positive regulation of apoptosis.

Downregulated PI3K/Akt pathway in R45W-iPRCs

MEG3 regulates ECM production,[39,40] and ECM modulates the PI3K/Akt pathway, influencing cell proliferation, differentiation, and migration.[41] The Akt pathway is constitutively active in cone photoreceptors,[42] and its downregulation or inactivation selectively induces cone death in diabetic retinopathy.[43,44] To determine whether the PI3K/Akt pathway is affected in R45W-iPRCs, RT-qPCR was performed to verify hub gene expressions. The FC values obtained from RT-qPCR and RNA-seq correlated highly (Pearson's correlation coefficient of 0.84, $p = 0.0006$), and the direction of the FC agreed between the two methods for all hub genes (Figures 7A and 7B). We then evaluated Akt expression and phosphorylation at both Ser473 and Thr308, which are required for the full activity of Akt, in R45W-iPRCs via WB (Figures 7C and 7D). Akt expression and Akt phosphorylation at Ser473 were markedly reduced in R45W-iPRCs. With respect to Akt phosphorylation at Thr308, the intensity of the WB band was reduced but not significantly. Taken together, the PI3K/Akt pathway was downregulated in R45W-iPRCs.

Discussion

WES of 133 samples from 78 OMD families revealed the R45W mutation in 13 pedigrees, representing the most

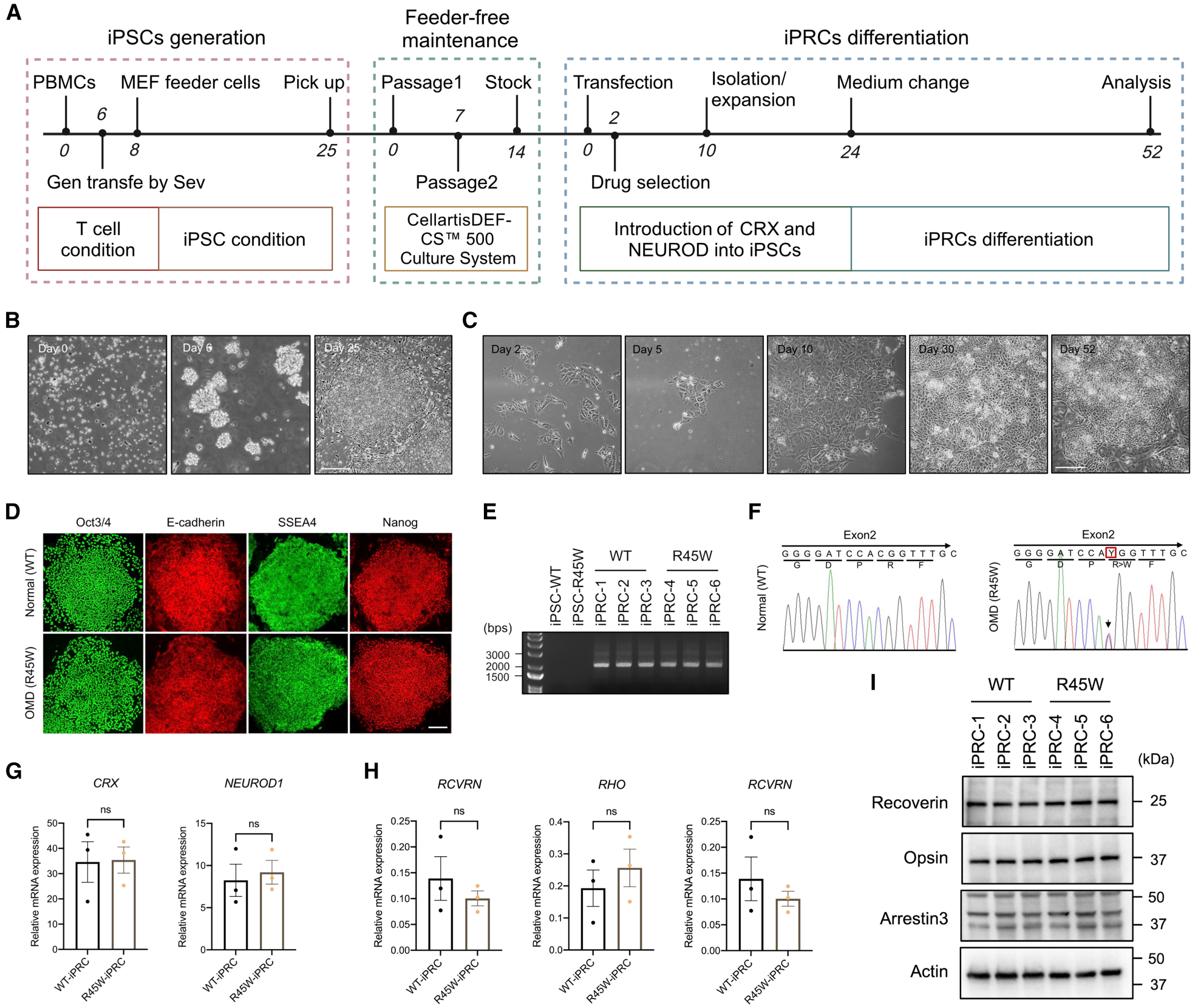

Figure 4. Induced pluripotent stem cell generation and induced photoreceptor-like cell differentiation

(A) Illustration of induced pluripotent stem cell (iPSC) generation and induced photoreceptor-like cell (iPRC) differentiation via CRX and NEUROD1 introduction via a polycistronic *piggyBac* vector.

(B and C) Bright-field images displaying iPSC colonies (B) and iPRCs (C). Scale bar: 200 μm.

(D) Characterization of iPSCs, including iPSC-R45W colonies derived from OMD patient lymphocytes and controls (iPSC-wild-type) from the same family. No significant differences were detected during induction or cultivation. Scale bar: 200 μm.

(E–I) Characterization of iPRCs.

(E) Gel electrophoresis of CRX-NEUROD1 RT-PCR products from iPRCs.

(F) Sanger sequencing chromatogram highlighting the heterozygous c.C133T (p.R45W) mutation in iPRCs (marked by the red box).

(G–I) RT-qPCR (G and H) and WB (I) confirmed the expression of CRX, NEUROD1, and photoreceptor markers, including photoreceptor precursor markers (*PCVRN*, recoverin), rod cell markers (*RHO*, opsin), and cone cell markers (*ARR3*), in iPRCs.

mRNA levels were measured in triplicate relative to the level of GAPDH. Data are presented as the means ± SEMs. ns, nonsignificant difference, as determined by Student's t test.

prevalent (16.67%) pathogenic variant. To our knowledge, the causative R45W mutation was identified for the first time through WES followed by family inheritance-based model filtering in two pedigrees. Patients carrying R45W exhibited earlier disease onset and a more severe clinical phenotype, emphasizing the critical need for early diagnosis and targeted interventions. We observed abnormal intracellular localization of R45W and RP1 without altered expressions or *in vitro* interaction. RNA-seq revealed downregulation of the lncRNA *MEG3* in R45W-iPRCs. Enrichment and PPI network analyses linked R45W to disruptions of ECM organization and PI3K/Akt signaling, which was further confirmed by the observed downregulation of the PI3K/Akt pathway in iPRCs. These findings offer insights into the molecular pathogenesis of R45W-induced OMD, suggest the diagnostic utility of genetic testing for the R45W mutation, and identify potential therapeutic targets.

Since the *RP1L1* gene was identified as the causative gene of OMD,[4] more than 80 *RP1L1* variants have been

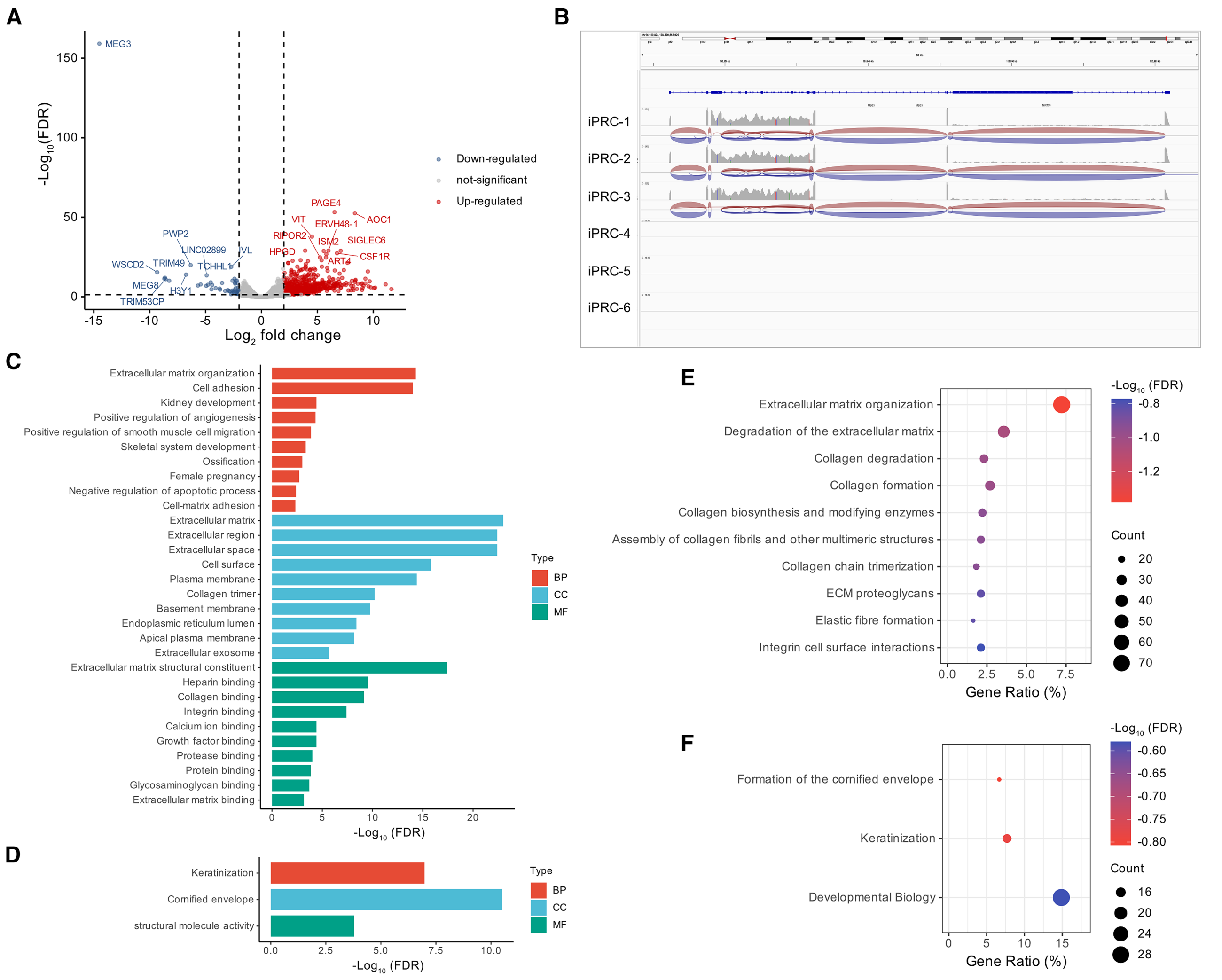

Figure 5. Gene Ontology and pathway enrichment analysis of the differentially expressed genes between R45W-iPRCs and controls

(A) Volcano plot of differentially expressed genes (DEGs) between R45W-iPRCs and isogenic controls. Red dots represent upregulated DEGs ($n = 1{,}044$), blue dots represent downregulated DEGs ($n = 198$), and gray dots represent genes whose expression did not significantly differ between the two groups (FDR <0.05, $|\log_2 FC| > 1$, $n = 3$).
(B) Representative Integrative Genomics Viewer tracks for lncRNA *MEG3* illustrating RNA-seq expression in iPRCs.
(C–F) Gene Ontology and Reactome pathway enrichment analysis ($p < 0.05$) of upregulated (C and E) and downregulated DEGs (D and F) in iPRCs. BP, biological process; CC, cellular component; FC, fold change; MF, molecular function.

associated with OMD in different countries.[8,10,12–14] In this study, we identified 25 unique *RP1L1* variants from the entire candidate gene list obtained through WES, with the MAF of <0.1% in global databases. After applying more stringent MAF filtration by combining the 54KJPN and global databases, 15 *RP1L1* variants were excluded. Functional prediction excluded another 4 *RP1L1* variants, leaving R45W, p.Gly221Arg, p.Val957Phe (novel), p.Ser1199Cys, p.Gly1200Val, and p.Val1201Gly as possible pathogenic mutations. Notably, the common *RP1L1* (NM_178857.6) truncating variants c.416dupC (p.Gly140fs, MAF = 8%), c.324_325insT (p.Pro109fs, MAF = 2%), and c.1972C>T (p.Arg658*, MAF = 0.3%) suggest that knockout of RP1L1 (amino acids 109–2,400, 95.5% of RP1L1) does not induce OMD. R45W, located in the first doublecortin domain, is the only identified mutation unaffected by these frequent truncating variants. Combined with the observation that R45W did not affect RP1L1 expression *in vitro*, these findings suggest that the pathogenesis of RP1L1-induced OMD is related to the function of the doublecortin domain rather than the quantity of RP1L1. Additionally, when a recessive model for WES analysis with an MAF <1% was used, no possible *RP1L1* candidates were identified. Given the complexity of *RP1L1*, with its large size and repetitive region, long sequencing of small sample sizes yielded discrepancies with WES results, suggesting the presence of more common *RP1L1* variants. These observations underscore the need for larger-scale long sequencing studies to comprehensively investigate the full spectrum of RP1L1 mutations, including structure variation, big deletion/insertion, and their contribution to disease pathogenesis.

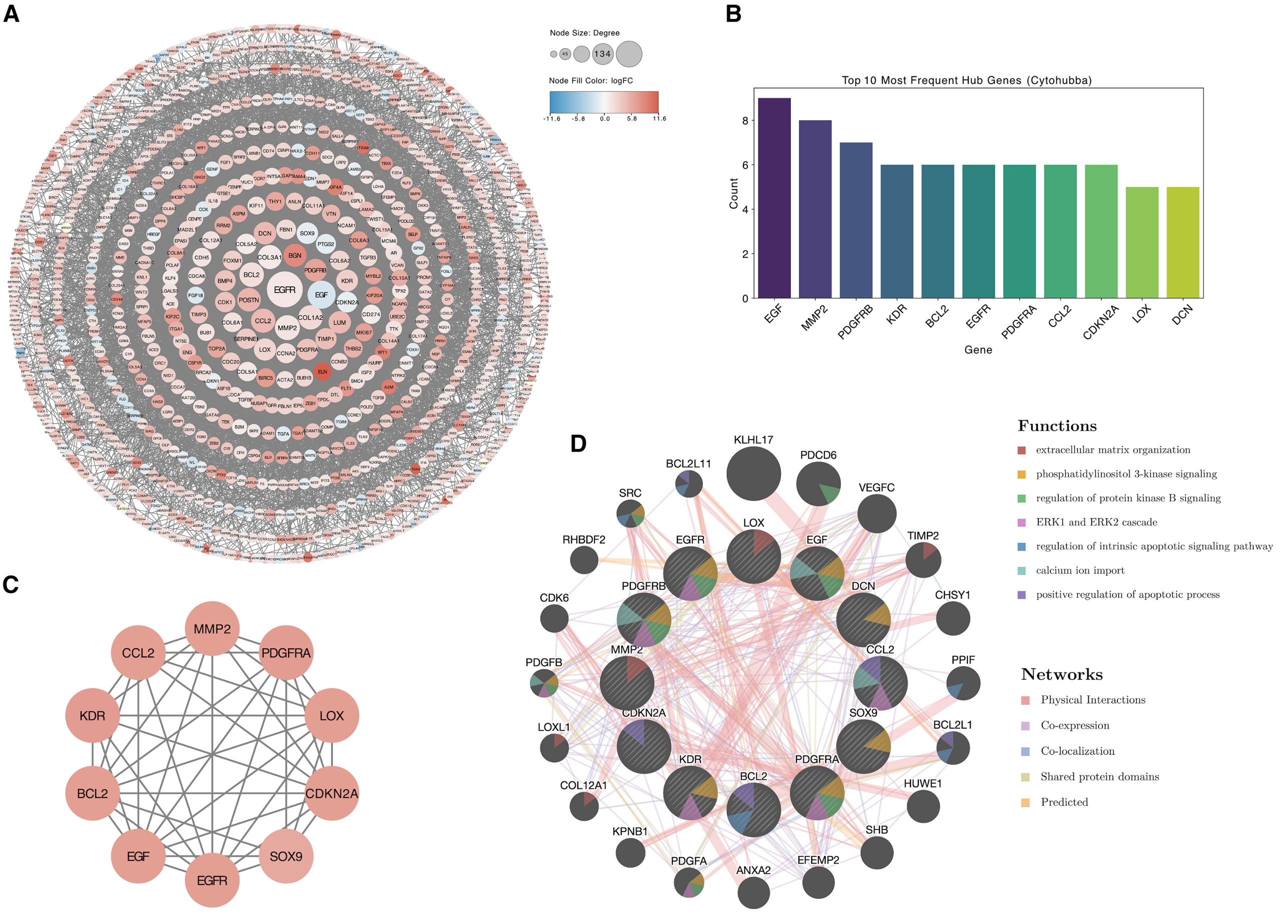

Figure 6. Protein-protein interaction network analysis and identification of hub genes

(A) Protein-protein interaction (PPI) network for common DEGs from iPRCs. Red nodes represent upregulated DEGs, and blue nodes represent downregulated DEGs. The size of the node corresponds to its degree in the network.
(B) The 10 most frequent hub genes were identified according to all 12 algorithms of the CytoHubba plugin in Cytoscape.
(C) A key cluster with 10 genes was further chosen by the MCODE plugin in Cytoscape.
(D) Gene network and functional analyses of the hub genes were performed via GeneMANIA. The inner circle represents the hub genes, and the outer circle represents the corresponding reciprocal genes. The colors of the nodes represent gene function annotations. The colors of the edges represent interactions based on physical interactions, coexpression, colocalization, shared protein domains, or predicted interactions.

RP is the most prevalent IRD and is characterized by the progressive degeneration of both cone and rod photoreceptors.[45] Although mutated genes are usually expressed only by rods, cones die subsequent to rod loss.[46] Because high-acuity vision depends primarily on cones, secondary cone death reduces the quality of life of RP patients.[47] However, the molecular pathogenesis of RP is not fully understood, and no cure or specific treatments are available. Our study revealed that R45W binds to RP1, which is encoded by a causative gene of RP,[36,37] and induces abnormal intracellular positioning of the RP1 in cotransfected COS-7 and 661W cells. Additionally, >10 homozygous or compound heterozygous *RP1L1* variants are associated with autosomal-recessive[48] but not autosomal-dominant[18] RP. These findings suggest that a single-allele *RP1L1* mutation induces OMD, whereas two-allele *RP1L1* mutations induce RP. Specifically, single-allele *RP1L1* mutation leads to cone degeneration, whereas two-allele *RP1L1* mutations cause both cone and rod degeneration, potentially linking the pathogenesis of RP and OMD.

RP1L1 and RP1 are axoneme-associated proteins localized to the outer segment of photoreceptor cells *in vivo*,[17,18] rather than the nucleus. Immunofluorescence studies in transfected COS-7 and 661W cells, which lack the characteristic features of photoreceptor cells, such as the outer segment, revealed differences in the intracellular localization of overexpressed RP1 and RP1L1, including nuclear localization. Overexpression of the R45W mutant protein partially altered this localization, suggesting a potential link between mislocalization and disease mechanisms. Due to the inherent limitations of heterologous systems and the potential artifacts introduced by protein overexpression *in vitro*,[49] these findings should be interpreted with caution. Currently, photoreceptor-like cells differentiated from human iPSCs have emerged as a scalable model for studying retinal diseases.[10–18] Despite these

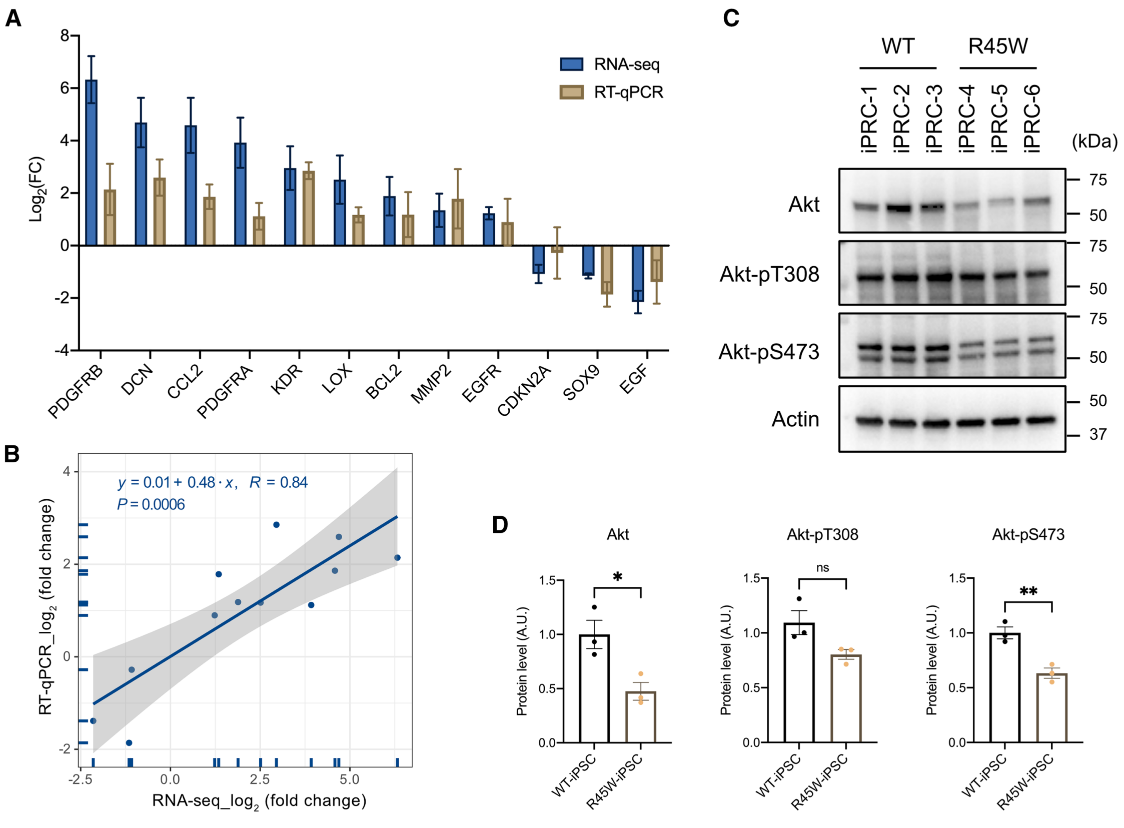

Figure 7. PI3K/Akt signaling is downregulated in R45W-iPRCs compared with controls
(A) RT-qPCR validation of the RNA-seq results for the 12 hub genes. *GAPDH* was used as an internal reference control gene.
(B) RT-qPCR and RNA-seq FC values were highly correlated according to the Pearson correlation.
(C) *RP1L1* R45W reduced Akt expression and phosphorylation at Thr308 and Ser473 in iPRCs, as determined by WB.
(D) Quantification of Akt expression and phosphorylation. All data are presented as means ± SEMs. ns, nonsignificant difference; *$p < 0.05$; **$p < 0.01$ by Student's t test.

advancements, generating robust and functional outer segment structures and purifying large quantities of differentiated photoreceptor cells expressing RHO or cone opsins is still technically difficult. Validation of these findings in models that more accurately replicate photoreceptor architecture, preferably *in vivo*, will be crucial. However, developing and applying such advanced models remain a significant challenge.

lncRNAs, defined as untranslated transcripts longer than 200 nt, are widely expressed and affect various physiological processes, and they play key roles in the nervous system and its associated pathologies.[50–53] lncRNAs are also crucial in the pathogenesis of retinopathies.[54] *MEG3*, a significant lncRNA involved in the PI3K/Akt pathway,[55] has been associated with Alzheimer disease,[56] epilepsy,[57] diabetic retinopathy,[58] and light-induced retinal degeneration.[59] Here, we found strongly downregulated *MEG3* in OMD patient-derived iPRCs, further supporting the role of *MEG3* in neuronal function. lncRNAs with key pathogenic roles could be therapeutic targets because of their high tissue specificity and ability to regulate specific facets of cellular networks.[60] Given the significantly downregulated PI3K/Akt pathway in R45W-iPRCs observed in this study and the downregulation of Akt in RP retinas,[61] the downregulation of *MEG3* suggests potential therapeutic approaches to OMD or even broader photoreceptor and nervous system degenerative diseases. Additionally, FANTOM6[62] enrichment analysis of DEGs from R45W-iPRCs revealed associations between R45W and lncRNAs (Figure S7), suggesting that not only *MEG3* but also other lncRNAs are involved in photoreceptor degeneration.

The retinal ECM is divided into two separate entities, the IPM and the ECM. The ECM encompasses the extracellular areas outside the IPM.[63] The IPM is a highly organized structure surrounding cone and rod photoreceptors that plays a significant role in retinal degenerative disorders[38] and comprises two major components (IMPG1 and IMPG2).[63] Mutations of *IMPG1* and *IMPG2* cause vitelliform macular dystrophies and autosomal-recessive RP, respectively.[38] In both *Impg2*$^{Q244Ter/Q244Ter}$ and *Impg2*$^{T807Ter/T807Ter}$ mice, OCT revealed changes in the outer retinal layers, with the EZs and IZs losing their linear and continuous appearance.[64] The extinguished interdigitation and disrupted ellipsoid zones of OMD patients, along with the dysregulated ECM pathway in R45W-iPRCs, underscore the importance of the ECM in OMD and similar diseases such as IMPG2-induced RP.

The PI3K/Akt pathway plays an important role in regulating cell proliferation, the cell cycle, and apoptosis.[65] In a murine retinal degeneration model, this pathway is inactivated during photoreceptor apoptosis.[61] In diabetic retinopathy, the leading cause of blindness in developed countries, cones die primarily through the downregulation or inactivation of the PI3K/Akt pathway.[44,66] Knockout of the p85α regulatory subunit of PI3K causes cone degeneration.[67] Akt, which is activated by growth factors in a PI3K-dependent manner,[68] is downregulated during photoreceptor apoptosis in RP retinas.[61] Moreover, dysfunctional Akt signaling is also linked to various neurodegenerative disorders that include Alzheimer disease.[68,69] Conversely, activation of the PI3K/Akt pathway can prevent the death of photoreceptors stimulated by bright light[44] and promote neuronal survival.[70] In this study, we observed decreased PI3K/Akt activity in R45W-iPRCs. Collectively, these findings underscore the importance of maintaining PI3K/Akt pathway activity for photoreceptor survival. Understanding the dysregulation of this pathway in OMD and identifying new regulators could inform the development of therapeutic strategies to protect dying photoreceptor cells.

Data and code availability

All phenotype, genotype, and WES data in this study were collected from the NISO database (https://niso.kankakuki.go.jp/opkarte/login.jsp), governed by the National Hospital Organization Tokyo Medical Center, Japan. These data can be accessed upon reasonable request. The RNA-seq data are also available from the corresponding author upon reasonable request.

Acknowledgments

We acknowledge and are grateful to all the patients and families who participated in this study and the contributions from the Japan Eye Genetics Consortium. This work was founded, in part, by AMED grants 22ek0109493h0003, 23ek0109617h0002, and 24ek0109617h0003 (Japan) and the JSPS Grant-in-Aid 23K15923 (Japan).

Author contributions

T.I. conceived the research, Y.P. and D.I. conducted the experiments for this article, and Y.P. and T.I. acquired and interpreted the data and wrote the manuscript. K.T. helped with phenotyping the patient and sample collections, K.Y. and Y.P. analyzed the WES and RNA-seq data, and K.T. and K.Y. helped with manuscript writing and revision. All authors have read and approved the final version.

Declaration of interests

The authors declare no competing interests.

Supplemental information

Supplemental information can be found online at https://doi.org/10.1016/j.xhgg.2025.100461.

Web resources

DAVID, https://david.ncifcrf.gov/tools.jsp
GeneMANIA, https://genemania.org
Hiplot Pro, https://hiplot.cn
Human Protein Atlas, https://www.proteinatlas.org
NCBI, https://www.ncbi.nlm.nih.gov/nuccore
OMIM, https://www.omim.org/
REVIGO, http://revigo.irb.hr/
STRING, https://string-db.org

Received: November 8, 2024
Accepted: May 27, 2025

References

1. Berger, W., Kloeckener-Gruissem, B., and Neidhardt, J. (2010). The molecular basis of human retinal and vitreoretinal diseases. Prog. Retin. Eye Res. *29*, 335–375. https://doi.org/10.1016/j.preteyeres.2010.03.004.
2. Rahman, N., Georgiou, M., Khan, K.N., and Michaelides, M. (2020). Macular dystrophies: clinical and imaging features, molecular genetics and therapeutic options. Br. J. Ophthalmol. *104*, 451–460. https://doi.org/10.1136/bjophthalmol-2019-315086.
3. Fujinami, K., Kameya, S., Kikuchi, S., Ueno, S., Kondo, M., Hayashi, T., Shinoda, K., Machida, S., Kuniyoshi, K., Kawamura, Y., et al. (2016). Novel *RP1L1* Variants and Genotype–Photoreceptor Microstructural Phenotype Associations in Cohort of Japanese Patients With Occult Macular Dystrophy. Investig. Ophthalmol. Vis. Sci. *57*, 4837–4846. https://doi.org/10.1167/iovs.16-19670.
4. Akahori, M., Tsunoda, K., Miyake, Y., Fukuda, Y., Ishiura, H., Tsuji, S., Usui, T., Hatase, T., Nakamura, M., Ohde, H., et al. (2010). Dominant Mutations in RP1L1 Are Responsible for Occult Macular Dystrophy. Am. J. Hum. Genet. *87*, 424–429. https://doi.org/10.1016/j.ajhg.2010.08.009.
5. Miyake, Y., Ichikawa, K., Shiose, Y., and Kawase, Y. (1989). Hereditary Macular Dystrophy without Visible Fundus Abnormality. Am. J. Ophthalmol. *108*, 292–299. https://doi.org/10.1016/0002-9394(89)90120-7.
6. Tsunoda, K., and Hanazono, G. (2022). Detailed analyses of microstructure of photoreceptor layer at different severities of occult macular dystrophy by ultrahigh-resolution SD-OCT. Am. J. Ophthalmol. Case Rep. *26*, 101490. https://doi.org/10.1016/j.ajoc.2022.101490.
7. Tsunoda, K., Usui, T., Hatase, T., Yamai, S., Fujinami, K., Hanazono, G., Shinoda, K., Ohde, H., Akahori, M., Iwata, T., and Miyake, Y. (2012). CLINICAL CHARACTERISTICS OF OCCULT MACULAR DYSTROPHY IN FAMILY WITH MUTATION OF RP1L1 GENE. Retina (Phila., Pa.) *32*, 1135–1147. https://doi.org/10.1097/IAE.0b013e318232c32e.
8. Nakamura, N., Tsunoda, K., Mizuno, Y., Usui, T., Hatase, T., Ueno, S., Kuniyoshi, K., Hayashi, T., Katagiri, S., Kondo, M., et al. (2019). Clinical Stages of Occult Macular Dystrophy Based on Optical Coherence Tomographic Findings. Investig. Ophthalmol. Vis. Sci. *60*, 4691–4700. https://doi.org/10.1167/iovs.19-27486.
9. Miyake, Y., and Tsunoda, K. (2015). Occult macular dystrophy. Jpn. J. Ophthalmol. *59*, 71–80. https://doi.org/10.1007/s10384-015-0371-7.

10. Zobor, D., Zobor, G., Hipp, S., Baumann, B., Weisschuh, N., Biskup, S., Sliesoraityte, I., Zrenner, E., and Kohl, S. (2018). Phenotype Variations Caused by Mutations in the *RP1L1* Gene in a Large Mainly German Cohort. Investig. Ophthalmol. Vis. Sci. *59*, 3041–3052. https://doi.org/10.1167/iovs.18-24033.
11. Liu, J., Hayden, M.R., and Yang, Y. (2024). Research progress of RP1L1 gene in disease. Gene (Amst.) *912*, 148367. https://doi.org/10.1016/j.gene.2024.148367.
12. Huchzermeyer, C., Fars, J., Kremers, J., Kühlewein, L., Kempf, M., Ott, S., Stingl, K., and Stingl, K. (2023). Photoreceptor-Specific Temporal Contrast Sensitivities in RP1L1-Associated Occult Macular Dystrophy. Investig. Ophthalmol. Vis. Sci. *64*, 33. https://doi.org/10.1167/iovs.64.7.33.
13. Fujinami, K., Yang, L., Joo, K., Tsunoda, K., Kameya, S., Hanazono, G., Fujinami-Yokokawa, Y., Arno, G., Kondo, M., Nakamura, N., et al. (2019). Clinical and Genetic Characteristics of East Asian Patients with Occult Macular Dystrophy (Miyake Disease). Ophthalmology *126*, 1432–1444. https://doi.org/10.1016/j.ophtha.2019.04.032.
14. Wang, D.-D., Gao, F.-J., Li, J.-K., Chen, F., Hu, F.-Y., Xu, G.-Z., Zhang, J.-G., Sun, H.-X., Zhang, S.-H., Xu, P., et al. (2020). Clinical and Genetic Characteristics of Chinese Patients with Occult Macular Dystrophy. Investig. Ophthalmol. Vis. Sci. *61*, 10. https://doi.org/10.1167/iovs.61.3.10.
15. Liu, X., Long, Y., Wang, Y., Liu, B., Ren, J., Wang, G., Wang, M., Meng, X., and Liu, Y. (2024). Varied clinical presentations of RP1L1 variants in Chinese patients: a study of occult macular dystrophy and vitelliform macular dystrophy. BMC Ophthalmol. *24*, 327. https://doi.org/10.1186/s12886-024-03591-7.
16. International HapMap Consortium (2005). A haplotype map of the human genome. Nature (London) *437*, 1299–1320. https://doi.org/10.1038/nature04226.
17. Yamashita, T., Liu, J., Gao, J., LeNoue, S., Wang, C., Kaminoh, J., Bowne, S.J., Sullivan, L.S., Daiger, S.P., Zhang, K., et al. (2009). Essential and Synergistic Roles of RP1 and RP1L1 in Rod Photoreceptor Axoneme and Retinitis Pigmentosa. J. Neurosci. *29*, 9748–9760. https://doi.org/10.1523/JNEUROSCI.5854-08.2009.
18. Bowne, S.J., Daiger, S.P., Malone, K.A., Heckenlively, J.R., Kennan, A., Humphries, P., Hughbanks-Wheaton, D., Birch, D.G., Liu, Q., Pierce, E.A., et al. (2003). Characterization of RP1L1, a highly polymorphic paralog of the retinitis pigmentosa 1 (RP1) gene. Mol. Vis. *9*, 129–137.
19. Makałowski, W., Zhang, J., and Boguski, M.S. (1996). Comparative analysis of 1196 orthologous mouse and human full-length mRNA and protein sequences. Genome Res. *6*, 846–857. https://doi.org/10.1101/gr.6.9.846.
20. Otsuka, Y., Imamura, K., Oishi, A., Kondo, T., Suga, M., Yada, Y., Shibukawa, R., Okanishi, Y., Sagara, Y., Tsukita, K., et al. (2022). One-step induction of photoreceptor-like cells from human iPSCs by delivering transcription factors. iScience *25*, 103987. https://doi.org/10.1016/j.isci.2022.103987.
21. Hasegawa, T., Ueda, T., Okamoto, M., and Ogata, N. (2014). Presence of Foveal Bulge in Optical Coherence Tomographic Images in Eyes With Macular Edema Associated With Branch Retinal Vein Occlusion. Am. J. Ophthalmol. *157*, 390–396.e1. https://doi.org/10.1016/j.ajo.2013.10.007.
22. Pan, Y., Suga, A., Kimura, I., Kimura, C., Minegishi, Y., Nakayama, M., Yoshitake, K., Iejima, D., Minematsu, N., Yamamoto, M., et al. (2022). METTL23 mutation alters histone H3R17 methylation in normal-tension glaucoma. J. Clin. Investig. *132*, e153589. https://doi.org/10.1172/JCI153589.
23. Sjöstedt, E., Zhong, W., Fagerberg, L., Karlsson, M., Mitsios, N., Adori, C., Oksvold, P., Edfors, F., Limiszewska, A., Hikmet, F., et al. (2020). An atlas of the protein-coding genes in the human, pig, and mouse brain. Sci. Technol. Humanit. *367*, eaay5947. https://doi.org/10.1126/science.aay5947.
24. Kelley, L.A., Mezulis, S., Yates, C.M., Wass, M.N., and Sternberg, M.J.E. (2015). The Phyre2 web portal for protein modeling, prediction and analysis. Nat. Protoc. *10*, 845–858. https://doi.org/10.1038/nprot.2015.053.
25. Pierce, B.G., Wiehe, K., Hwang, H., Kim, B.-H., Vreven, T., and Weng, Z. (2014). ZDOCK server: interactive docking prediction of protein–protein complexes and symmetric multimers. Bioinformatics *30*, 1771–1773. https://doi.org/10.1093/bioinformatics/btu097.
26. Dobin, A., Davis, C.A., Schlesinger, F., Drenkow, J., Zaleski, C., Jha, S., Batut, P., Chaisson, M., and Gingeras, T.R. (2013). STAR: ultrafast universal RNA-seq aligner. Bioinformatics *29*, 15–21. https://doi.org/10.1093/bioinformatics/bts635.
27. Ewels, P., Magnusson, M., Lundin, S., and Käller, M. (2016). MultiQC: summarize analysis results for multiple tools and samples in a single report. Bioinformatics *32*, 3047–3048. https://doi.org/10.1093/bioinformatics/btw354.
28. Schmieder, R., and Edwards, R. (2011). Quality control and preprocessing of metagenomic datasets. Bioinformatics *27*, 863–864. https://doi.org/10.1093/bioinformatics/btr026.
29. Bolger, A.M., Lohse, M., and Usadel, B. (2014). Trimmomatic: a flexible trimmer for Illumina sequence data. Bioinformatics *30*, 2114–2120. https://doi.org/10.1093/bioinformatics/btu170.
30. Robinson, M.D., McCarthy, D.J., and Smyth, G.K. (2010). edgeR : a Bioconductor package for differential expression analysis of digital gene expression data. Bioinformatics *26*, 139–140. https://doi.org/10.1093/bioinformatics/btp616.
31. Liao, Y., Smyth, G.K., and Shi, W. (2013). The Subread aligner: fast, accurate and scalable read mapping by seed-and-vote. Nucleic Acids Res. *41*, e108. https://doi.org/10.1093/nar/gkt214.
32. Dai, S., Cao, T., Shen, H., Zong, X., Gu, W., Li, H., Wei, L., Huang, H., Yu, Y., Chen, Y., et al. (2023). Landscape of molecular crosstalk between SARS-CoV-2 infection and cardiovascular diseases: emphasis on mitochondrial dysfunction and immune-inflammation. J. Transl. Med. *21*, 915. https://doi.org/10.1186/s12967-023-04787-z.
33. Szklarczyk, D., Kirsch, R., Koutrouli, M., Nastou, K., Mehryary, F., Hachilif, R., Gable, A.L., Fang, T., Doncheva, N.T., Pyysalo, S., et al. (2023). The STRING database in 2023: protein–protein association networks and functional enrichment analyses for any sequenced genome of interest. Nucleic Acids Res. *51*, D638–D646. https://doi.org/10.1093/nar/gkac1000.
34. Rath, S., Sharma, R., Gupta, R., Ast, T., Chan, C., Durham, T. J., Goodman, R.P., Grabarek, Z., Haas, M.E., Hung, W.H.W., et al. (2021). MitoCarta3.0: an updated mitochondrial proteome now with sub-organelle localization and pathway annotations. Nucleic Acids Res. *49*, D1541–D1547. https://doi.org/10.1093/nar/gkaa1011.
35. Nakanishi, A., Ueno, S., Kawano, K., Ito, Y., Kominami, T., Yasuda, S., Kondo, M., Tsunoda, K., Iwata, T., and Terasaki, H. (2015). Pathologic Changes of Cone Photoreceptors in

Eyes With Occult Macular Dystrophy. Investig. Ophthalmol. Vis. Sci. *56*, 7243–7249. https://doi.org/10.1167/iovs.15-16742.
36. Sullivan, L.S., Heckenlively, J.R., Bowne, S.J., Zuo, J., Hide, W. A., Gal, A., Denton, M., Inglehearn, C.F., Blanton, S.H., and Daiger, S.P. (1999). Mutations in a novel retina-specific gene cause autosomal dominant retinitis pigmentosa. Nat. Genet. *22*, 255–259. https://doi.org/10.1038/10314.
37. Pierce, E.A., Quinn, T., Meehan, T., McGee, T.L., Berson, E.L., and Dryja, T.P. (1999). Mutations in a gene encoding a new oxygen-regulated photoreceptor protein cause dominant retinitis pigmentosa. Nat. Genet. *22*, 248–254. https://doi.org/10.1038/10305.
38. Ishikawa, M., Sawada, Y., and Yoshitomi, T. (2015). Structure and function of the interphotoreceptor matrix surrounding retinal photoreceptor cells. Exp. Eye Res. *133*, 3–18. https://doi.org/10.1016/j.exer.2015.02.017.
39. Chen, K., Zhu, H., Zheng, M.-Q., and Dong, Q.-R. (2021). LncRNA MEG3 Inhibits the Degradation of the Extracellular Matrix of Chondrocytes in Osteoarthritis via Targeting miR-93/TGFBR2 Axis. CARTILAGE *13*, 1274S–1284S. https://doi.org/10.1177/1947603519855759.
40. Piccoli, M.-T., Gupta, S.K., Viereck, J., Foinquinos, A., Samolovac, S., Kramer, F.L., Garg, A., Remke, J., Zimmer, K., Batkai, S., and Thum, T. (2017). Inhibition of the Cardiac Fibroblast–Enriched lncRNA *Meg3* Prevents Cardiac Fibrosis and Diastolic Dysfunction. Circ. Res. *121*, 575–583. https://doi.org/10.1161/CIRCRESAHA.117.310624.
41. Hastings, J.F., Skhinas, J.N., Fey, D., Croucher, D.R., and Cox, T.R. (2019). The extracellular matrix as a key regulator of intracellular signalling networks. Br. J. Pharmacol. *176*, 82–92. https://doi.org/10.1111/bph.14195.
42. Johnson, L.E., Van Veen, T., and Ekström, P.A.R. (2005). Differential Akt activation in the photoreceptors of normal and rd1 mice. Cell Tissue Res. *320*, 213–222. https://doi.org/10.1007/s00441-004-1046-8.
43. Rajala, R.V.S. (2010). Phosphoinositide 3-kinase signaling in the vertebrate retina. J. Lipid Res. *51*, 4–22. https://doi.org/10.1194/jlr.R000232.
44. Reiter, C.E.N., Wu, X., Sandirasegarane, L., Nakamura, M., Gilbert, K.A., Singh, R.S.J., Fort, P.E., Antonetti, D.A., and Gardner, T.W. (2006). Diabetes Reduces Basal Retinal Insulin Receptor Signaling. Diabetes (New York, N. Y.) *55*, 1148–1156. https://doi.org/10.2337/diabetes.55.04.06.db05-0744.
45. Hartong, D.T., Berson, E.L., and Dryja, T.P. (2006). Retinitis pigmentosa. Lancet (N. Am. Ed.) *368*, 1795–1809. https://doi.org/10.1016/S0140-6736(06)69740-7.
46. Strettoi, E., and Pignatelli, V. (2000). Modifications of retinal neurons in a mouse model of retinitis pigmentosa. Proc. Natl. Acad. Sci. USA *97*, 11020–11025. https://doi.org/10.1073/pnas.190291097.
47. Lin, B., Xiong, G., and Yang, W. (2018). Ribosomal protein S6 kinase 1 promotes the survival of photoreceptors in retinitis pigmentosa. Cell Death Dis. *9*, 1141. https://doi.org/10.1038/s41419-018-1198-1.
48. Noel, N.C.L., and MacDonald, I.M. (2020). RP1L1 and inherited photoreceptor disease: A review. Surv. Ophthalmol. *65*, 725–739. https://doi.org/10.1016/j.survophthal.2020.04.005.
49. Prelich, G. (2012). Gene overexpression: uses, mechanisms, and interpretation. Genetics (Austin, Tex.) *190*, 841–854. https://doi.org/10.1534/genetics.111.136911.
50. Perry, R.B.-T., Hezroni, H., Goldrich, M.J., and Ulitsky, I. (2018). Regulation of Neuroregeneration by Long Noncoding RNAs. Mol. Cell *72*, 553–567.e5. https://doi.org/10.1016/j.molcel.2018.09.021.
51. He, D., Wang, J., Lu, Y., Deng, Y., Zhao, C., Xu, L., Chen, Y., Hu, Y.-C., Zhou, W., and Lu, Q.R. (2017). lncRNA Functional Networks in Oligodendrocytes Reveal Stage-Specific Myelination Control by an lncOL1/Suz12 Complex in the CNS. Neuron (Camb., Mass.) *93*, 362–378. https://doi.org/10.1016/j.neuron.2016.11.044.
52. Esteller, M. (2011). Non-coding RNAs in human disease. Nat. Rev. Genet. *12*, 861–874. https://doi.org/10.1038/nrg3074.
53. Fatica, A., and Bozzoni, I. (2014). Long non-coding RNAs: new players in cell differentiation and development. Nat. Rev. Genet. *15*, 7–21. https://doi.org/10.1038/nrg3606.
54. Song, J., and Kim, Y.-K. (2021). Targeting non-coding RNAs for the treatment of retinal diseases. Mol. Ther. Nucleic Acids *24*, 284–293. https://doi.org/10.1016/j.omtn.2021.02.031.
55. Royer, M., Pai, B., Menon, R., Bludau, A., Gryksa, K., Perry, R. B.-T., Ulitsky, I., Meister, G., and Neumann, I.D. (2022). Transcriptome and chromatin alterations in social fear indicate association of MEG3 with successful extinction of fear. Mol. Psychiatr. *27*, 4064–4076. https://doi.org/10.1038/s41380-022-01481-2.
56. Balusu, S., Horré, K., Thrupp, N., Craessaerts, K., Snellinx, A., Serneels, L., T'Syen, D., Chrysidou, I., Arranz, A.M., Sierksma, A., et al. (2023). MEG3 activates necroptosis in human neuron xenografts modeling Alzheimer's disease. Sci. Technol. Humanit. *381*, 1176–1182. https://doi.org/10.1126/science.abp9556.
57. Zhang, H., Tao, J., Zhang, S., and Lv, X. (2020). LncRNA MEG3 Reduces Hippocampal Neuron Apoptosis via the PI3K/AKT/mTOR Pathway in a Rat Model of Temporal Lobe Epilepsy. NDT *16*, 2519–2528. https://doi.org/10.2147/NDT.S270614.
58. Qiu, G.-Z., Tian, W., Fu, H.-T., Li, C.-P., and Liu, B. (2016). Long noncoding RNA-MEG3 is involved in diabetes mellitus-related microvascular dysfunction. Biochem. Biophys. Res. Commun. *471*, 135–141. https://doi.org/10.1016/j.bbrc.2016.01.164.
59. Zhu, Y.-X., Yao, J., Liu, C., Hu, H.-T., Li, X.-M., Ge, H.-M., Zhou, Y.-F., Shan, K., Jiang, Q., and Yan, B. (2018). Long non-coding RNA MEG3 silencing protects against light-induced retinal degeneration. Biochem. Biophys. Res. Commun. *496*, 1236–1242. https://doi.org/10.1016/j.bbrc.2018.01.177.
60. Statello, L., Guo, C.-J., Chen, L.-L., and Huarte, M. (2021). Gene regulation by long non-coding RNAs and its biological functions. Nat. Rev. Mol. Cell Biol. *22*, 96–118. https://doi.org/10.1038/s41580-020-00315-9.
61. Jomary, C., Cullen, J., and Jones, S.E. (2006). Inactivation of the Akt Survival Pathway during Photoreceptor Apoptosis in the Retinal Degeneration Mouse. Investig. Ophthalmol. Vis. Sci. *47*, 1620–1629. https://doi.org/10.1167/iovs.05-1176.
62. Yip, C.W., Hon, C.-C., Yasuzawa, K., Sivaraman, D.M., Ramilowski, J.A., Shibayama, Y., Agrawal, S., Prabhu, A.V., Parr, C., Severin, J., et al. (2022). Antisense-oligonucleotide-mediated perturbation of long non-coding RNA reveals functional features in stem cells and across cell types. Cell Rep. *41*, 111893. https://doi.org/10.1016/j.celrep.2022.111893.

63. Al-Ubaidi, M.R., Naash, M.I., and Conley, S.M. (2013). A Perspective on the Role of the Extracellular Matrix in Progressive Retinal Degenerative Disorders. Investig. Ophthalmol. Vis. Sci. *54*, 8119–8124. https://doi.org/10.1167/iovs.13-13536.

64. Williams, B.N., Draper, A., Lang, P.F., Lewis, T.R., Smith, A.L., Mayerl, S.J., Rougié, M., Simon, J.M., Arshavsky, V. Y., Greenwald, S.H., et al. (2024). Heterogeneity in the progression of retinal pathologies in mice harboring patient mimicking Impg2 mutations. Hum. Mol. Genet. *33*, 448–464. https://doi.org/10.1093/hmg/ddad199.

65. Rodon, J., Dienstmann, R., Serra, V., and Tabernero, J. (2013). Development of PI3K inhibitors: lessons learned from early clinical trials. Nat. Rev. Clin. Oncol. *10*, 143–153. https://doi.org/10.1038/nrclinonc.2013.10.

66. Reiter, C.E.N., and Gardner, T.W. (2003). Functions of insulin and insulin receptor signaling in retina: possible implications for diabetic retinopathy. Prog. Retin. Eye Res. *22*, 545–562. https://doi.org/10.1016/S1350-9462(03)00035-1.

67. Ivanovic, I., Anderson, R.E., Le, Y.Z., Fliesler, S.J., Sherry, D. M., and Rajala, R.V.S. (2011). Deletion of the p85alpha regulatory subunit of phosphoinositide 3-kinase in cone photoreceptor cells results in cone photoreceptor degeneration. Investig. Ophthalmol. Vis. Sci. *52*, 3775–3783. https://doi.org/10.1167/iovs.10-7139.

68. Manning, B.D., and Toker, A. (2017). AKT/PKB Signaling: Navigating the Network. Cell (Cambridge, MA, U. S.) *169*, 381–405. https://doi.org/10.1016/j.cell.2017.04.001.

69. Griffin, R.J., Moloney, A., Kelliher, M., Johnston, J.A., Ravid, R., Dockery, P., O'Connor, R., and O'Neill, C. (2005). Activation of Akt/PKB, increased phosphorylation of Akt substrates and loss and altered distribution of Akt and PTEN are features of Alzheimer's disease pathology. J. Neurochem. *93*, 105–117. https://doi.org/10.1111/j.1471-4159.2004.02949.x.

70. Brunet, A., Bonni, A., Zigmond, M.J., Lin, M.Z., Juo, P., Hu, L. S., Anderson, M.J., Arden, K.C., Blenis, J., and Greenberg, M. E. (1999). Akt Promotes Cell Survival by Phosphorylating and Inhibiting a Forkhead Transcription Factor. Cell (Cambridge, MA, U. S.) *96*, 857–868. https://doi.org/10.1016/S0092-8674(00)80595-4.

HGGA, Volume *6*

Supplemental information

Clinical features and molecular mechanisms of *RP1L1* variants causing occult macular dystrophy

Yang Pan, Daisuke Iejima, Kazutoshi Yoshitake, Kazushige Tsunoda, Takeshi Iwata, and on behalf of the Japan Eye Genetics Consortium

Supplemental material

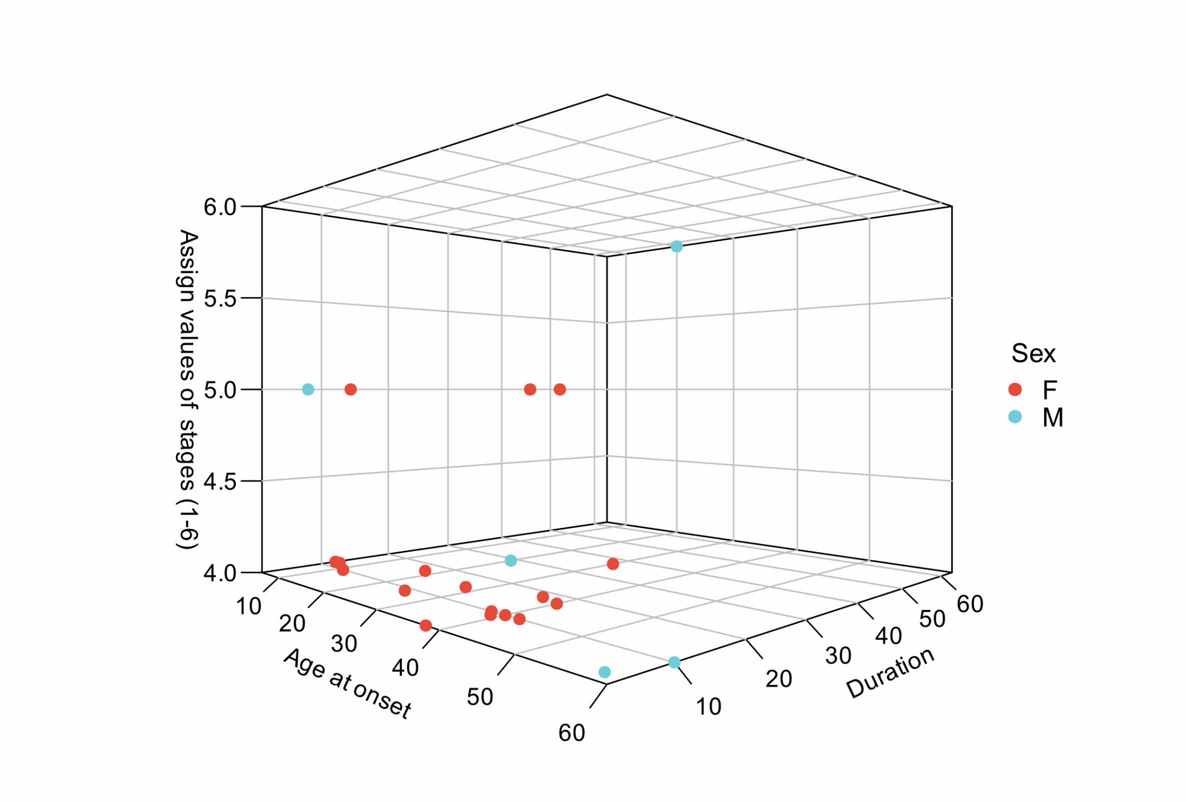

Figure S1. Correlation analysis in a 3D scatter plot

The 3D scatter plot shows the correlation between age at onset and OMD stage, as well as the correlation between duration and OMD stage. The female data points are represented by red dots, whereas the male data points are represented by blue dots.

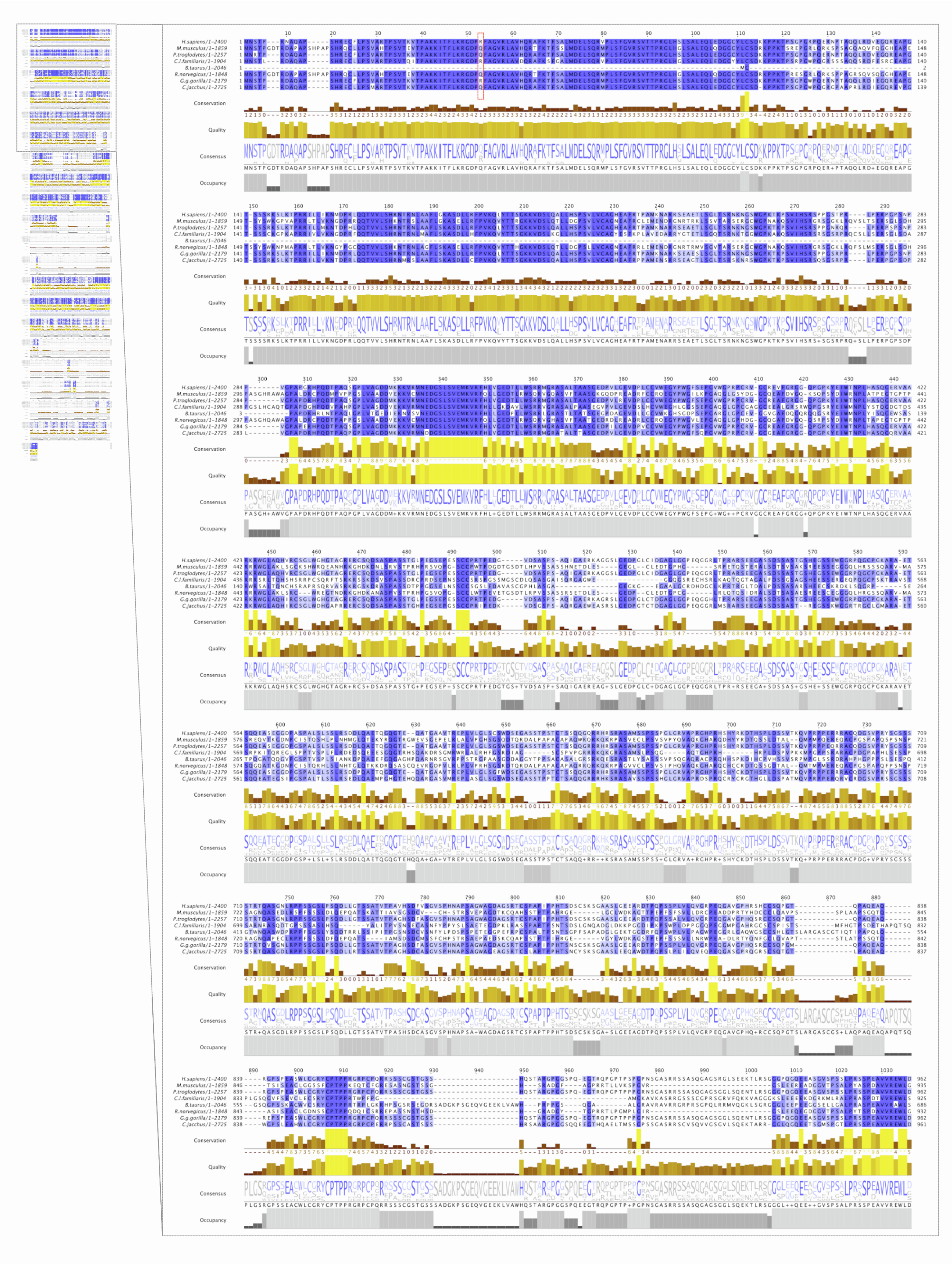

Figure S2. Multiple sequence alignment for RP1L1 in Jalview

The position of the variant is shown below. Histograms show the degree of conservation at each residue. The human RP1L1 R45 is indicated by the red box.

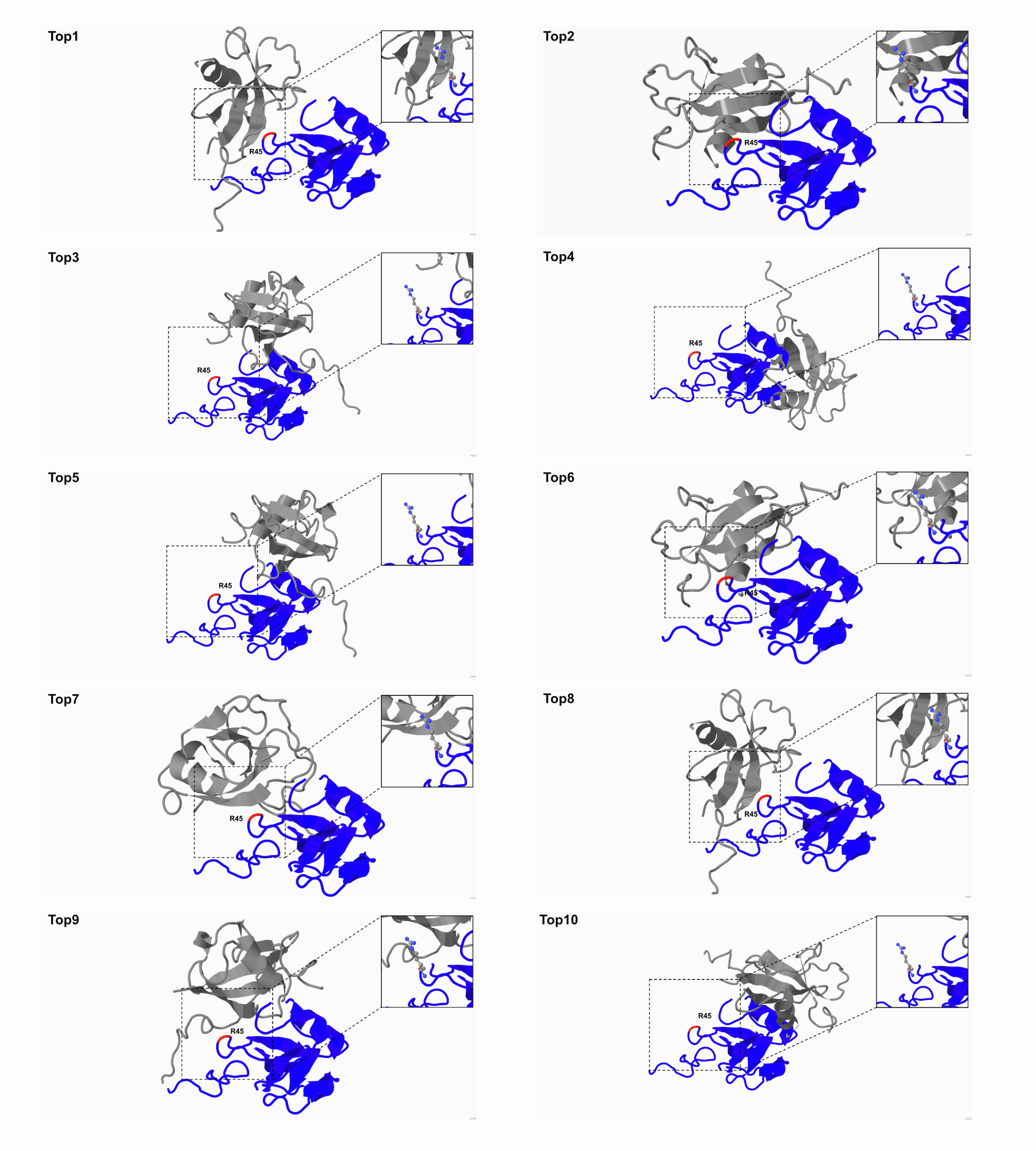

Figure S3. Top 10 docking predictions for RP1L1 and RP1 binding

The Phyre2 server generated RP1L1 (blue) and RP1 (gray) protein structures. The protein structures were visualized with Jmol (https://jmol.sourceforge.net).

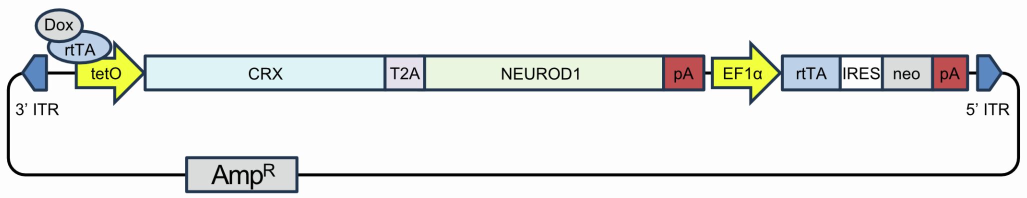

Figure S4. Schematic representations of the polycistronic piggyBac vector for CRX and NEUROD1 introduction into iPSCs

ITR, inverted terminal repeats; Dox, doxycycline; rtTA, reverse tetracycline transactivator; tetO, dox-responsive promoter; T2A, self-cleaving 2A peptide; pA, polyadenylation signal; EF1α, elongation factor 1α; IRES, internal ribosome entry site; neo, neomycin resistance gene.

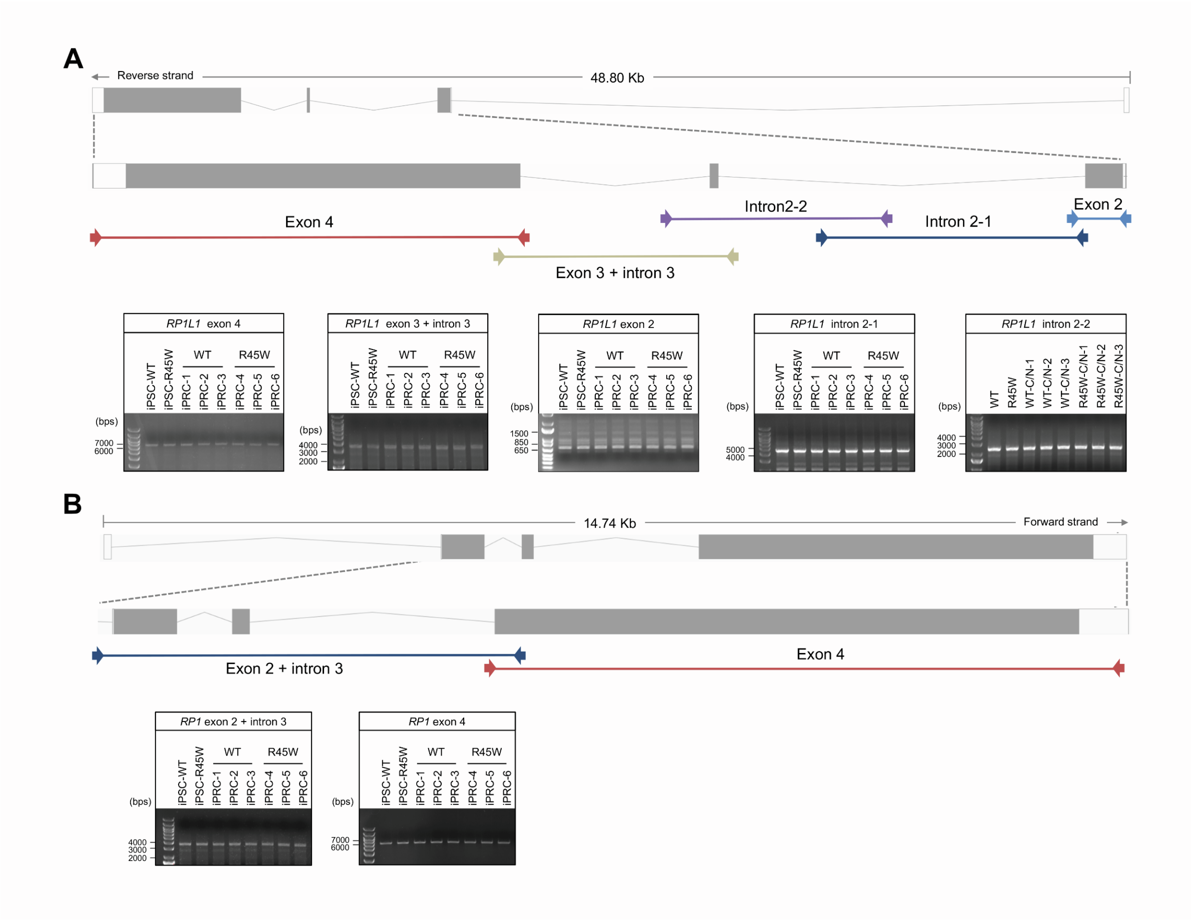

Figure S5. Confirmation of *RP1L1* and *RP1* gene integrity via endpoint PCR

Gel electrophoresis images showing the PCR products of *RP1L1* (A) and *RP1* (B) from iPSCs (induced pluripotent stem cells) and iPRCs (iPSC-induced photoreceptor-like cells). The integrity of the RP1L1 and RP1 genes was verified. The arrows highlight the positions of the primers utilized in the PCR reactions.

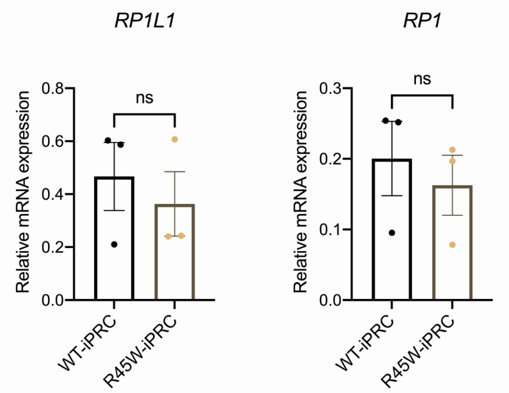

Figure S6. *RP1L1* and *RP1* transcription in iPRCs

The transcription levels of *RP1L1* and *RP1* in iPRCs were analyzed via RT–qPCR. The mRNA levels were normalized to those of *GAPDH* and measured in quadruplicate. The data are presented as the means ± SEMs (standard error of the mean). Statistical significance was assessed via Student's *t* test, with nonsignificant results indicated as "ns" (p >= 0.05).

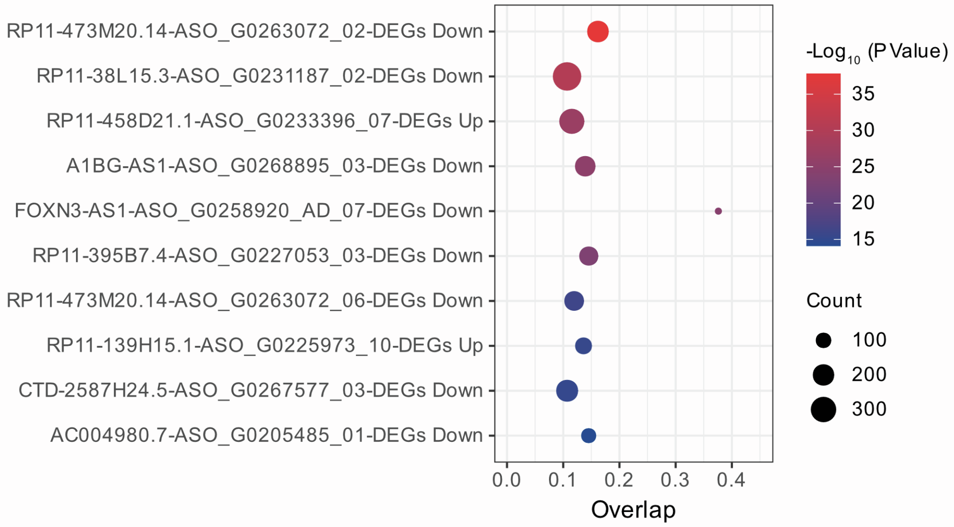

Figure S7. FANTOM6 enrichment analysis

Enrichment analysis was conducted on 1242 DEGs identified in R45W-iPRCs via the FANTOM6_lncRNA_KD_DEGs library. This analysis aimed to uncover associations between R45W and other long noncoding RNAs.

Table S1: Staging of OMD on the basis of visual symptoms and SD-OCT findings.

Family ID	Patient ID	Sex	Age at onset	Duration	Visual Symptoms		Decimal BCVA		OCT			Stage
					1st	2nd	OD	OS	IZ	EZ	Foveal Bulge	
1	KA-008	F	30	37	Reduced VA	-	0.15	0.15	Extinguished	Blurred	Present	IIb
2	KA-017	F	38	10	Reduced VA	Photophobia	0.1	0.1	Extinguished	Blurred	Present	IIb
3	KA-035	F	25	9	Reduced VA	Photophobia	0.2	0.2	Extinguished	Blurred	Present	IIb
4	KA-067	F	7	12	Reduced VA	Photophobia	0.15	0.15	Extinguished	Blurred	Present	IIb
4	KA-068	F	40	10	Photophobia	Reduced VA	0.1	0.1	Extinguished	Blurred	Present	IIb
5	KA-290	F	11	10	Reduced VA	Photophobia	0.1	0.1	Extinguished	Blurred	Present	IIb
5	KA-313	F	37	18	Reduced VA	Photophobia	0.1	0.1	Extinguished	Blurred	Present	IIb
6	SMOP-P01	F	40	17	Reduced VA	-	0.1	0.4	Extinguished	Blurred	Present	IIb
6	SMOP-P02	F	10	48	Reduced VA	-	0.2	0.15	Extinguished	Blurred	Absent	IIIa
6	SMOP-P03	F	6	12	Reduced VA	-	0.15	0.15	Extinguished	Blurred	Present	IIb
6	SMOP-P06	M	20	63	Reduced VA	-	0.15	0.15	Extinguished	Disrupted	Absent	IIIb
6	KA-014	F	18	17	Reduced VA	Photophobia	0.2	0.2	Extinguished	Blurred	Present	IIb
7	Nagoya-144-0144	F	38	2	Photophobia	Reduced VA	0.4	0.5	Extinguished	Blurred	Present	IIb
8	KINKI-070-0017	F	19	6	Reduced VA	Color vision abnormality	0.2	0.2	Extinguished	Blurred	Absent	IIIa
8	KINKI-070-0018	F	12	40	Reduced VA	-	0.1	0.15	Extinguished	Blurred	Absent	IIIa
8	KINKI-070-0019	M	8	7	Reduced VA	-	0.2	0.2	Extinguished	Blurred	Absent	IIIa
9	KINKI-002-2	F	28	15	Reduced VA	-	0.3	0.4	Extinguished	Blurred	Present	IIb
10	KINKI-030-11	M	60	10	Reduced VA	-	0.1	0.2	Extinguished	Blurred	Present	IIb
10	KINKI-030-14	M	58	4	Reduced VA	-	0.15	0.15	Extinguished	Blurred	Present	IIb
11	Nagoya-003-0003	F	42	10	Reduced VA	-	0.3	0.6	Extinguished	Blurred	Present	IIb

12	Nagoya-007-0007	M	20	29	Reduced VA	-	0.16	0.16	Extinguished	Blurred	Present	IIb
13	Nagoya-040-0040	F	39	9	Reduced VA	-	0.2	0.2	Extinguished	Blurred	Present	IIb

OMD = occult macular dystrophy; SD-OCT= spectral-domain optical coherence tomography; F = female; M = male; VA = visual acuity; OD = right eye; OS = left eye; IZ = interdigitation zone; EZ = ellipsoid zone; BCVA = best corrected visual acuity.

Table S2. Sequencing metrics for *RP1L1* and *RP1* coverage.

Metrics	*RP1L1* (pCMV-HA-N-RP1L1)	*RP1* (pCMV-Myc-N-RP1)
Contig Length (with gaps)	8,371	7,326
Contig Length (without gaps)	7,788	7,208
Average Length/Sequence	802	803
Total Sequence Length	136,368	41,764
Top Strand	75	26
Bottom Strand	95	26
Total	170	52
Average Coverage	16.29	5.7

Table S3: Results of in silico molecular genetic analysis for identified *RP1L1* variants in the pCMV-HA-N-RP1L1 (with or without R45W) plasmid and *RP1* variants in the pCMV-Myc-N-RP1 plasmid.

Nucleotide change		Amino acid change	dbSNP ID	Frequency (GnomAD)	SIFT	Polyphen2	CADD	REVEL	MetaLR	Mutation Assessor
RP1L1	c.501A>G	-	-	-	-	-	-	-	-	-
	c.665A>C	p.222H>P	rs4388421	18.61%	tolerated	possibly damaging	likely benign	likely benign	tolerated	low
	c.1242C>T	-	-	-	-	-	-	-	-	-
	c.1651C>T	p.551R>W	rs372698666	0.00%	tolerated	benign	likely benign	likely benign	tolerated	low
	c.1837C>T	-	-	-	-	-	-	-	-	-
	c.4401G>T	p.1467R>S	rs4840498	98.42%	tolerated	benign	likely benign	likely benign	tolerated	-
	c.4448C>T	p.1483A>V	rs62490855	17.88%	deleterious	benign	likely benign	likely benign	tolerated	-
	c.5530G>A	p.1884A>T	rs773601565	0.00%	tolerated	benign	likely benign	likely benign	tolerated	-
	c.5836C>A	p.1946A>E	rs11785822	92.42%	tolerated	benign	likely benign	likely benign	tolerated	-
	c.5860A>G	p.1954T>A	rs11783478	61.40%	tolerated	benign	likely benign	likely benign	tolerated	-
	c.6723A>G	-	-	-	-	-	-	-	-	-
	c.6853G>A	p.2285G>R	rs55642448	47.84%	tolerated	benign	likely benign	likely benign	tolerated	-
RP1	c.2619G>A	p.872R>H	rs444772	27.61%	deleterious	benign	likely benign	likely benign	tolerated	neutral
	c.5008G>A	p.1670A>T	rs446227	25.94%	tolerated	benign	likely benign	likely benign	tolerated	low
	c.5071T>C	p.1691S>P	rs414352	27.75%	tolerated	benign	likely benign	likely benign	tolerated	neutral
	c.5175A>G	-	-	-	-	-	-	-	-	-

dbSNP = single nucleotide polymorphism database; GnomAD = genome aggregation database.

Table S4. Information on the primers used in the experimental procedures.

Name	Sequence (5' to 3')	Application
RP1L1-R45W-F	TTTGTCCTTCCTTCTGTTCCATC	PR1L1-R45W sequencing
RP1L1-R45W-R	GCTTCTTATCAGAGCAGAGGTAGC	
RP1L1-A-EcoRI-F	ATGAGAATTCGGATGAACAGCACCCCCAGGAATG	RP1L1 cloning
RP1L1-A-R	GCCACAGGTCCTTCGAGATG	
RP1L1-B-F	TGCCAGCAAAGTGAGGTTCAAAGACTCC	
RP1L1-B-Not1-R	ATGCGCGGCCGCCTAGAAATCTAAGTCATCTTGG CCAA	
RP1-Sal1-F	ATGCGTCGACCATGAGTGATACCCCTTCTAC	RP1 cloning
RP1-Not1-R	CCCCGCGGCCGCTTATAAATCTTCTTGTTCTCT	
RP1-seq1R	GACCTCTGTCAGGTGCTGTAGAAA	RP1 sequencing
RP1-seq2F	ACGGCGAGTCCTACCTATGTTC	
RP1-seq2R	TCTCAACTGTCATAGTGCCGTCTT	
RP1-seq3F	GGAAATGCAAAGTCAGAAAGCA	
RP1-seq3R	ACTAAGGTCACACTGCCAATCAC	
RP1-seq4F	ACCTATGGAGCGAAGCAGTAATC	
RP1-seq4R	CATCTGCTGAAATAGGACTGAACC	
RP1-seq5F	CAAGTGCAATAAGTGCTGGTGTT	
RP1-seq5R	TCTTGGAAATCGTGGAATTGAG	
RP1-seq6F	GACAGCAAGCAATAAATTCCAGGT	
RP1-seq6R	GCTAAAGAATTTGCCCTGGTTG	
RP1-seq7F	GAAGTGGCATCTGGGTATTTGAG	
RP1-seq7R	GCAGCCTCTACACTTTGCCTTT	
RP1-seq8F	AATTGCCGGTTTGACAGGAG	
RP1-seq8R	ACTCTGAATGTTGACCGTGGAA	
RP1-seq9F	GATGCTCACAAGGCTACCAACA	
RP1-seq9R	TTAGGCAAAGGCCACAGGAG	
RP1-seq10F	TTCCTGTCAATGTCTGCAATACC	
RP1-seq10R	GTTTCTCCTTCATTGGTCTCCTTT	
RP1-seq11F	GGAGAGCAAGCCACTGAAGAA	
RP1-seq11R	CCCTCACAGCACTAACATCACAC	
RP1-seq12F	GCGAACTTACCCAAGAGAAAGAA	
RP1-seq12R	CCAGCAGAAATAAAGGAATGAGTG	
RP1-seq13F	CGATGGATGAACTCTCCTCTTCA	
RP1-seq13R	CCGCTGAGGTCTTGTGTATTTG	
RP1-seq14F	CAGGGAAGAGAACAATAAAGCAAG	

RP1L1-seq1F	GAGTGCTTCCTGCCCTCTGT	RP1L1 sequencing
RP1L1-seq2F	GCCTGTCCGTGGAGATGAAA	
RP1L1-seq3F	GAGAGATGCAGCCAGGACAG	
RP1L1-seq4F	AGGCAGAGACGCAAGGACAG	
RP1L1-seq5F	CTCTTCCACCCCTTCCACCT	
RP1L1-seq6F	CTCCTCGGGCTCTCTTCCTT	
RP1L1-seq7F	CCTCCTCACCCTTGGTTCTG	
RP1L1-seq8F	TATGAGTTGGCGGACGAGAC	
RP1L1-seq9F	TGACACTGGTCCCCAATCAG	
RP1L1-seq10F	GAAGAAGGGCTGCAAGAAGAG	
RP1L1-seq11F	CACAGAGGAACCCACAGAGC	
RP1L1-seq12F	ATGATGGGCCAAGAGCACAC	
RP1L1-seq13F	CGAAACCTCTCGGCCTTCTC	
RP1L1-seq14F	GCAACCAGAGGTCCCATCAA	
RP1L1-seq15F	GGGGAGACCCAGAAGACAGA	
RP1L1-seq16F	GAGGCAGAAGAGGAGGCACA	
RP1L1-seq17F	GCCCAACCAGAGTTAGAAGGTG	
RP1L1-seq18F	GGGGCCAGTGAAGGTTATGA	
RP1L1-seq19F	AGAGCTGCCCCTGAAAACCT	
RP1L1-seq20F	TGAGGATGAAGCAGAAAGAGACA	
RP1L1-seq21F	CTCGAACCTGGAGCAGTTAGC	
RP1L1-seq22F	CCCCGGAGGCAGAAAAGGAG	
RP1L1-seq23F	AGTCAGAAGGTGCAGAGGCCCAAG	
RP1L1-seq24F	GAGGCCCAGGAGGCTGAAGAGGA	
RP1L1-seq25F	CATCTGGGTGTCCGTGTTACTG	
RP1L1-seq26F	CAACCAGAGGTCCCATCAAA	
RP1L1-seq27F	GGGCATAAGTGAAAGGGGAGA	
RP1L1-seq28F	GCTCTGGGCATGAGGACAAC	
RP1L1-seq29F	GAGGCCCAGGAGGCTGAAG	
RP1L1-seq1R	GATCCCCTCGCTTGAGGAAG	
RP1L1-seq2R	GCAGGGGATTCGTCCAGATT	
RP1L1-seq3R	GGCTGACGAGTCCGAAGAAG	
RP1L1-seq4R	CCTTGCGGTAGTGAGAATGC	
RP1L1-seq5R	AAGGAAGAGAGCCCGAGGAG	
RP1L1-seq6R	CTGTGAGCAGCAGTGGCTTC	
RP1L1-seq7R	CTTCTCCTTGATGCCCCTGA	
RP1L1-seq8R	GGGACACCCTCTCCTGATTG	

RP1L1-seq9R	CACACCAGAGGAGGATGTGG	
RP1L1-seq10R	CCCTCTGCTCCTCACTGTCTCT	
RP1L1-seq11R	AGCCCTTCTCCTCCTGTTTCT	
RP1L1-seq12R	GGTCCAGCAGATCATTGTCC	
RP1L1-seq13R	AGGCTCCAGCACCATCCTAC	
RP1L1-seq14R	ATCCTCACCCTCGTCCACTC	
RP1L1-seq15R	CCCGAGTTTGGGATCTTTGT	
RP1L1-seq16R	CCTGGATCTTGGTCACCTCCT	
RP1L1-seq17R	ACCTTCTGACTCTGGCTCGTC	
RP1L1-seq18R	TCTGCCTCCTGGGCATCTAC	
RP1L1-seq19R	TCAGAAGCCTCCTCAGATTGG	
RP1L1-seq20R	CCAGTGGACTGAACGTTGCTC	
RP1L1-seq21R	GGCGCTGAAGGTCTTAAAGG	
RP1L1-seq22R	GTCCATGAGGGCGCTGAAGGT	
RP1L1-seq23R	GAGGCACGCGCTGGGAGAG	
RP1L1-seq24R	GGTAGCAGCCTCCATCTTCC	
RP1L1-seq25R	GCTTCTTATCAGAGCAGAGGTAGC	
RP1L1-seq26R	GCCTCTTCTTCTTGCTGTCCTT	
RP1L1-seq27R	GCTGGCCTCTGACAATTCCT	
RP1L1-seq28R	TCATCTTCCTGGGAGCCTTT	
RP1L1-seq29R	TGCCTCTGGGGTCTCTACATC	
RP1L1-seq30R	CGTCTGACTCTGGCTGGGCATC	
RP1L1-seq31R	AGCCTCCTGTGCCTCCTCTT	
RP1L1-seq32R	TCTGCCTCCTGGGCATCTACATCT	
RP1L1-seq33R	TCAGAAGCCTCCTCAGATTGG	
RP1L1-seq34R	TCTGCCAGCAGTTGCCCCAAG	
RP1L1-seq35R	TGGAGGAGGAAGGGCCTGTTTG	
RP1L1-seq36R	CTCCAAGTACATGGTCATTT	
PB-CRX-T2A-NeuroD1-CRX-1 F	TGGATCCGGTACCGATGATG	CRX and NEUROD sequencing
PB-CRX-T2A-NeuroD1-CRX-1 R	CTCCAGATGGACACAGTGG	
PB-CRX-T2A-NeuroD1-CRX-2 F	CCCTCCACAGATGTGTGTCC	
PB-CRX-T2A-NeuroD1-CRX-2 R	CTCTGCCCTCCAAGATCTGA	
PB-CRX-T2A-NeuroD1-NEUROD1-1 F	ACCCTGGCCCTATGACCAAA	
PB-CRX-T2A-NeuroD1-NEUROD1-1 R	CCAGGTTGGTGGTGGGTTG	

PB-CRX-T2A-NeuroD1-NEUROD1-2 F	CGCTTTGCAAGGGCTTATCC	
PB-CRX-T2A-NeuroD1-NEUROD1-2 R	CACTTTGTACAAGAAAGCTGGGT	
CRX F	CCAGTGTGGATCTGATGCACCA	RT–qPCR
CRX R	GGTACTGGGTCTTGGCAAACAG	
NEUROD1 F	GGTGCCTTGCTATTCTAAGACGC	
NEUROD1 R	GCAAAGCGTCTGAACGAAGGAG	
RCVRN F	TTCAAGGAGTACGTCATCGCC	
RCVRN R	GATGGTCCCGTTACCGTCC	
RHO F	CACCTCTCTGCATGGATACTTCG	
RHO R	ATGGGCTTACACACCACCAC	
ARR3 F	GCACAAGCTAGGGGACAATG	
ARR3 R	CCAGCCGCACATAGTCTCTC	
RP1L1 F	AAAATGCCAGGAGAAGCGAGGC	
RP1L1 R	AGACCGCGAATGGATCACACTC	
RP1 F	TCTACAGCACCTGACAGAGGTC	
RP1 R	TAAATGGCTCCCTTCCTGCCGC	
RP1L1 intron 2-1 F	ACCAGCGGGAAAAAGGTAGG	PCR
RP1L1 intron 2-1 R	AATCGCACCCACTAAACGGT	
RP11 intron 2-2 F	AGCTACCGTTTAGTGGGTGC	
RP1L1 intron 2-2 R	ACTGTTGCCTGAGACCATCG	
RP1L1 exon 2 F	CCCTCTTCCTCTGCCCTGTT	
RP1L1 exon 2 R	TTTTTCCCGCTGGTCGTGTA	
RP1L1 exon 3 + intron 3 F	CAGGGTAGAGGGGTTTCTGC	
RP1L1 exon 3 + intron 3 R	ATGGATCACACTCGGCTTGG	
RP1L1 exon 4 F	TTCCTCTTCACTGCGTGCC	
RP1L1 exon 4 R	GGCTGACCTCCGATAACCG	
RP1 exon2-intron 3 F	TCTTCTCTAGGTCTCAGCCAAAA	
RP1 exon2-intron 3 R	GAGTAAAAGGTGCCCACATCAAAA	
RP1 exon 4 F	GATGTGGGCACCTTTTACTCTTA	
RP1 exon 4 R	TTGTCCTTAGCACATTTTGATGCC	

Uncropped images

Figure 2E

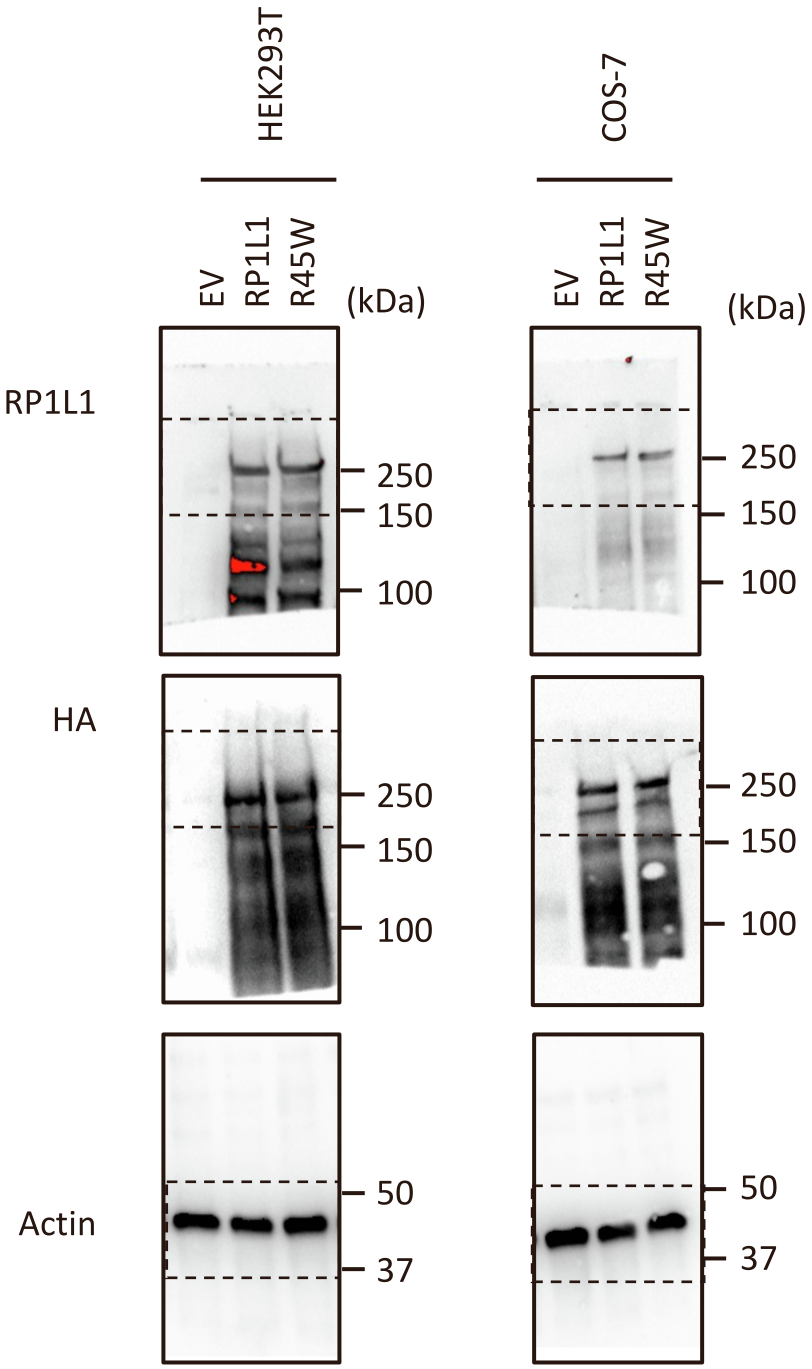

HEK293T
COS-7
EV
RP1L1
R45W
(kDa)
RP1L1
HA
Actin
250
150
100
50
37

Figure 3C

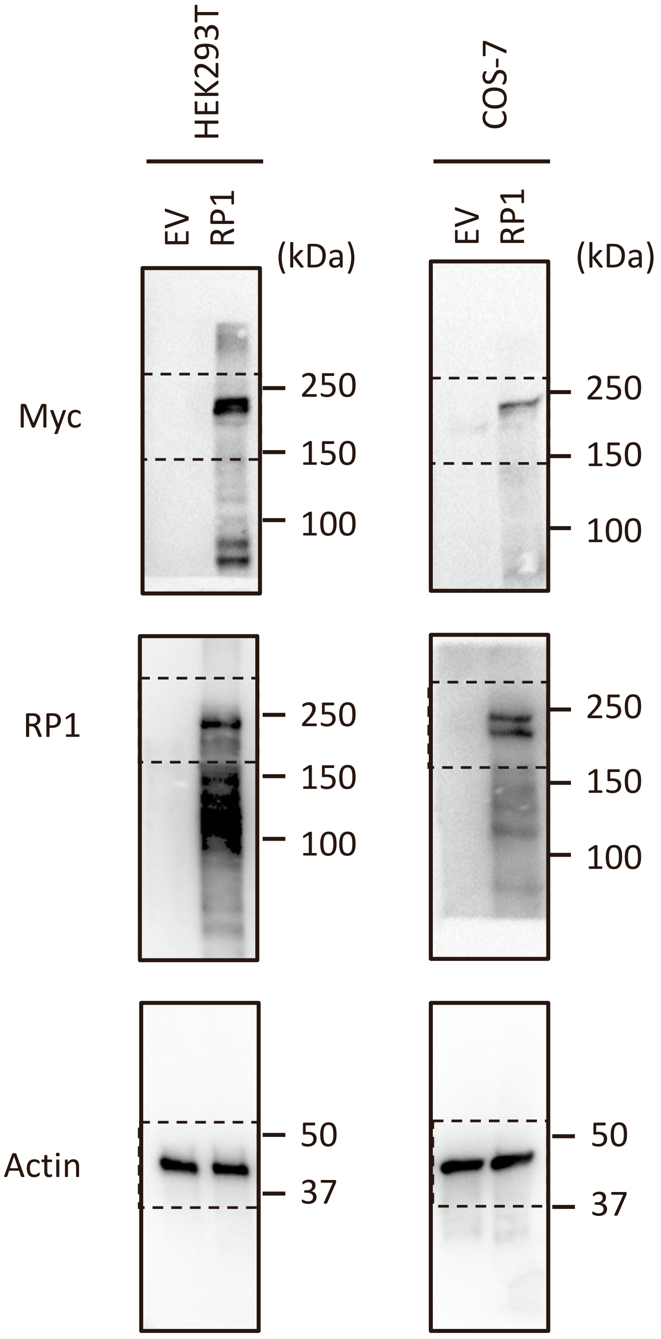

Figure 3D

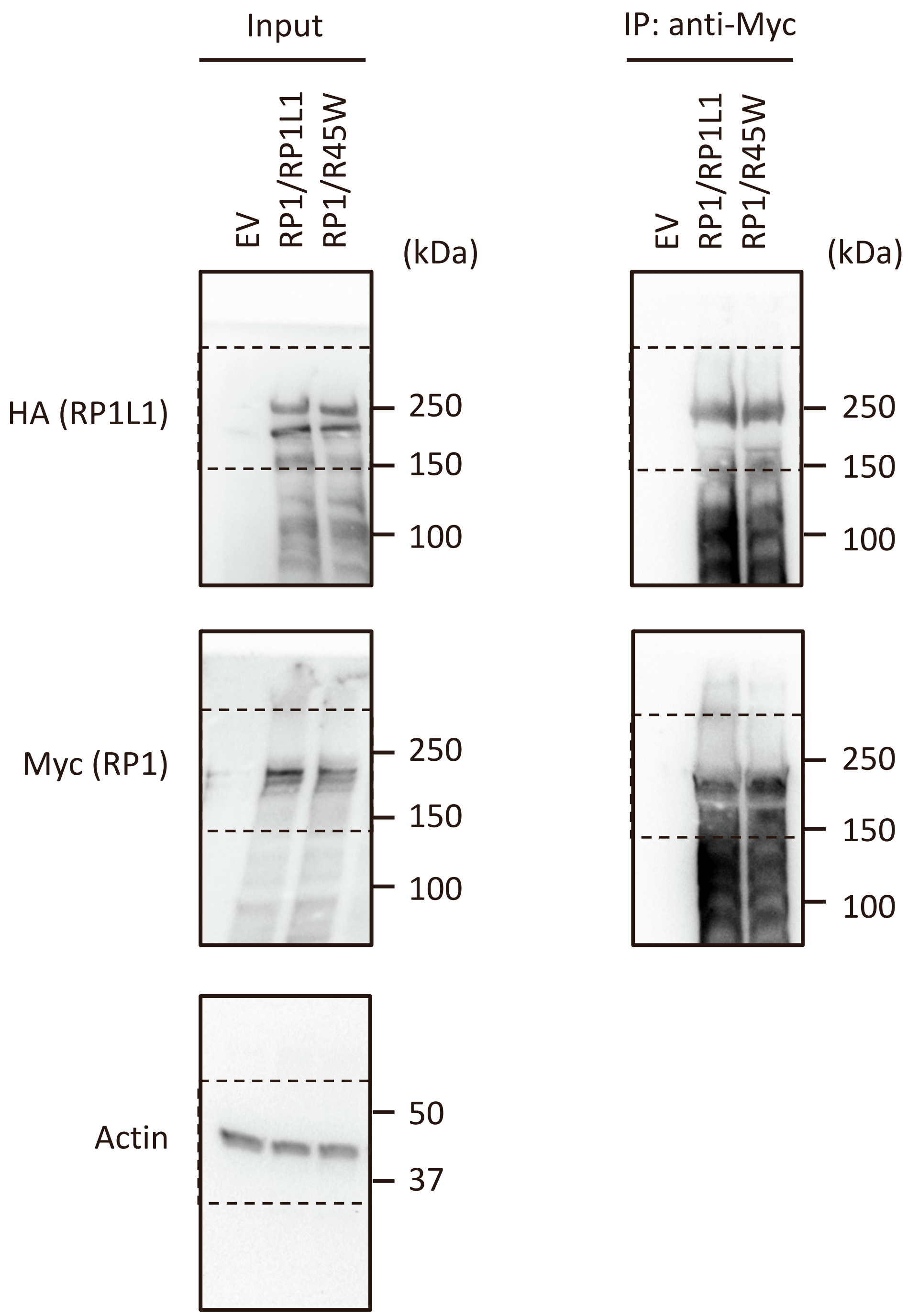
Input
IP: anti-Myc
EV
RP1/RP1L1
RP1/R45W
(kDa)
HA (RP1L1)
250
150
100
Myc (RP1)
250
150
100
Actin
50
37

Figure 4I

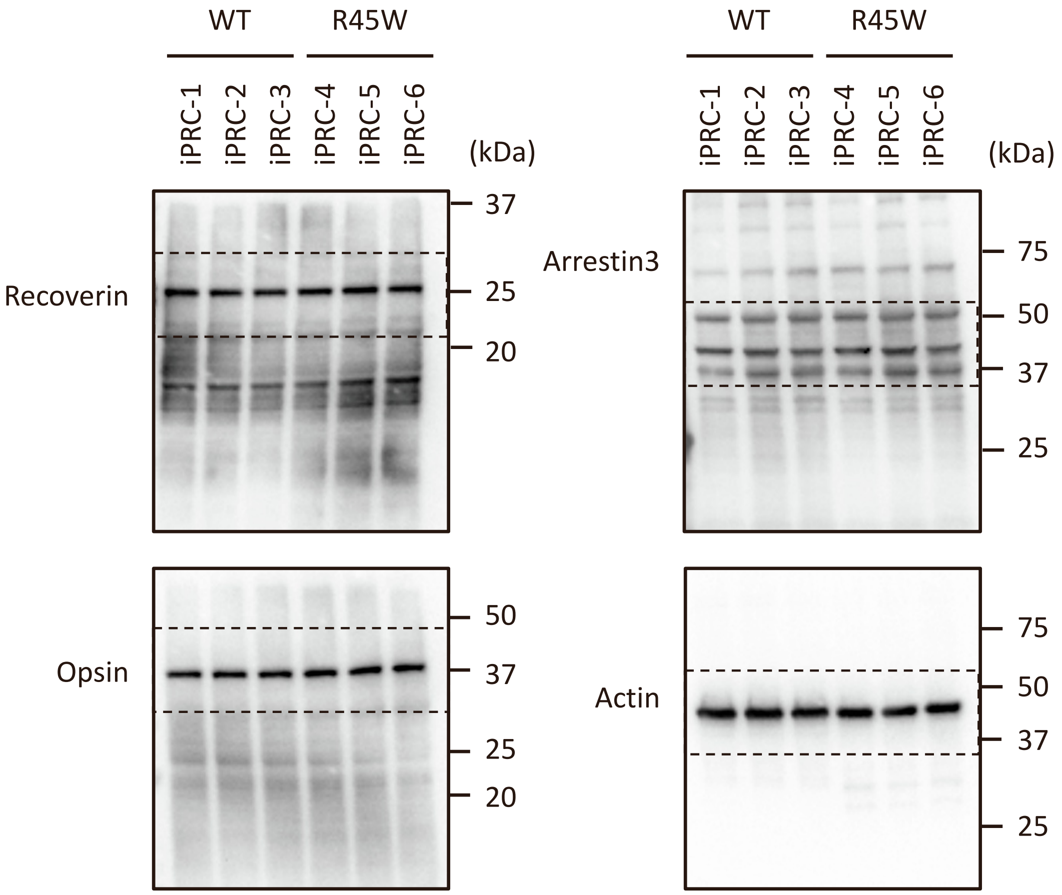

WT
R45W
iPRC-1
iPRC-2
iPRC-3
iPRC-4
iPRC-5
iPRC-6
(kDa)
Recoverin
37
25
20
Opsin
50
37
25
20
WT
R45W
iPRC-1
iPRC-2
iPRC-3
iPRC-4
iPRC-5
iPRC-6
(kDa)
Arrestin3
75
50
37
25
Actin
75
50
37
25

Figure 7C

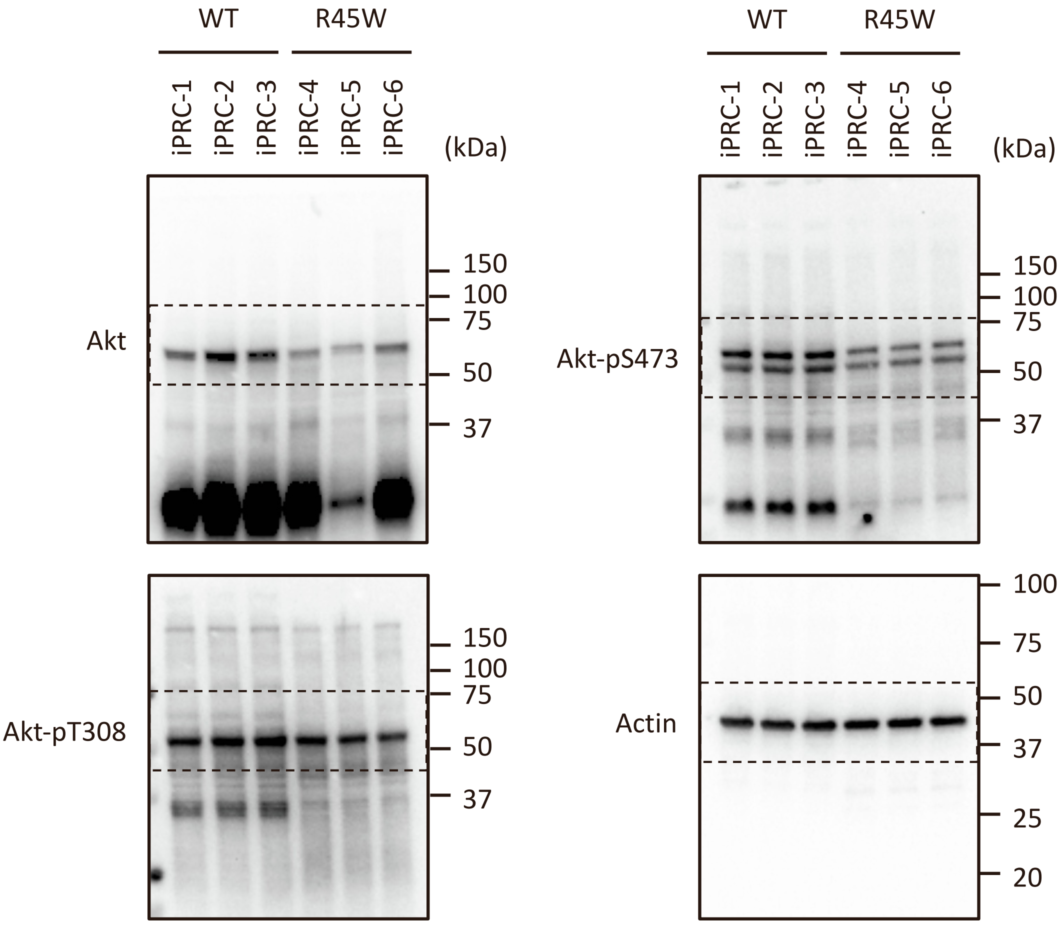
WT
R45W
iPRC-1
iPRC-2
iPRC-3
iPRC-4
iPRC-5
iPRC-6
(kDa)
Akt
150
100
75
50
37
Akt-pS473
Akt-pT308
Actin
25
20

Figure S4

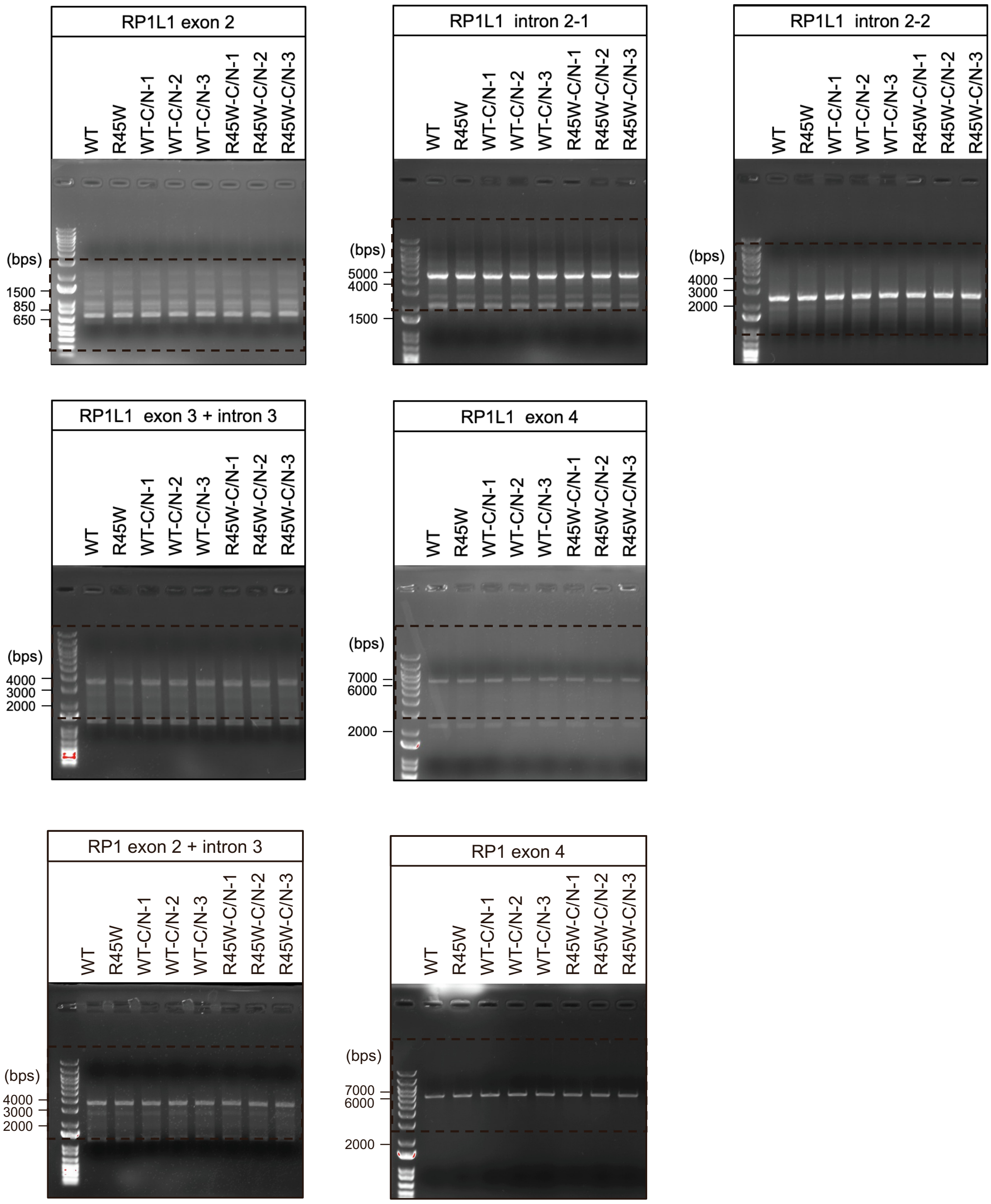
RP1L1 exon 2
WT
R45W
WT-C/N-1
WT-C/N-2
WT-C/N-3
R45W-C/N-1
R45W-C/N-2
R45W-C/N-3
(bps)
1500
850
650
RP1L1 intron 2-1
(bps)
5000
4000
1500
RP1L1 intron 2-2
(bps)
4000
3000
2000
RP1L1 exon 3 + intron 3
(bps)
4000
3000
2000
RP1L1 exon 4
(bps)
7000
6000
2000
RP1 exon 2 + intron 3
(bps)
4000
3000
2000
RP1 exon 4
(bps)
7000
6000
2000

Members of the Japan Eye Genetics Consortium:

Takeshi Iwata, Kazushige Tsunoda, Akiko Suga, Yang Pan, Kazutoshi Yoshitake, Yu Teruyama, Kazuki Kuniyoshi, Takaaki Hayashi, Shinji Ueno, Kei Shinoda, Hiroyuki Kondo, Mineo Kondo, Makoto Nakamura, Kaoru Fujinami, Shuhei Kameya, Nobuhisa Naoi, Yoshihide Hayashizaki, Yasuhiro Murakawa, Hideya Kawaji, Carninci Piero, Toshihide Nishimura, Nobuhiro Shimozawa, Yozo Miyake, Masayuki Horiguchi, Syuichi Yamamoto, Manami Kuze, Atsushi Mizota, Nobuhisa Naoi, Shigeki Machida, Yoshiaki Shimada, Hisashi Fujikado, Yoshihiro Hotta, Masayo Takahashi, Kiyofumi Motiduki, Akira Murakami, Susumu Ishida, Mitsuru Nakazawa, Teruhisa Hatase, Kazuo Tsubota, Akiko Maeda, Atsuhiro Tanigawa, Syuji Yamamoto, Hiroyuki Yamamoto, Tetsuju Sekiryu, Kenji Kashiwagi, Takeo Fukuchi, Atsuhshi Hayashi, Katsuhiro Hosono, Keisuke Mori, Koichi Furuya, Keiichiro Suzuki, Yasuo Yanagi, Natsuko Nakamura.
